# Cell shape information is transduced through tension-independent mechanisms

**DOI:** 10.1038/s41467-017-02218-4

**Published:** 2017-12-15

**Authors:** Amit Ron, Evren U. Azeloglu, Rhodora C. Calizo, Mufeng Hu, Smiti Bhattacharya, Yibang Chen, Gomathi Jayaraman, Sunwoo Lee, Susana R. Neves-Zaph, Hong Li, Ronald E. Gordon, John C. He, James C. Hone, Ravi Iyengar

**Affiliations:** 1 Department of Mechanical Engineering, Columbia University, New York, NY 10027 USA; 2 Department of Pharmacological Sciences and Systems Biology Center New York, Icahn School of Medicine at Mount Sinai, New York, NY 10029 USA; 3 School of Electrical and Computer Engineering, Cornell University, Ithaca, NY 14853 USA; 4 Department of Microbiology, Biochemistry and Molecular Genetics, Rutgers University – New Jersey Medical School, Newark, NY 07103 USA; 5 Department of Pathology, Icahn School of Medicine at Mount Sinai, New York, NY 10029 USA; 6 Division of Nephrology, Department of Medicine, Icahn School of Medicine at Mount Sinai, New York, NY 10029 USA

## Abstract

The shape of a cell within tissues can represent the history of chemical and physical signals that it encounters, but can information from cell shape regulate cellular phenotype independently? Using optimal control theory to constrain reaction-diffusion schemes that are dependent on different surface-to-volume relationships, we find that information from cell shape can be resolved from mechanical signals. We used microfabricated 3-D biomimetic chips to validate predictions that shape-sensing occurs in a tension-independent manner through integrin β_3_ signaling pathway in human kidney podocytes and smooth muscle cells. Differential proteomics and functional ablation assays indicate that integrin β_3_ is critical in transduction of shape signals through ezrin–radixin–moesin (ERM) family. We used experimentally determined diffusion coefficients and experimentally validated simulations to show that shape sensing is an emergent cellular property enabled by multiple molecular characteristics of integrin β_3_. We conclude that 3-D cell shape information, transduced through tension-independent mechanisms, can regulate phenotype.

## Abstract (web summary)

It is not known whether the shape of a cell can regulate cellular phenotype independently. Here, the authors show that culturing kidney podocytes or smooth muscle cells on 3-D biomimetic surfaces results in phenotypic changes and that cell shape is sensed by integrin β_3_ in a tension-independent manner.

## Introduction

It has been empirically known that the in vivo shape of cells is an indicator of health or disease, and this is one of the foundations for clinical pathology. Cell shape is often seen as an as an output of mechanotransduction^1,2^, whereby mechanical forces transmitted through the extracellular matrix (ECM) are converted to biochemical signals that modulate the cytoskeletal structure^3–5^. However, many other factors, including interactions with the ECM and chemical signals such as autocrine and paracrine factors, also regulate cell shape. Additionally, different lipid microdomains such as lipid rafts can affect cell shape^6^. Hence, shape can be an integrative repository of information from multiple physical and chemical sources operating in different time domains. In this study, we ask whether information stored in shape can regulate cell phenotype, in tandem with other well-studied factors such as chemical signals (growth factors, morphogens) and physical information (substrate stiffness)^7–11^. While shape modulates transmembrane chemical signaling^12^, can cell shape on its own, independent of tension, be a source of information? This general question raises two specific questions, as follows: (i) how is the information stored in cell shape retrieved? and (ii) how does this information contribute to cellular phenotype?

We studied two morphologically different cell types: human kidney podocytes and vascular smooth muscle cells (SMCs). In vivo, podocytes possess a branched morphology with projections called foot processes, which interdigitate to form the slit diaphragm^13^, an intercellular junction in which specific proteins create a porous filtration barrier^14^; failure to maintain the branched morphology and the slit diaphragm leads to kidney disease^15^. Mature SMCs show an elongated spindle morphology and express specific contractile proteins associated with their ability to exhibit a contractile phenotype^16^. Similar to podocytes, when cultured in vitro or under in vivo conditions of vascular injury, SMCs adopt a proliferative phenotype with significant changes in cell shape and decreased expression of contractile proteins^17^.

We used microfabrication to construct 3-D single-cell micropatterns representing simplified versions of the in vivo morphology of podocytes and SMCs. In both types, cells in the shapes showed marked phenotypic changes, as measured by expression levels of physiologically important proteins and localization of these proteins to the appropriate subcellular compartments. We used a reaction-diffusion model to understand the modulation of membrane-based signaling by shape, and an optimal control theory model to resolve the effects of cell shape and intracellular tension. Our theoretical model was experimentally validated in podocytes, which show shape-dominated phenotype, and in fibroblasts, which show tension-dominated phenotype. Using proteomics and functional assays, we found that integrin β_3_ and its binding partners from the ezrin–radixin–moesin (ERM) family mediate the transduction of shape signals.

## Results

### Cell shape enables a differentiated phenotype in podocytes

**Fig. 1:**
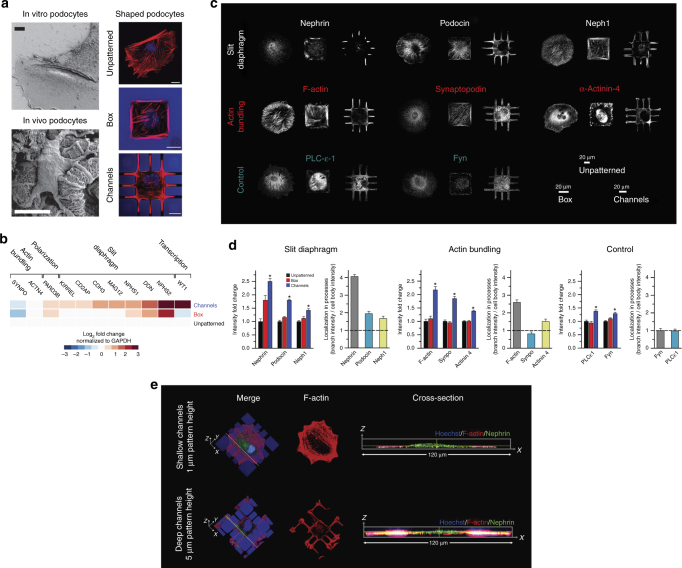
Podocytes differentiate in response to shape signals. **a** (Left) Scanning electron micrograph of in vivo podocytes showing distinct processes that branch out of a central cell body; (Right) representative images of cells cultured on unpatterned glass, box, and channel micropatterns of the 3-D biochips. Cells were stained for F-actin (red) and nuclei (blue). All scale bars are 20 μm. **b** mRNA expression levels measured by RT-PCR for physiologically essential proteins in podocytes revealed an increase in expression of nine out of eleven transcripts for cells plated on the channel micropatterns with a median increase of 87% (mean fold change of 2.8 ± 0.9; *p* < 0.05 vs. UNP; one-way ANOVA followed by a post hoc Tukey test). Cells in box patterns showed a median change of 7% with an increase in six and a decrease in five transcripts (mean fold change of 1.4 ± 0.3; *p* = ns vs. UNP). Heatmap represents the average expression levels from four independent experiments with two slides in each group. **c** Representative immunofluorescence images of physiologically essential proteins in podocytes plated on unpatterned and patterned surfaces. The proteins are organized into “slit diaphragm proteins” nephrin, podocin, and neph1 (upper row), “actin-bundling proteins” F-actin, synaptopodin, and α-actinin-4 (middle row), and “control proteins” phospholipase-C-ε and Fyn (lower row). **d** Summary of whole-cell fluorescence intensity fold change and localization ratio (ratio of fluorescence intensity within peripheral processes vs. cell body) in micropatterned podocytes. Values given as mean ± SEM; *n* = 80, chosen randomly from eight different slides cultured independently at different times (*p < 0.01 vs. UNP; one-way ANOVA followed by a post hoc Tukey test). **e** Representative immunofluorescent confocal volume scans of podocytes cultured on shallow (1 μm) and deep (5 μm) channel patterns showing nephrin (green), F-actin (red) and nuclei (blue). On biochips with shallow micropatterns, cells did not produce high aspect ratio processes enriched for nephrin. In contrast, clear enrichment was observed in deep channels. Hence, 3-D shape is necessary for the localization phenotype

To determine whether confining podocytes to physiological shapes upregulates the expression of genes relevant to in vivo podocyte function, we cultured human podocytes on 3-D engineered biochips with a simple approximation of the in vivo cell shape. These consisted of arrays of boxes (that mimic the cell body) connected by protruding channels (that correspond to primary processes), plus control surfaces consisting of either boxes or unpatterned glass. Conditionally immortalized human podocytes^18^ were plated on biochips and cultured for 5 days; the coverslips were not coated with any ECM proteins. Shape compliance was excellent even with long-term culture; actin staining showed that cells fully complied with the square/box micropatterns and put out peripheral processes on the biochips (Fig. 1a and Supplementary Fig. 1). This allowed for multiple assays of phenotype as described below.

Phenotypic state was first assessed using RT-PCR to quantify mRNA levels of eleven genes associated with physiological function, selected using gene ontology terms related to glomerular epithelial differentiation and physiology^19–22^. Compared to unpatterned cells, podocytes cultured on 3-D biochips showed a significant increase in the expression of major differentiation markers WT1, DDN, NPHS1, NPHS2, MAGI2, CDH3, CD2AP, KIRREL, and PARD3B (*p* < 0.05 vs. unpatterned, *n* = 4, one-way ANOVA), whereas podocytes on box micropatterns showed little or no change (Fig. 1b). These eleven physiologically relevant mRNAs are key for wide variety of functions (Fig. 1b). On channel micropatterns, average fold-change was 2.8 ± 0.9 (mean ± SD) vs. the unpatterned control cells, whereas on boxes it was 1.4 ± 0.3 (*p* = ns, one-way ANOVA).

We next used quantitative immunofluorescence to measure both the expression and localization of three classes of proteins: (i) key slit diaphragm proteins (nephrin, neph1, and podocin) that localize to foot processes in functionally differentiated podocytes^20^; (ii) actin-bundling-related proteins related to formation of foot processes (F-actin, synaptopodin, and α-actinin-4); and (iii) non-spatial “control” proteins phospholipase-C and Fyn (Fig. 1c). Overall expression was quantified by whole-cell fluorescence intensity divided by the spreading area of the cell onto the micropattern. The localization ratio was used only for cells plated on channel micropatterns and defined as the intensity per unit area within the channels divided by the intensity within the main boxes (i.e., branches vs. cell body). Quantitative analyses reveal that cells plated on the channel micropatterns showed increased expression of all six measured slit diaphragm and actin-bundling proteins compared to unpatterned cells, while control proteins showed no increase (Fig. 1d). In contrast, only nephrin levels increased on boxes, indicating the importance of physiological shape as opposed to spurious effects from micropatterning. In addition, all of the measured slit diaphragm proteins and two of the actin-bundling proteins showed localization into peripheral processes, while the control proteins did not. This is the “localization phenotype” required for appropriate physiological function. Podocytes on the channel micropatterns developed a median of four peripheral processes per cell, of which roughly half were “specialized” processes showing increased concentration of slit diaphragm proteins (Supplementary Fig. 2). The ratio of specialized to total number of processes was similar for all slit diaphragm proteins. Cell type selectivity of this shape-specific localization response was evaluated using neonatal rat cardiac fibroblasts and human dermal fibroblasts. While both cells conformed to shape constraints and established processes (Supplementary Fig. 3), neither cell type expressed observable levels of nephrin nor increased levels of F-actin inside these processes (Supplementary Fig. 4).

One of the unique advantages of our photoresist-based biochips is the ability to generate 3-D features that guides cell shape without any ECM coating over extended time periods. Accordingly, we saw that the 3-D shape of the micropatterns was critical in driving process specialization. When the heights of the micropatterns were lowered from deep (5 μm) to shallow (1 μm), a significant reduction in geometric compliance was observed after five days of culture (Fig. 1e). This reduction was accompanied by the loss of branched geometry, whereby cells still spread through the channels, but failed to produce specialized peripheral processes with high aspect ratios that were enriched for slit diaphragm proteins. This showed that 3-D shape constraint, similar to a hand-in-glove-fit, is necessary for the manifestation of the differentiated state. Note that podocytes in shallow micropatterns lost nephrin enrichment even though they were still spreading along the channel surface (i.e., bottom surfaces of cells in shallow micropatterns were similar to those in deep ones). The only difference between the two populations was the constraint of processes along the third dimension (i.e., height); thus, mimicking the packing effect a podocyte encounters in the tissue environment. These findings provide support for the hypothesis that 3-D cell shape provides information that is selectively recognized by the relevant cell type.

### Theoretical basis for resolving shape and tension signals

The shape of the luminal cell surface has been shown to modify intracellular tension carried by stress fibers^6^. The requirement for 3-D shape conformance that is indifferent to luminal cell surface led us to question if cell shape could be a source of information independent from cellular tension, and if so, whether we could resolve these two signal sources. As shown in Supplementary Fig. 5, the localization phenotype can be modulated by different types of extracellular signals depending on the complexity of the shape and the cell type. Therefore, we utilized a 3-D reaction-diffusion model to better understand the quantitative flow of information from cell shape and mechanical tension (see Supplementary Note 1 for detailed derivation). We assumed that information originates at transmembrane receptors that are either independent of tension (shape receptors) or activated by tension (tension receptors), and that their operation is described by reaction-diffusion in domains within limited volumes^23^. We considered geometries with increasing surface area to volume ratios (SA/V), from a perfect sphere to a complex polygon star (Supplementary Note 1). We determined how the effective forward rate of the receptors for shape or tension would change as the SA/V ratio increased by computing the forward rate, *K*_a_, of a given geometry with respect to the reference *K*_a_ of a sphere.

**Fig. 2:**
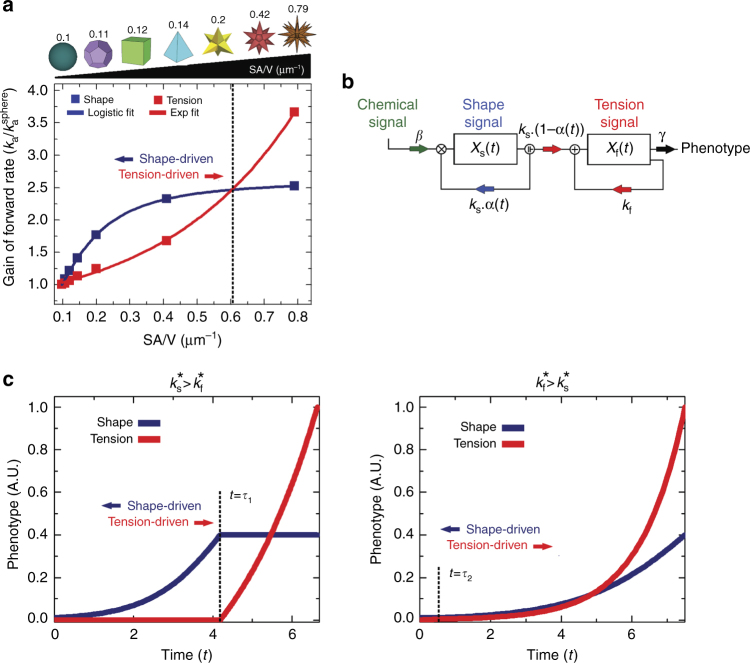
Multiple types of signals can regulate cellular phenotype. **a** Tension- (red) and shape (blue)-dependent association rate constants (*K*_a_) as functions of SA/V ratio based on a series of 3-D geometries. As the SA/V gets higher, shape signals are dominant until around SA/V ≅ 0.6 when the system becomes tension-driven. The term $K_{\mathrm{a}}/K_{\mathrm{a}}^{{\mathrm{sphere}}}$ represents the relative change with respect to a spherical reference. **b** Control state diagram for shape and tension-driven phenotypes. The state variables *X*_s_(*t*) and *X*_f_(*t*) represent phenotypic contributions of the shape and tension signals, respectively. *k*_s_ and *k*_f_ represent the rates by which shape and tension signals alter the phenotype, respectively. *γ* is the natural decay (degradation) rate of the phenotype. *β* represents the probability of chemical induction via other extracellular ligands. *α*(*t*) is the control function that represents the probability of the cell to react to shape signals, and [1−α(*t*)] is the probability to react to tension signals. **c** Phenotypic state as a function of time depicting optimal solution for the dynamics of shape- (blue) and tension- (red) driven phenotypes. When $k_{\mathrm{s}}^*  >k_{\mathrm{f}}^*$, shape cues are dominant in driving phenotype, whereas tension becomes the main driver when $k_{\mathrm{s}}^* < k_{\mathrm{f}}^*$

In this model, the activation of shape receptors is governed by the ratio between the diffusion and reaction components between the 2-D cell membrane and the 3-D cytoplasmic volume, i.e., Thiele modulus. As the shape becomes more complex, the effective forward rate constant of shape receptors increases logistically with the SA/V ratio (Fig. 2a). The decrease in local volume with increasing curvature leads to signaling microdomains^8^, a phenotype solely controlled by variations in shape. The effect of tension was modeled using structural mechanics to calculate the intracellular mechanical stress associated with the activation of mechanoreceptors throughout the plane of the membrane. The interaction between the tensile force and membrane mechanoreceptors was described by the catch-bond mechanism^24^, in which the rate constant increases exponentially with force.

Shape-driven effects on *K*_a_ increase slowly with SA/V and eventually saturates as the reaction-to-diffusion ratio plateaus with increasing membrane area (Supplementary Note 2), whereas the tension effects are more dominant for high SA/V ratios (>0.6) due to the exponential force-dependence. These results indicate that as the shape becomes more complex (i.e., SA/V increases), the regulation of receptor activity shifts towards a tension-driven system. Cell type and density of specific receptors should control the transition between the drivers of receptor activation: one cell type can show a transition point between simple shapes (e.g., square to rhombus), while another cell type may show a transition only in highly complex shapes (e.g., star).

We used optimal control theory^25^ to develop a framework to understand the dynamics by which shape and mechanical tension signals could regulate phenotype separately; where a dynamic control function *α*(*t*) regulates the evolution of state variables to achieve a desired goal^26,27^. The model assumes that for a given chemical background signal, a cell is exposed to two types of signals that affect its phenotype: shape and tension (Fig. 2b), where the state variables *X*_s_(*t*) and *X*_f_(*t*) describe the phenotypic contribution of these respective signals. Optimization seeks to find control parameter *α*(*t*) that minimizes time *t* to achieve a mature phenotype $\bar X\left( t \right) = \left[ {X_{\mathrm{s}}\left( t \right),X_{\mathrm{f}}\left( t \right)} \right]$. The phenotype is determined based on *α*(*t*) that describes the probability of the cell to react to shape or tension signals (see Supplementary Note 3 for complete derivation). The dynamics of phenotypic states are described using the rate equations:

1 $$\dot X_{\mathrm{s}}\left( t \right) = \alpha \left( t \right)\beta k_{\mathrm{s}}X_{\mathrm{s}}\left( t \right)\left( {\frac{{c - X_{\mathrm{s}}\left( t \right)}}{c}} \right)$$

2 $$\dot X_{\mathrm{f}}\left( t \right) = \left( {1 - \alpha \left( t \right)} \right)\beta k_{\mathrm{s}}X_{\mathrm{s}}\left( t \right)\left( {\frac{{c - X_{\mathrm{s}}\left( t \right)}}{c}} \right) + X_{\mathrm{f}}\left( t \right)\left( {k_{\mathrm{f}} - \gamma } \right),$$

where $k_{\mathrm{s}}\left[ {1/{\it{s}}} \right]$ and $k_{\mathrm{f}}\left[ {1/{\it{s}}} \right]$ are the rates by which the shape *X*_s_(*t*) and tension *X*_f_(*t*) phenotypes are changed; *γ*[1/*s*] is the natural decay (degradation) rate of phenotype. Since biochemical signals are known to be modulated by cell shape^8^, we include a normalized interdependence factor, *β*, that governs the relationship between shape and the biochemical signals. When *β* = 0, shape does not modulate biochemical signals (well-stirred system), and when *β* = 1, the shape effect is completely independent of biochemical signals. Since any phenotypic capacity is limited (i.e., a given cell cannot change phenotype unrestrictedly), we include parameter, *c*, the carrying capacity, which is the maximum sustainable phenotypic output (as measured by expression or localization) for a given cell.

The solutions for the above set of equations are plotted in Fig. 2c for cases: $k_{\mathrm{s}}^*  >k_{\mathrm{f}}^*$ and $k_{\mathrm{s}}^* < k_{\mathrm{f}}^*$, where $k_{\mathrm{s}}^* = k_{\mathrm{s}}\beta$, and $k_{\mathrm{f}}^* = \left( {k_{\mathrm{f}} - \gamma } \right)$. We can identify two distinct phases for each plot, separated by a time constant $\tau (k_{\mathrm{s}},k_{\mathrm{f}}).$ This phenomenon is due to the inherent nature of the optimal control function changing from *α*(*t*) = 1 to *α*(*t*) = 0 across *t* = *τ*; this type of behavior is known as bang-bang control^25^; when *t* < *τ*, phenotype is dominated by the logarithmic rise of shape signals, while the exponentially increasing tension signals take over for *t* > *τ*. For $k_{\mathrm{s}}^*  >k_{\mathrm{f}}^*$, the shape effect dominates early and then saturates, while for $k_{\mathrm{s}}^* < k_{\mathrm{f}}^*$, the crossover happens very early and tension is the dominant driver of phonotype throughout. The relative values of *k*_s_ and *k*_f_ depend on both the cell type and SA/V ratio (Fig. 2a).

### Cell shape signals through integrin β_3_ pathway

**Fig. 3:**
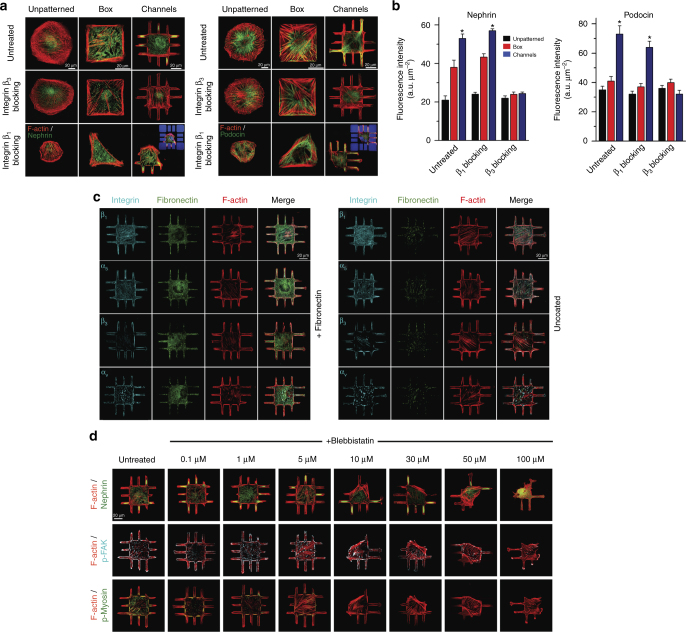
Integrin β_3_ controls shape-driven phenotype in podocytes. **a** Representative images of podocytes plated on unpatterned and micropatterned surfaces, and stained either for (left) nephrin (green) and F-actin (red), or (right) podocin (green) and F-actin (red). Both podocin and nephrin were highly localized within peripheral processes. Treating cells with integrin β_3_ blocking antibodies abolished the localization effect. Podocytes treated with integrin β_1_ blocking antibodies exhibit limited spreading; however, podocin and nephrin phenotype was relatively unaffected (insets show the autofluorescent patterns for clarity). **b** Quantitative analysis of whole-cell nephrin and podocin intensities in patterned and unpatterned podocytes treated with either β_1_ or β_3_ blocking antibodies. Values given as mean ± SEM; *n* = 80 chosen randomly from eight different slides cultured independently (*p < 0.01 vs. UNP; one-way ANOVA followed by a post hoc Tukey test). **c** No significant differences in integrin expression were observed between fibronectin coated and uncoated surfaces; spatial integrin expression did not depend on the ECM coating in 3-D micropatterned podocytes. Integrin β_1_, α_5_, β_3_, and α_v_ (cyan, stained independently), fibronectin (green), and F-actin (red) in podocytes plated on channel surfaces with and without fibronectin coating. **d** Podocytes were treated with varying concentrations of blebbistatin for 12 h prior to fixation and stained for (top) nephrin (green) and F-actin (red), (middle) p-FAK (cyan) and F-actin (red), or (bottom) p-myosin (green) and F-actin (red). Phospho-myosin intensity decreased gradually with increasing blebbistatin concentration, whereas recruitment of p-FAK to focal adhesions was not affected by blebbistatin up to 10 μM

Since integrins play a critical role in regulating shape-driven changes in phenotype^1^, we hypothesized that specific integrin isoforms may act as “effective” receptors for shape signaling. To test this hypothesis, we used functional antibodies to block integrin β_1_ and β_3_ activation in micropatterned and unpatterned podocytes and quantified the phenotypic state by measuring nephrin and podocin localization (Fig. 3a, b). Blocking β_3_ integrin did not affect morphology (Supplementary Fig. 6), but prevented localization of nephrin and podocin within peripheral processes (Fig. 3b and Supplementary Fig. 7). No significant differences were observed in unpatterned or box micropatterned cells. Blocking integrin β_1_ reduced cell spreading. Since formation of processes was limited, localization effects were not always observed, but expression of nephrin and podocin remained enhanced in the channel-shaped cells. When a lower dose of integrin β_1_ blocking antibodies was used (0.1 mg/ml), localization ratios were similar to untreated cells (Supplementary Fig. 7).

To further understand the role β_3_ and other integrin isoforms in transducing signals from cell shape, we imaged β_1_, α_5_, β_3_, and α_v_ distribution on micropatterned and unpatterned podocytes with and without fibronectin coating, as shown in Fig. 3c (see Supplementary Figs. 8–10 for validation of antibody specificity). In micropatterned podocytes, β_1_ and α_5_ were homogenously distributed, with little colocalization with fibronectin. In unpatterned cells, β_1_ and α_5_ demonstrated punctate patterns on uncoated surfaces with little colocalization with fibronectin (Supplementary Fig. 11); on fibronectin, β_1_ colocalized with fibronectin, while α_5_, which is known to form fibrillar adhesions^28^, was expressed mainly within the cell epicenter. β_3_ and α_v_ showed strong colocalization with actin bundles and were mainly expressed at cellular peripheries on coated or uncoated surfaces. On fibronectin surfaces, β_3_ formed larger focal adhesions (FAs), while α_v_ mainly clustered along the cell periphery and FAs^28,29^.

We then examined the effect of integrin β_1_ and β_3_ on the spatial arrangement and maturation of FAs on micropatterned and unpatterned podocytes by imaging active focal adhesion kinase, p-FAK (Supplementary Fig. 12). FAs on micropatterned podocytes were larger, more elongated (i.e., anisotropic), peripherally oriented, and aligned with actin bundles. On unpatterned cells, FAs were small and mainly formed at the tips of actin fibers. Blocking of integrin β_3_ reduced FA area and anisotropy while blocking integrin β_1_ had no effect on FA area. These observations suggested that β_3_ activity affects signal flow from cell shape to FAs.

Since FA assembly and maturation is also known to increase with cytoskeletal tension^30,31^, we examined FA morphology and the localization phenotype in micropatterned podocytes treated with non-muscle myosin inhibitor, blebbistatin. We tested a range of blebbistatin concentrations using unpatterned podocytes (Supplementary Figs. 13–14) and determined that significant disruption of FA maturation and force generation was observed with minimal shape change at 10 μM (Supplementary Fig. 15). Micropatterned podocytes were then treated with 10 μM blebbistatin and stained for p-FAK, F-actin, p-myosin, and nephrin (Fig. 3d, quantification in Supplementary Fig. 16). We saw that cell morphology, FA maturation, and nephrin localization showed little to no change (~ 25% decrease vs. untreated cells). At 100 μM blebbistatin, cell spreading, FA morphology, and F-actin intensity showed dramatic change, which led to a severe reduction in nephrin localization. The localization however, was found to be unaffected when the cells were plated on fibronectin-coated micropatterns prior to the blebbistatin inhibition (Supplementary Figs. 17–18). Here, the strong adhesion between cells and the ECM prevented reduced spreading, which allowed proper nephrin localization even when contractile tension was abolished. Taken together, these findings suggest that a low level of p-myosin activity (~ 30% vs. untreated at 10 μM blebbistatin) is sufficient to maintain FA maturation and phenotypic switching in podocytes (Supplementary Fig. 19). The correlation between FA maturation and F-actin density within the adhesion sites indicates that in micropatterned podocytes, FA maturation and morphology are regulated more by stress fiber architecture and less by contractile tension (Supplementary Fig. 20), which was shown to control assembly and maturation of FAs^32,33^.

To validate the specificity of our observations, we repeated the tension inhibition using a second inhibitor, namely the Rho kinase inhibitor, Y-27632 (Supplementary Figs. 21–25). For this inhibitor, the optimal concentration was ~ 5 μM where cell shape and FAs morphology were preserved while tension was abolished. Nevertheless, nephrin localization within channels was found to be significant with Y-27632 treatment, even at 30 μM. As with blebbistatin, these results support the finding that podocytes with physiological shapes are able to differentiate with substantially reduced intracellular tension.

### Experimental validation of control theory predictions

**Fig. 4:**
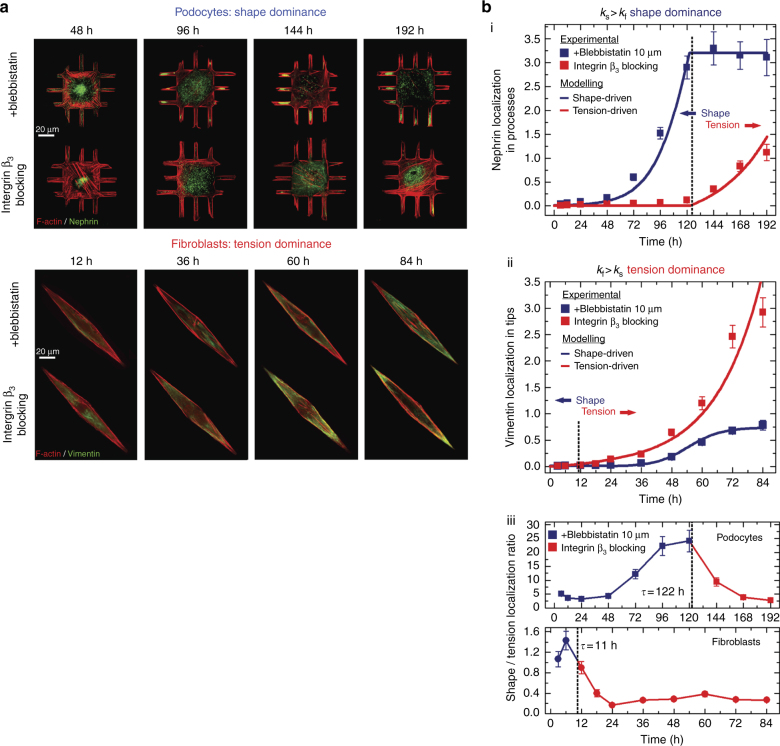
Experiments agree with the optimal control theory predictions. **a** Representative immunofluorescence images showing the dynamics of (top) nephrin localization in podocytes, and (bottom) vimentin localization in fibroblasts. Cells were plated on channel or rhombus micropatterns, treated with integrin β_3_ blocking antibodies or blebbistatin, fixed at given time points, and stained for nephrin or vimentin (green) as well as F-actin (red). **b** Experimentally measured localization dynamics in podocyte and fibroblasts for (i) nephrin (podocytes) and (ii) vimentin (fibroblasts) were fitted with the temporal order predicted from the optimal control solution (blue and red lines). Both cells show an excellent fit with the control models. A clear shape-tension transition is revealed which confirm the predicted strategy that cells initially respond to shape signals followed by a transition to a tension-driven mechanism. (iii) The localization ratio between shape and tension signals reveals how well the predicted switching time, *t* agrees with the measured dynamics. Experimental values are given as mean ± SEM; *n* = 20 cells per timepoint, chosen from four different slides, cultured independently

The optimal control theory solution presented in Fig. 2c predicts that cellular phenotypes can be regulated independently by shape and tension signals, and that a transition between states is determined by the inherent sensitivity of the cell. To validate model predictions, we performed time-series measurements of nephrin and vimentin localization in micropatterned podocytes and fibroblasts. Podocytes were cultured on the 3-D channel micropatterns, while fibroblasts whose function is dependent on contractile tension^34^ were cultured on rhombus micropatterns with an increasing aspect ratio that would increase intracellular cytoskeletal tension. We evaluated vimentin localization in fibroblasts, which was shown to be a critical modulator of spatial cell biomechanics, changing its cytosolic distribution in response to cellular tension^35^. Vimentin localization in fibroblasts (i.e., area normalized intensity within the tip vs. main body) was compared to nephrin localization in podocytes (i.e., branches vs. cell body). Measurements were carried out in cells treated either with integrin β_3_ blocking antibodies (blocking shape signals) or with 10 μM blebbistatin (blocking tension signals). This design allowed us to observe the dynamics of localization in both podocytes and fibroblasts, which are assumed to be regulated by shape or tension signals. Representative images show the time course of localization of nephrin for podocytes and vimentin for fibroblasts (Fig. 4a). For podocytes treated with blebbistatin, significant nephrin localization into the processes occurred ~ 96 h after plating on biochips; after six days in culture, nephrin remained localized in peripheral processes. In contrast, podocytes treated with integrin β_3_ blocking antibodies showed no nephrin localization. The opposite was true for fibroblasts: cells treated with blebbistatin showed no vimentin localization at the cell tips, while cells treated with integrin β_3_ blocking antibodies showed localization after ~ 2.5 days in culture. These findings show that podocyte phenotype is governed more predominantly by shape signals mediated by β_3_, while tension is a stronger determinant of phenotype in fibroblasts. These findings are further supported by localization specificity assays showing the uniqueness of localization to a given cell and shape (fibroblasts, Supplementary Figs. 3–4; podocytes, Supplementary Fig. 26).

Following localization measurements, we tested whether the dynamics of phenotypic state agreed with the temporal order predicted by the optimal control theory model. For podocytes, we used the solution from $k_{\mathrm{s}}  >k_{\mathrm{f}}$, whereas for fibroblasts we used the solution from $k_{\mathrm{s}} < k_{\mathrm{f}}$. Experimental results and theoretical predictions were compared using non-linear least-square fitting (Supplementary Note 3 for complete fitting details). Dynamics of both cell types were in agreement with the theoretical solution (Fig. 4b). For podocytes, shape signal rose logarithmically while the tension signal showed exponential dependence at a later stage (*t* ≅ days). The trend was reversed for fibroblasts, where tension rapidly overcame shape *t* ≅ 0.42days). Plotting the localization ratio between the shape and the tension signals (Fig. 4b, lower panel) reveals how well the predicted transition times (marked by black dashed lines) fit the experimental dynamics. In agreement with computational predictions, podocytes demonstrate a shape-tension transition in five days whereas fibroblasts switch at six hours, showing that localization phenotype can be optimized to achieve maximal intensity in minimal time through the two different types of signals. Experiments with integrin β_3_ blocking and blebbistatin reveal that cells respond initially to shape signals followed by tension-driven signaling. Hence, we can conclude that the mechanism of phenotypic maturation in podocytes and fibroblasts can be formalized as a minimal time problem under the framework of optimal control theory.

### Shape controls phenotypic maturation of smooth muscle cells

**Fig. 5:**
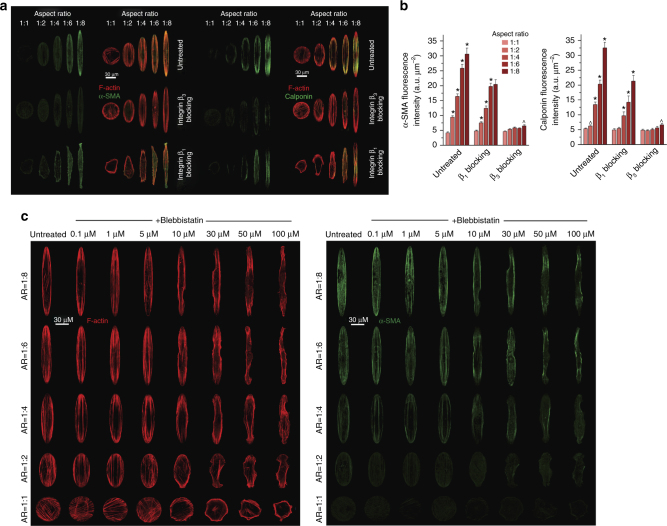
Shape signals control maturation of vascular smooth muscle cells. **a** Representative images of SMCs plated on ellipsoid micropatterns and stained either for (left) α-SMA (green) and F-actin (red), or (right) calponin (green) and F-actin (red). In untreated SMCs, both α-SMA and calponin showed increased expression with increasing aspect ratio that was colocalized with actin stress fibers, which is a hallmark of contractile SMC maturation. When integrin β_3_ activation was blocked, this phenotypic feature was abolished. Treated cells with β_1_ blocking antibodies had no effect on the shape-driven phenotype even though the cells failed to comply with the ellipsoid micropatterns. **b** Quantitative analysis of α-SMA and calponin in SMCs plated on ellipsoid patterns with or without integrin β_1_ or β_3_ blocking antibodies. Values are given as mean ± SEM; *n* = 80, chosen randomly from eight different slides cultured independently (^*p* < 0.05, *p < 0.01 vs. previous ratio; one-way ANOVA comparisons independent for each condition). **c** SMCs were treated with varying concentrations of blebbistatin from 0.1 to 100 μM for 12 h before fixation and stained for F-actin (red) and α-SMA (green). Both stress fiber integrity and compliance of the cells with the micropatterns decrease with increasing blebbistatin concentration; however, α-SMA expression shows little change with the increasing blebbistatin concentration

To assess the generalizability of our hypothesis, we tested whether shape signals through integrin β_3_ can regulate phenotypic state in another cell type. We studied vascular smooth muscle cells (SMCs), which retain phenotypic plasticity that can be modulated in response to extracellular signals^36^. We constructed arrays of ellipsoid micropatterns with varying aspect ratios from 1:1 to 1:8 (Supplementary Figs. 27 and 28). To quantify phenotypic maturation, we determined the expression levels and spatial distribution of two SMC-specific contractile proteins: α-SMA and calponin. Intensity levels of both proteins for ellipsoid-micropatterned SMCs were quantified using immunofluorescence. Both markers demonstrated elevated expression and strong colocalization with actin as the aspect ratio increased. The increased expression was found to be independent of stress fibers (Supplementary Fig. 29). Similar to podocytes, we quantified the role of β_1_ and β_3_ in transducing shape signals to induce phenotypic maturation (Fig. 5a). Cells treated with β_1_ blocking antibodies showed less compliance with the micropatterns; however, as with untreated SMCs, both proteins showed localized expression along actin bundles with increasing aspect ratio as well as increased expression (Fig. 5b). These findings indicate that integrin β_1_ activity only minimally affected the expression levels of proteins in micropatterned SMCs; however, it does not affect the maturation response of cells with shape signals or specialized localization of contractile proteins. In contrast, treating SMCs with β_3_ blocking antibodies abolished this phenotype (Fig. 5b). A similar outcome was observed when the cells were treated with ITGB3 siRNA to knockdown integrin β_3_ levels (Supplementary Fig. 30); spreading areas on ellipsoid micropatterns were unaffected by β_3_ knockdown, and the correlation between α-SMA expressions and cell aspect ratio was lost.

Integrin β_3_ was expressed along actin stress fibers, whereas β_1_ was more distributed across the cell with weak actin colocalization (Supplementary Fig. 31). As the aspect ratio increased, β_1_ was localized to the tips, which may indicate mechanosensitive fibrillar adhesion sites (known to be enriched for α_5_β_1_); these sites were also formed on areas exposed to high mechanical stress as shown to occur in sharp membrane protrusions^28^. We further examined the effect of β_1_ and β_3_ on the maturation of FAs. For the untreated cells, as the aspect ratio increased, the FAs were found to be large, well polarized, and oriented along the cell periphery (Supplementary Figs. 32 and 33). Even though cells treated with integrin β_1_ blocking antibodies were less compliant with the micropatterns, mature and large FAs were observed as the aspect ratio increased (Supplementary Fig. 34). While integrin β_3_ blocking antibody had no effect on spreading, FAs were found to be smaller and markedly less polarized. Similar to podocytes, we also preformed direct inhibition of contractility using blebbistatin to reduce intracellular tension and examined the spreading characteristics and α-SMA expression in micropatterned SMCs (Fig. 5c, complete panels in Supplementary Figs. 35 and 36). Similar to podocytes, we found that low myosin activity was sufficient for the mature phenotype in micropatterned SMCs. This was confirmed using both integrin β_1_ blocking antibodies and blebbistatin. These findings show that shape signals represent an independent source of information for SMCs, similar to podocytes.

### Integrin β_3_ acts through ezrin–radixin–moesin family

**Fig. 6:**
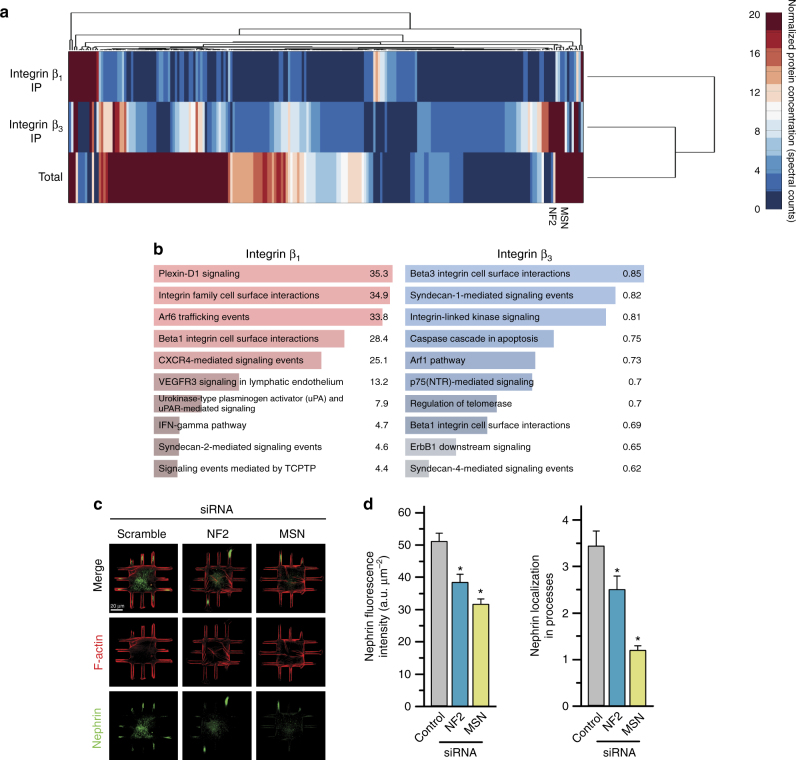
Integrin β_3_ acts through ezrin–radixin–moesin (ERM) family to transduce shape signals. **a** Dendrogram depicting the preferential binding partners of β_1_ and β_3_ as characterized by mass spectrometry. Cell lysates from kidney podocytes were immunoprecipitated (IP) using monoclonal antibodies for the respective integrin isoforms and ran through label-free proteomics and quantified using the spectral counting method. Intensity represents the number of spectra per protein. ERM proteins merlin (NF2) and moesin (MSN) clustered as top differentially bound proteins for integrin β_3_. **b** Enrichment analysis using NCI-Nature ontological database showed “Beta1 integrin cell surface interactions” and “Beta3 integrin cell surface interactions” as the highest over-represented processes for the binding partners of the respective proteins. Color-coded bars represent the shown –log10 *p* value of respective enriched processes. **c** Representative images of podocytes plated on channel micropatterns and transfected with moesin (MSN), merlin (NF2), or scrambled siRNA, and stained for F-actin (red) and nephrin (green). **d** Quantitative analysis of (left) whole-cell nephrin intensity and (right) nephrin localization in processes of podocytes plated on channel micropatterns and transfected with scrambled, NF2, or MSN siRNA. Values given as mean ± SEM; *n* = 80, chosen randomly from four different slides cultured independently (*p < 0.01 vs. untreated control; one-way ANOVA followed by a post hoc Tukey test)

We next aimed to identify the molecular components that are involved in transduction of shape signals through integrin β_3_. We used differential proteomics of β_3_ and β_1_ complexes after immunoprecipitation with isoform selective monoclonal antibodies. For podocytes, these experiments resulted in 219 unique proteins that were differentially localized to the signaling complexes of integrin β_3_ and β_1_ (Fig. 6a); complete results presented in Supplementary Data 1). Enrichment analysis using the NCI-Nature pathway database, which treats integrin isoforms separately, successfully predicted the respective binding partners (Fig. 6b). We also saw that β_3_ precipitates were highly enriched for ezrin–radixin–moesin (ERM) family proteins (merlin, rank 2; moesin, rank 77; and ezrin, rank 156) that are known to be integrin-specific adapter proteins and signaling mediators. We saw that activated ERM proteins (as assessed using p-ERM antibodies) were expressed mostly in peripheral processes of micropatterned podocytes, colocalizing with β_3_ and F-actin (Supplementary Fig. 37), which was abolished when integrin β_3_ was blocked, but was unaffected by integrin β_1_ blocking. The same trend was observed for SMCs (Supplementary Fig. 38).

To test whether ERM is involved in transduction of shape signals, we knocked down ERM proteins merlin and moesin using siRNA, and quantified nephrin expression and localization using immunofluorescence (Fig. 6c). Knockdown of both merlin (NF2) and moesin (MSN) resulted in significant reduction of nephrin expression and localization (Fig. 6d), further supporting that signals through β_3_ and ERM regulate subcellular localization required for physiological function, and they enable us to identify a integrin β_3_-ERM-actin pathway for transducing information flow from cell shape. Our data show that the integrin β_3_-ERM complex provides a link between the membrane and actin cytoskeletal components that is essential for cells to distinguish shape-based and tension-based signaling, which we summarize in a simplified signaling scheme in Supplementary Fig. 39.

### Reaction-diffusion dynamics transduce shape signals

**Fig. 7:**
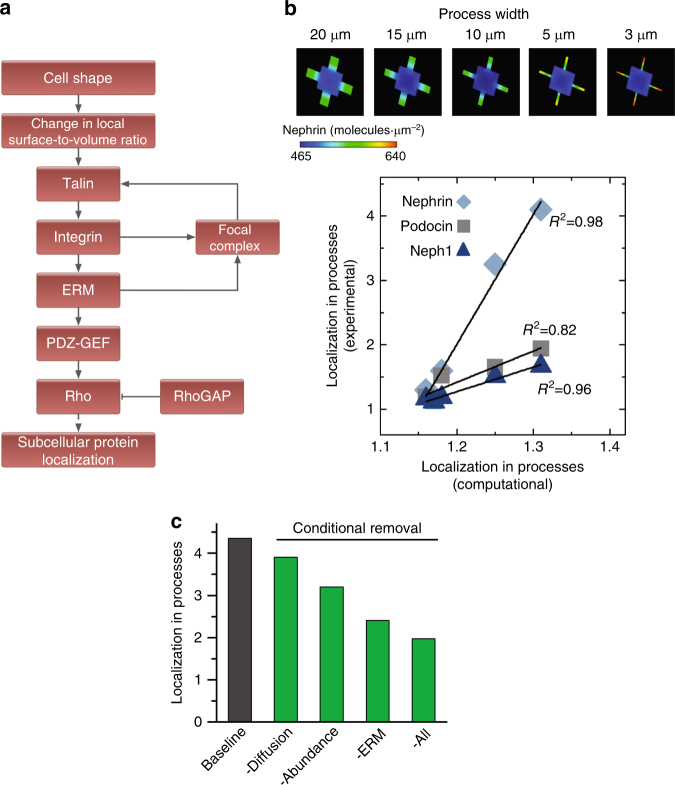
Intracellular reaction-diffusion dynamics enable shape sensing. **a** Schematic of the signaling pathway with an ERM-centered positive feedback loop that induces shape-based subcellular localization of proteins in podocytes. **b** (Top) Simulation results for nephrin localization in 3-D podocytes with peripheral processes of different widths. PDE-based dynamical models confirm the experimentally observed width-dependent localization of nephrin due to spatially heterogeneous focal adhesion assembly. (Bottom) Correlation between experimental localization of slit diaphragm proteins nephrin, podocin, and neph1 and the computational predictions. **c** The effect of experimentally measured molecular attributes of integrin β_3_ on generation and maintenance of localization phenotype was tested by removing individual molecular properties of integrin β_3_ and replacing them with those of integrin β_1_

To understand the physicochemical mechanisms that enable integrin β_3_ to act as an effective shape receptor, we developed a partial differential equation-based dynamical model of integrin activation and the downstream signal flow (Fig. 7a). We used Virtual Cell (VCell)^37^ to build a spatially-specific model of biochemical reactions that can dissect and quantify the respective contributions of individual molecular attributes to relevant phenotypic changes, i.e., protein localization (see Supplementary Note 4 for details).

To parameterize the model with experimentally measured values of the individual integrin subtypes, we quantified the plasma membrane mobility and molecular abundance of β_1_ and β_3_ in SMCs and human podocytes. For the mobility measurements, human podocytes and VSMC were transfected with mEmerald-β_1_ integrin or mEmerald-β_3_ integrin (Supplementary Fig. 40) and plated on glass and fibronectin-coated substrates. Forty-eight hours after transfection, localization and diffusion coefficients of integrin were characterized. For both cell types on glass substrates, mEmerald-β_3_ was enriched in the FAs, while mEmerald-β_1_ was diffuse. In SMCs, fibronectin-coated substrates promoted localization of integrin β_1_ into FAs (Supplementary Fig. 41); however, in podocytes, integrin β_1_ remained uniformly distributed on the membrane even on fibronectin coating with little to no localization to FAs.

To investigate the differences in of integrin β_1_ and β_3_ mobility on plasma membrane, we measured free-diffusion of the two integrin subtypes using fluorescence correlation spectroscopy (FCS)^38,39^. For β_1_, the diffusion coefficients in the presence and absence of fibronectin coating were measured. Since β_3_ localizes to FAs, integrin diffusion inside and outside of FA were measured in the presence and absence of fibronectin coating. For both uncoated and fibronectin-coated substrates, the population of β_3_ outside FAs were significantly more mobile than those inside FAs. We also validated these results using FRAP; mobile fractions of β_3_ outside FAs was higher (MF_β3_ = 0.76 ± 0.08, *n* = 127) compared to inside (MF_β3__−FA_ = 0.34 ± 0.05), in agreement with previous studies demonstrating that integrin exhibits much slower diffusion within FA vs. outside^40^. In contrast, β_1_ was significantly more mobile compared to β_3_ outside of FAs. However, in the presence of fibronectin, both β_1_ and β_3_ outside FAs were less mobile, with greater change in mobility exhibited by integrin β_1_ (61%) compared to β_3_ (47%). The same trend was observed for SMCs on fibronectin (Supplementary Fig. 41). In all, these results suggest that integrin β_3_ displays slower free-diffusion outside of FAs, while integrin β_1_ exhibit higher diffusivity.

Another molecular determinant for formation of spatial information is protein abundance. Proteins that are expressed at lower levels can be more readily segregated into spatial gradients through surface-to-volume effects. In order to measure the levels of integrin β_1_ and β_3_, we used label-free proteomics coupled with the spectral counting method^41^. We found that β_1_ is expressed at five times higher levels than β_3_. To determine if these individual molecular properties controlled shape signaling that modulates cellular functions, we developed a spatial model in VCell and parameterized it using the measured diffusion coefficients, abundance values, and specific kinetic parameters. The goal was to determine the quantitative contribution of different molecular properties of β_3_ with respect to those of β_1_ that would explain how the former acted as an effective shape receptor. Simulations using integrin β_3_ showed increased FA formation and resultant nephrin localization in branches (Supplementary Fig. 42), that also explained the 3-D volume requirement observed for micropatterned podocytes (Fig. 1e) showing that only fully compliant podocytes can achieve amplified concentration of FAs in the branches. We also compared in silico formation of signaling gradients within the processes (Fig. 7b) to the experimentally observed localization of slit diaphragm proteins in podocytes by running virtual geometries of varying widths of peripheral processes. The increased FA signaling in simulations matched the experimentally measured increase of slit diaphragm proteins nephrin, podocin, and neph1 in micropatterned podocytes. Lastly, we systematically replaced the molecular properties of integrin β_3_ with those of β_1_ in the model (i.e., increased diffusion coefficient and abundance, and the inability to bind to ERM proteins). Each of these changes reduced increased localization of FAs into branches, which was almost completely abolished when all three were altered (Fig. 7c). These numerical simulations show that the key molecular properties of integrin β_3_ enable formation and maintenance of spatiotemporal signaling gradients in response to cell shape. These results support our quantitative model for information flow from cell shape, establishing the validity of our general theoretical framework.

## Discussion

Organization of cells within tissues that give rise to organ level physiology is likely to require continuous information flow in both directions: from cell to the tissue microenvironment, and from tissue to the cell. Well-studied modes of information flow within tissues include autocrine signaling that communicates through chemical signals and signaling through cell adhesion molecules that interact with the ECM. These combine information from mechanical forces and chemical interactions; however, they may not fully communicate the exquisite morphological organization of tissues to the single cell. We therefore hypothesized that the physiologically relevant shape of cells within tissues may independently encode information that can be utilized to maintain functional competency. The experiments using 3-D engineered biochips and the computational models in this study show that the shape of cells, separate from other biochemical and biomechanical signals, is an important regulator of cellular phenotype. These findings offer deep insight into the rules governing multiscale organization of tissues for three reasons: (1) cell shape contains information required to maintain the cell in a physiologically relevant phenotype; (2) both shape compliance and signals for physiological functions are dependent on three-dimensional geometric constraints, indicating that cells sense and use packing volume to “know” that they are part of tissue self-organization; and (3) surface-to-volume ratios of physiologically relevant shapes are likely to encode information critical for reaction-diffusion balance required to maintain phenotypic state. Such states are characterized by both expression and subcellular localization of the proteins required for physiologic function. Nature may have favored close to optimal dimensions for enabling information transfer from local surface-to-volume relationships.

We have previously shown that information from cell shape is distinct from the information from the activity level of upstream components^8^. Biochemical signaling pathways at the plasma membrane are modulated by cell shape, thus contributing to decoding shape information^12^. Using microcontact printing, a number of groups showed that mesenchymal stem cells can be shifted towards adipogenic or osteogenic lineages by changing their surface area^3^ or surface-to-volume ratio^5^, suggesting a role for cell shape in early differentiation. However, in these studies on mesenchymal stem cells, the shape signals were decoded by mechanotransduction to activate the Rho pathway, indicating that shape determined the amount of contractile tension being generated along the cytoskeleton. These previous studies show that shape information can modulate mechanical signaling, similar to our findings that shape modulates biochemical signal transduction from hormones and growth factors^12^. In contrast, here, we show that the integrin β_3_-ERM pathway plays an important role in directly transducing signals from cell shape in a tension-independent manner, suggesting that cell shape is a separate signal source. In the native tissue environment, we propose that cells balance signals from biochemical, biomechanical, and shape to control normal physiological function.

## Methods

### Fabrication of 3-D micropatterned biochips

Micropatterning for podocytes, smooth muscle cells, and fibroblast cultures were fabricated by conventional photolithography using SU8 2005 photoresist (MicroChem) onto 22 mm × 22 mm microscope cover glass slides (Corning, Cat: 2725–22). Prior to fabrication, glass coverslips were cleaned by sonication in isopropanol and deionized water for 15 min each and then baked in oven at 110 °C overnight. Photolithography was performed on Süss MicroTec MA6 Mask Aligner (Süss Microtec AG, Germany) using standard vacuum hard-contact mode. UV source was a standard mercury lamp with a power throughput of 8 mJ/cm^2^, and exposure time of 115–125 s with 360 nm long pass filter (Omega Optical, SKU: 2007308) was used in order to ensure straight edges and sidewalls.

Following fabrication, patterned slides were treated with pluronic F-127 (Sigma-Aldrich, Cat: P2443). The natural hydrophobicity of SU8 allowed selective adsorption of pluronic only onto the SU8 and not onto the glass substrate, which ensured that cells could only spread within the patterns. Slides were immersed in 1% (wt/vol) pluronic solution for 3 h. Excess pluronic was washed away using deionized water and PBS. The substrates were then immersed in culture media for 1 h and used immediately thereafter.

### Podocyte cell culture

Human podocytes were a kind gift from Prof. Moin Saleem (Faculty of Medicine and Dentistry, University of Bristol, UK). Methods for podocyte culture and differentiation are based on a previously described protocol^18^. Cells proliferate under permissive conditions (γ-interferon, at 33 °C) but differentiate under nonpermissive conditions (37 °C). Briefly, cells were initially cultured on culture dishes in RPMI 1640 medium (Invitrogen, Cat: 11875119) containing 10% fetal bovine serum (Invitrogen Cat: 26140–079) supplemented with 1% insulin/transferrin/selenium liquid media supplement (Sigma-Aldrich, Cat: I3146) and 100 units/ml penicillin (Invitrogen, Cat: 15140–122). Cultures were incubated in a 37 °C humidified incubator. After two days, cells were collected with trypsin EDTA (Invitrogen, Cat: 25300054) and re-plated on 3-D biochips for continuous culture for five days. Cells were transfected using the Neon Transfection System (Life Technologies). Briefly, cells were harvested and resuspended in electroporation buffer containing 5 µg of DNA, followed by electroporation using a single pulse at 1700 V, 20 ms pulse duration. Cells were then maintained in complete antibiotic-free media until imaging or cell assay is performed.

### Fibroblast cell culture

Neonatal rat cardiac fibroblasts^42^, human dermal fibroblasts^43^, and human foreskin fibroblasts (kind gifts from Prof. Kevin Costa, Icahn School of Medicine at Mount Sinai, New York) cultured in Dulbecco’s modified eagle medium (high Glucose DMEM, Invitrogen, Cat: 11965–118) supplemented with 10% fetal bovine serum, and 100 units/ml penicillin, and maintained in 5% CO_2_ at 37 °C. Cells were harvested after 2 days with trypsin EDTA and re-plated on 3-D biochips for continuous culture for 5 days.

### Human vascular smooth muscle cell culture

HITB5 human vascular smooth muscle cells from Cellutions Biosystems were cultured in M199 medium (25 mM HEPES, Invitrogen, Cat: 12340–030) supplemented with 10% fetal bovine serum, and 100 units/ml penicillin, and maintained in 5% CO_2_ at 37 °C. Cells were harvested with trypsin EDTA and re-plated on SU8 patterned coverslips for continuous culture for 24 h. Coverslips were then resuspended in M199 medium in the absence of 10% FBS for additional two days under the same conditions as mentioned above.

### Rat aortic vascular smooth muscle cell culture

A10 rat thoracic aorta vascular smooth muscle cells from ATCC were cultured in DMEM supplemented with with 10% fetal bovine serum, and 100 units/ml penicillin. Plasmids mEmerald-Integrin-β_1_ N-18 and mEmerald-Integrin-β_3_ N-18 plasmids were gifts from Michael Davidson (Addgene plasmids #54129 and #54130). Cells were transfected using Neon Transfection System (Life Technologies) according to manufacturer’s instructions, with modifications. Briefly, 1 × 10^7^ cells in suspension were transfected with the following settings: 1400 Volts, 10 ms pulse width and pulse number = 2. Plasmid amounts were varied from 2–20 µg. Cells were seeded on glass coverslips and imaged 48 h post-transfection. Cells were imaged in Hank’s Balanced Salt Solution supplemented with 1 mM HEPES and 1 mM Pyruvate.

### COS-7 cell culture

Cells from ATCC were maintained at 37 °C in 5% CO_2_ in DMEM (Life Technologies) supplemented with 10% FBS (Life Technologies) and 1% penicillin-streptomycin (Life Technologies). PMMA-patterned coverslips were incubated with 0.5% Pluronic F127 (Sigma-Aldrich) for 3 h, washed with PBS followed by 0.1 mg/ml poly-L-lysine treatment (Sigma-Aldrich) for 1 h. Coverslips were then washed and cells were plated in DMEM supplemented with 10% FBS. After 4–6 h, cells were serum starved overnight in DMEM supplemented with 0.1% BSA. At the time of plating, patterned cells were transfected with the cAMP biosensor EPAC1 and Lipofectamine 2000 (Life Technologies) in OPTI-MEM following manufacturer’s instructions. After 4–6 h, cells were serum starved with 0.1% BSA DMEM overnight.

### Integrin and myosin inhibition assays

Prior to plating on the patterned surfaces, podocytes/SMCs were suspended in RPMI/M199 media containing either integrin β_3_ blocking antibodies (B3A, EMD Millipore, Cat: MAB2023Z) or integrin β_1_ blocking antibodies (6S6, EMD Millipore, Cat: MAB2253) at a concentration of 1 mg/ml for 1 h at 37 °C. For podocytes, a second and third dose of antibodies at a dilution of 1:100 were added to the culture media two and four days after cells seeding, respectively.

Non-muscle myosin IIa inhibitor blebbistatin (Sigma, Cat: B0560) was dissolved in DMSO to a stock concentration of 5 mg/ml and added to the RPMI culturing media 12 h prior to fixation at a final concentration of 0.1, 1, 5, 10, 30, 50, and 100 μM respectively. For fibroblasts, blebbistatin was added to the DMEM culturing media at a final concentration of 10 μM 12 h prior to fixation. Rho kinase inhibitor Y-27632 (Sigma, Cat: Y0503) was dissolved in DMSO to a stock concentration of 5 mg/ml and added to the RPMI culturing media 6 h prior to fixation at a final concentration of 0.1, 1, 5, 10, 30, 50, and 100 μM, respectively.

### Real-time PCR

Total RNA was extracted using the RNeasy kit (Qiagen, Cat: 74104) per manufacturer’s instructions. First strand cDNA was prepared from total RNA (~ 1.0 μg per reaction) using the SuperScript III first strand synthesis kit (Invitrogen, Cat: 1461361), and cDNA was amplified in duplicates using Quantitech SYBR Green kit (Qiagen, Cat: 204143) on an AB PRISM 7500 (Applied Biosystems) real-time PCR machine. Pre-designed Quantitech primer sets were obtained from Qiagen for Homo sapiens NPHS2 (podocin), WT1, DDN (dendrin), NPHS1 (nephrin), MAGI2 (membrane associated guanylate kinase, WW and PDZ domain containing 2), CDH3 (p-cadherin), CD2AP, KIRREL (Neph1), PARD3B (Par-3 family cell polarity regulator), ACTN4 (α-actinin-4), SYNPO (synaptopodin) and GAPDH. AB7500 analysis software was used to determine crossing points using the second derivative method. Changes in the expression levels of eleven differentiation genes were normalized to the housekeeping gene (GAPDH) and presented as fold increases compared with RNA isolated cells cultured on unpatterned glass using the 2^−ΔΔCT^ method^44^.

### Immunostaining

Cells were fixed with 4% paraformaldehyde in PBS for 20 min and permeabilized with 0.5% Triton-X for 10 min at room temperature. After washing with PBS and blocking in 4% bovine serum albumin, cells were incubated overnight independently with a primary antibody at a given dilution as summarized in Supplementary Table 4. Primary antibody incubation was followed by fluorescently tagged anti-rabbit (Alexa Fluor 488, Cell Signaling, Cat: 4412 s) or anti-mouse (Alexa Fluor 647, Cell Signaling, Cat: 4410 s) secondary antibodies. Phalloidin (Alexa Fluor 568, Invitrogen, Cat: A12380) and Hoechst 33342 (Invitrogen, Cat: H3570) were used to stain F-actin and the nuclei, respectively. Images were acquired by Zeiss LSM 700 laser scanning microscope using ZEN Lite 2011 software. Images were recorded under the same conditions for each marker (i.e. voltage gain, pinhole, digital offset). All spatial fluorescence intensity measurements were carried out in a pairwise fashion where patterned and unpatterned cells were imaged on the same coverslip under identical microscope settings. Signal-to-noise ratios for all antibodies were first tested by imaging with and without primary antibody on unpatterned glass surfaces for all cell types. All representative images in the manuscript are composites whereby individual images were assembled together to highlight the phenotype and to show progressive variations due to cell shape.

### Morphometric analyses

Analysis of patterned cells was carried out by a random selection of single cells with pattern compliance greater than 90%. The compliance *C* is defined as the ratio of the paterned glass area *A*_g_ over the sum of both glass and SU8 barrier (*A*_b_) areas covered by the cell, given by:

M.1 $$C = \frac{{A_{\mathrm{g}}}}{{A_{\mathrm{g}} + A_{\mathrm{b}}}}$$

For immunostaining quantification, cells were chosen randomly at 63x magnification in order to capture a representative population without biased phenomenology. Statistics were performed from images taken at 20x magnification with a total of 30–50 different areas being scanned per slide. The average fluorescence intensities were calculated as arbitrary units per square micrometer (a.u./μm^2^). The localization ratio was used only for podocytes and fibroblasts. For podocytes, the localization ratio was used for cells plated on the channel patterns and defined as the intensity per unit area in the channels divided by the intensity in the main box area per unit area (branches vs. cell body). For fibroblasts, the localization ratio was used for cells plated on the rhombus patterns and defined as the intensity per unit area in the tip (effective area = 150 μm^2^) divided by the intensity of the main body per unit area (effective area = 900 μm^2^). The data was analyzed for variance using one-way ANOVA, *p* < 0.05 was considered as statistically significant. Protein localization was defined for cells on SU8 patterns and represented the ratio of fluorescence intensities between two regions of interests (ROIs) randomly located within the tip/boundary of the cell and the centroid.

Morphological characteristics, including average spreading area and aspect ratio (AR) were measured independently for patterned and unpatterned podocytes. Spreading area was calculated based on the contour of the cell, while the aspect ratio was based on a fitted ellipse representing the ratio of the long axis to the short axis. The spreading area and aspect ratio were calculated using ImageJ based on F-actin stained cells. Similarly, *D* analysis was carried out using p-FAK immunostaining, where images were thresholded and segmented using Matlab, and later quantified using ImageJ.

### Electron microscopy

Samples were fixed in 2.5% glutaraldehyde in 200 mM sodium cacodylate buffer overnight, washed with cacodylate buffer (pH 7.4) and osmicated with 1% osmium tetroxide. For scanning electron microscopy (SEM), cultured human podocytes on coverslips and 200 μm-thick rat cortical kidney slices were dehydrated through serial ethanol and critical point dried with liquid carbon dioxide. They were then sputter coated with gold palladium twice, and imaged on a Hitachi S-4500 SEM. For transmission electron microscopy, human cortical kidney samples (fixed in 2.5% glutaraldehyde) or puromycin-induced nephropathy rat cortical kidney samples (fixed in 2.5% glutaraldehyde) were dehydrated in steps with ethanol and propylene oxide washes and embedded in Embed 812. One-micrometer plastic sections were cut and stained with methylene blue plus azure II to localize representative structures. Samples were then cut into 60 nm ultrathin sections, stained with uranyl acetate-lead citrate and imaged on a Hitachi H-7650 TEM.

### FRAP measurements

FRAP measurements were performed at 25 °C on Zeiss LSM 780 inverted microscope equipped with 488 nm argon ion laser and gallium arsenide phosphide photon detectors and a 40x Plan-Apochromat oil objective (NA = 1.4). A circular region of interest (radius = 2 μm) at the bottom membrane of the cells was selected for bleaching, either centered on a FA, or on an area that is devoid of FAs. The region of interest was illuminated with high intensity (100% transmittivity) 488 nm argon ion laser for 500 ms and observed for 120 s using low intensity (2% transmittivity) laser power. Under these bleaching conditions, the fluorescence intensity in the region of interest immediately after photobleaching was 40% of the intensity of the pre-bleach fluorescence. Correction for photobleaching was performed by fitting an exponential decay equation to a non-bleached region interest over time. FRAP curves were normalized by the average intensities of the prebleached region (*I*_max_) and average intensity of the region of interest immediately after bleaching (*I*_min_). The half-maximal recovery time (*t*_1/2_) and the mobile fraction were calculated from the normalized FRAP curves, by fitting a one component exponential function to the time dependent fluorescence recovery.

### FCS measurements

FCS measurements on cells expressing mEmerald-integrin β_3_ N-18 or mEmerald-integrin β1 N-18 were performed on Zeiss LSM 780 system as previously described^38^ using a 40x C-Apochromat W (NA 1.2) water immersion objective. mEmerald-tagged proteins were excited with 488-nm argon ion laser line (0.5% transmittivity). Fluorescence emission was collected on gallium arsenide phosphide detector collected through the 488 nm main beam splitter collecting emission light from 498–596 nm. The focal volume was calibrated with 10 nM Rhodamine 6 G solution (*D* = 2.8 × 10^−6^ cm^2^/s). For each cell, a pre-bleaching step was performed using 100% laser power for 5 s and then subsequent illumination with 0.5% transmittivity. FCS measurements were recorded at 25 °C in HBSS supplemented with HEPES and Pyruvate for 50 s (5 consecutive 10 s intervals). FCS data were fitted to a two-dimensional diffusion model shown by the following equation:

M.2 $$G\left( \tau \right) = 1 + \frac{1}{N}\left( {(1 - Y)\left( {1 + \frac{\tau }{{\tau _{D1}}}} \right)^{ - 1}\left( {1 + \frac{\tau }{{S^2\tau _{D1}}}} \right)^{ - \frac{1}{2}} + Y\left( {1 + \frac{\tau }{{\tau _{D2}}}} \right)^{ - 1}\left( {1 + \frac{\tau }{{S^2\tau _{D2}}}} \right)^{ - \frac{1}{2}}}\right)$$

where *N* is number of fluorescent particles in the confocal volume, *S* is structural parameter which defines the aspect ratio of the confocal volume, *τ*_*D*1_ is the diffusion time of the faster component, 1−*Y* is the fraction of particles with diffusion time *τ*_*D*1_, *τ*_*D*2_ is the diffusion time of the slower component, and *Y* the fraction of particles with diffusion time *τ*_*D*2_. A fitting algorithm using the iterative least-square method was performed in MATLAB environment. Structural parameter was set to 100 (quasi-infinite) for two-dimensional diffusion. Diffusion coefficients were calculated from the diffusion time, *τ*_*D*_, using the Einstein relation for diffusion: $D = \omega ^2/4\tau _{D2}$, where ω is the radius of the observation volume.

### FRET imaging of COS-7 cells

Cells were imaged in a Zeiss LSM meta-510 inverted confocal microscope. Coverslips containing the patterned cells were mounted in a temperature controlled imaging chamber (Warner) with recirculation of HEPES buffered imaging media [1X HBSS (Life Technologies) supplemented with 10 mM glucose and 25 mM HEPES (Life Technologies), pH 7.4]. After a stable baseline, cells were stimulated with 0.1 μM isoproterenol (Sigma-Aldrich). 512 × 512 images were acquired using a 63X objective. Fluorophores were excited with an argon laser at 458 nm (ECFP and FRET), or at 514 nm (EYFP). FRET was calculated using the net FRET (nF) method in Volocity (Improvision/Perkin Elmer). Color-coded nF was plotted as a function of pixel or plotted as a function of time.

### FRET analysis of vinculin tension biosensor

Analysis of FRET was performed as described^45^. Vinculin tension bioensor plasmid was a kind gift from Martin Schwartz (Addgene plasmid # 26019). Human podocytes and A10 vascular SMCs were transfected with vinculin tension biosensor using Neon Transfection System (Life Technologies) following manufacturer’s instructions. Twenty-four hours after transfection, cells were treated with 1, 3, 10, 30 and 100 µM of blebbistatin for two hours. Media was then replaced with Hank’s Balanced Salt Solution (w/o phenol), supplemented with pyruvate (1 mM) and penicillin-streptomycin immediately before imaging. Live-cell FRET imaging was performed using Zeiss LSM 880 confocal microscope equipped with 1.4 NA water objective and with humidity and CO_2_ control. eCFP was excited with 458 nm emission line using an Argon ion laser and eYFP was excited with 515 nm emission line. eCFP, FRET and eYFP channels were simultaneously imaged using an internal GaAsp detector collecting the emission from a range of wavelengths appropriate for each channel: For eCFP channel: 463 nm – 520 nm, FRET channel: 520 nm – 620 nm and eYFP channel: 520 nm – 620 nm. Intensity based ratiometric FRET indices were obtained on a per-cell and per-focal adhesion basis using custom-written scripts in ImageJ and MATLAB. Stepwise, images were sorted into donor, FRET and acceptor channels. Each set of images from different channels was registered and background corrected. To obtain FA masks, CLAHE and Laplacian of Gaussian (LoG) were performed on the eYFP channel, followed by applying size and intensity filter on the resulting particles. Since vinculin tension biosensor is a single-chain construct, FRET was calculated by dividing the FRET channel (donor excitation with acceptor emission) by the sum of the CFP channel (donor excitation with donor emission) and FRET channel (donor excitation with acceptor emission) (Ia/(Id + Ia)). FRET indices display an increasing trend with increasing concentration of blebbistatin. FRET indices were then normalized by dividing the FRET indices by the values of the basal (0 µM) concentration.

### Proteomics

Cells were collected, lysed with immunoprecipitation (IP) lysis buffer (25 mM Tris, 150 mM NaCl, 1 mM EDTA, 1% Triton-X), and immunoprecipitated using DynaBeads (Life Technologies) coated for 24 h with monoclonal antibodies against integrin β_1_ or β_3_, according to manufacturer’s protocol. Lysates were immunoprecipiated for 16 h with (or without) antibodies, washed (using 1 M NaCl + 0.1% Tween 20 wash buffer) and beads were pulled down using the DynaBead magnet system, according to manufacturer’s protocol. Precipitates for anti-β_1_ integrin, anti-β_3_ integrin, negative control (beads alone with no antibody) and total lysates were run through tandem LC-MS/MS and spectral counts were quantified using the Scaffold Suite (Proteome) as previously described^46^. A protein was selected as a differential binder of a given integrin isoform if its IP spectral counts were greater than twice the IP counts of the other isoform.

### ITGB3 knockdown

Small interfering RNA for ITGB3 (Catalog Number: L-004124–00–0005) and siCONTROL non-targetting siRNA pool (Cat. number D-001810–10) were purchased from Dharmacon. HITB5 cells were transfected according to manufacturer’s instructions. Briefly, HITB5 cells were counted (10^5^ cells) and plated onto microfabricated surfaces or to 6-well dishes. Twenty-four hours later, growth medium was aspirated and replaced with Accell delivery medium (serum-free) containing a final concentration of 1 µM ITGB3 siRNA or 1 μM Non-targetting siRNA. 48 h later, media was aspirated and replaced with M199 media supplemented with 0.5% FBS. Cells were fixed and stained 96 h after siRNA treatment. For cells in 6-well dishes, cells were lysed and assayed for protein knockdown using Western blotting.

### Statistical analyses

All experiments were repeated at least three times; means were obtained from three or more independent experiments. The data were presented as mean ± standard error of the mean. Statistical differences were determined using unpaired *t* test or one-way ANOVA followed by a post hoc Tukey test, where appropriate. Significance was defined at a *p* < 0.05.

### Data availability

Data supporting the findings of this study are available within the paper and its supplementary information. The raw mass spectrometry proteomics data have been deposited to the ProteomeXchange Consortium via the PRIDE partner repository with the dataset identifier PXD008150 and 10.6019/PXD008150.

## Electronic supplementary material

Supplementary Information

Description of Additional Supplementary Files

Supplementary Data 1
