# Supplementary material for: Cell shape information is transduced through tension-independent mechanisms
Source: Nat Commun. 2017 Dec 15;8:2145. doi: 10.1038/s41467-017-02218-4 (PMC5732205; doi:10.1038/s41467-017-02218-4)
Supplement: Supplementary file 1 — Supplementary Information [file 41467_2017_2218_MOESM1_ESM.pdf]

Supplementary Information Table of Contents

SUPPLEMENTARY FIGURES 2
SUPPLEMENTARY TABLES 44
SUPPLEMENTARY NOTE 1: REACTION-DIFFUSION MODEL 48
1.1 Derivation of analytical and computational framework 48
1.2 Graphical solutions and biophysical interpretations 49
SUPPLEMENTARY NOTE 2: CELLULAR ACTIVATION MODEL 53
2.1 Derivation of analytical framework 53
2.2 Shape-driven model 54
2.2.1 Surface-to-volume effect 54
2.2.2 Analytical definition of shape-dependent rate constant 56
2.3 Tension-driven model 58
2.3.1 Introduction and definitions 58
2.3.2 Biophysical concept 59
2.3.3 Cellular measures 60
2.3.4 Analytical and computational methodology 61
2.4 Catch-bond model 64
SUPPLEMENTARY NOTE 3: CONTROL THEORY MODEL 67
3.1 Derivation of analytical framework 67
3.2 Estimation of control parameters: nonlinear least squares fit 71
3.3 Levenberg-Marquardt Algorithm 72
SUPPLEMENTARY NOTE 4: SIGNALING MODEL 76
SUPPLEMENTARY REFERENCES 79

Supplementary Figures

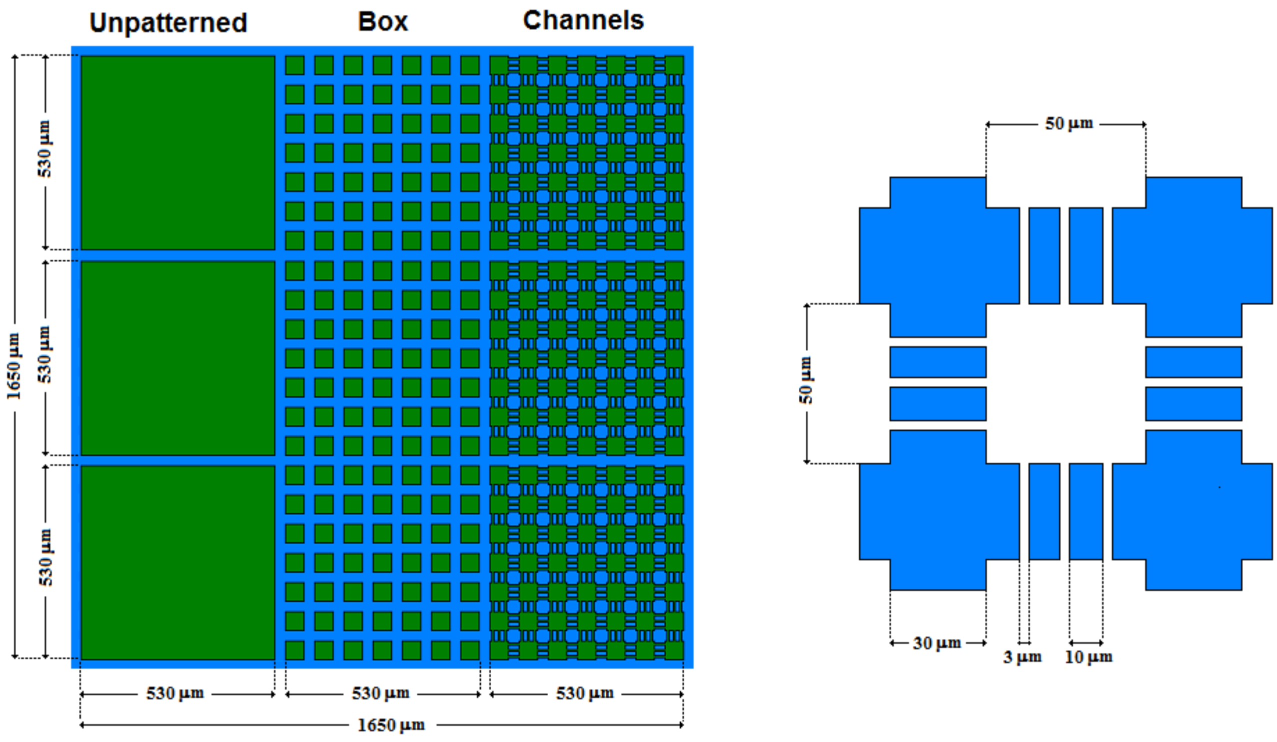

Supplementary Figure 1: Architecture of fabricated biochip used for podocyte culturing. **(Left)** The 3 x 3 heterogeneous culture surface where unpatterned, box micropatterned, and channel micropatterned cells can be co-cultured. **(Right)** Geometrical characteristics of a single channel micropattern.

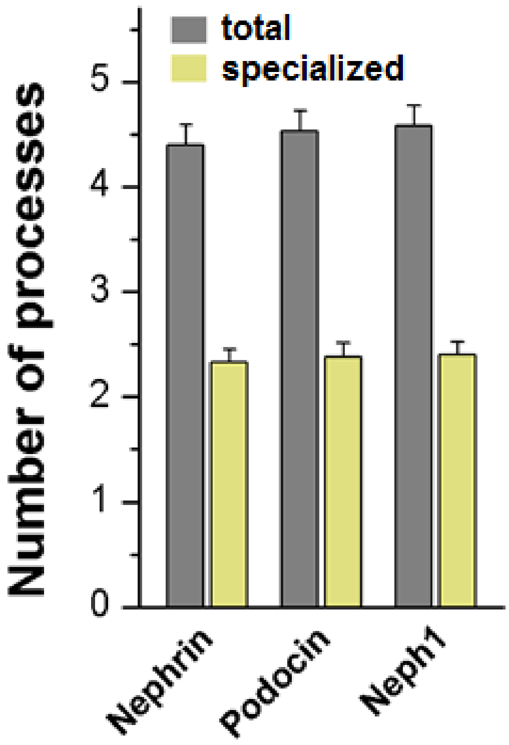

Supplementary Figure 2: Quantitative analysis of specialization of processes in podocytes cultured on channel micropatterns independently using nephrin, podocin, and neph1. We defined a "specialized process" as any peripheral branch that has increased localization levels of slit diaphragm proteins. The total number of processes and the percentage of processes with increased amount of slit diaphragm proteins were similar for all the markers. Values given as mean ± SEM; n = 80, chosen randomly from eight different slides cultured independently at different times.

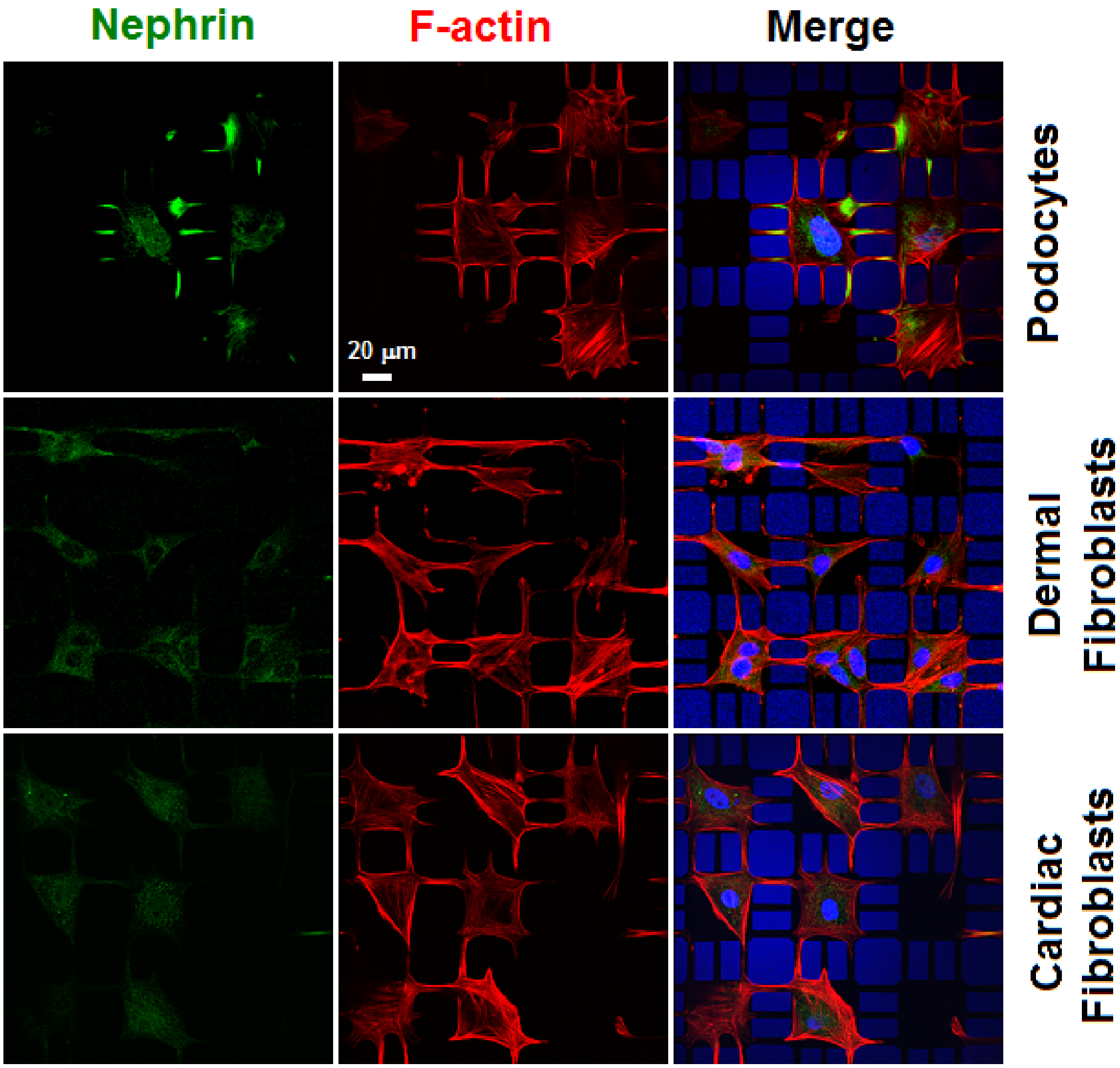

Supplementary Figure 3: Selective and cell type specific localization of nephrin and F-actin in podocyte processes. Representative immunofluorescence images of human podocytes, human dermal fibroblasts, and neonatal rat cardiac fibroblasts cultured on channel micropatterns and stained for nephrin (green), F-actin (red) and nuclei (blue). Nephrin and F-actin enrichment in processes can be observed in podocytes but not in cardiac or dermal fibroblasts.

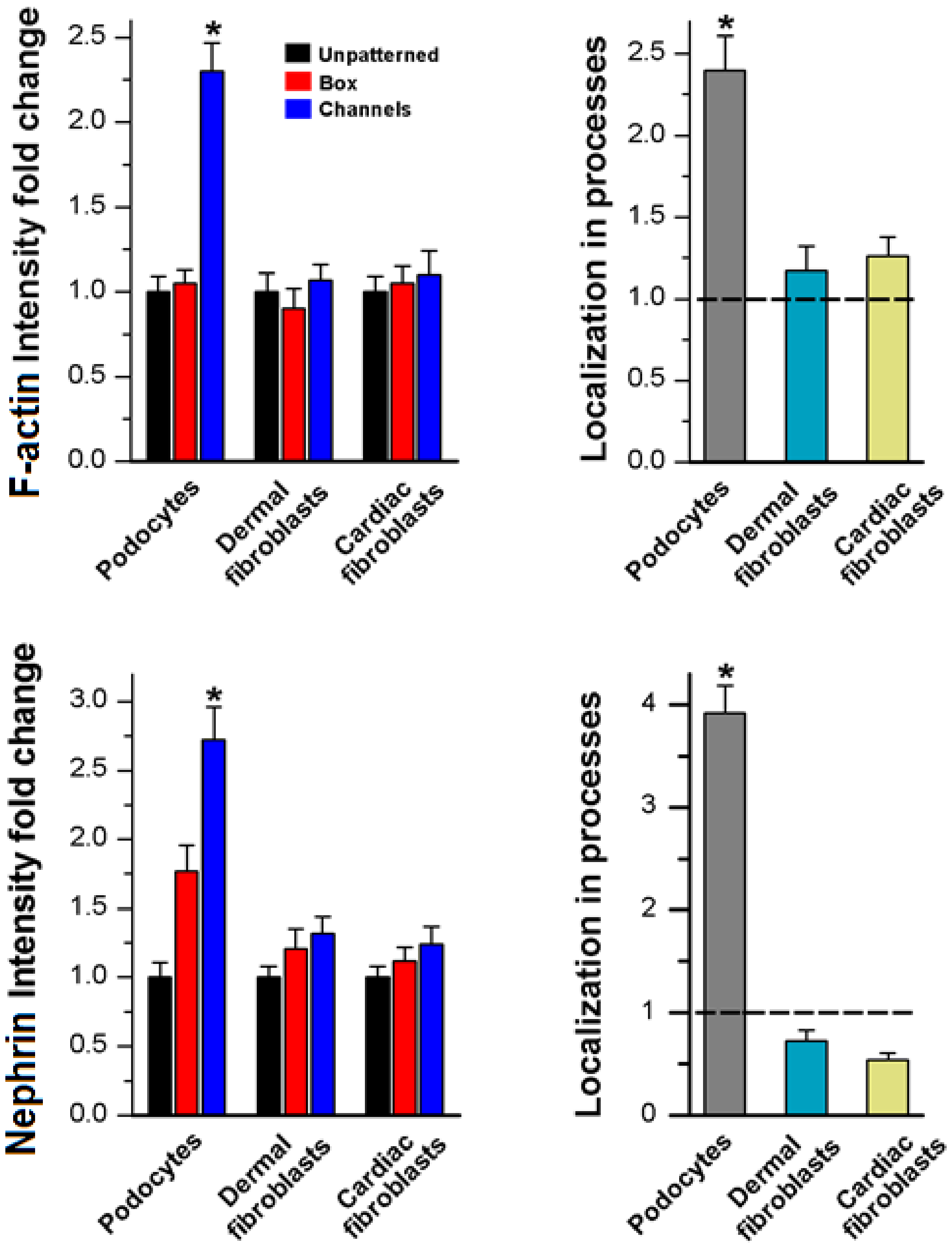

Supplementary Figure 4: Quantitative analysis of nephrin and F-actin in podocytes, cardiac fibroblasts and dermal fibroblasts cultured on unpatterned glass, box, and channel micropatterns. Fibroblasts express lower levels of nephrin and F-actin when plated on channels and do not exhibit localization in peripheral processes (n = 80 cells each, chosen randomly from eight different slides cultured independently; ***p < 0.01; one-way ANOVA with post-hoc Tukey).

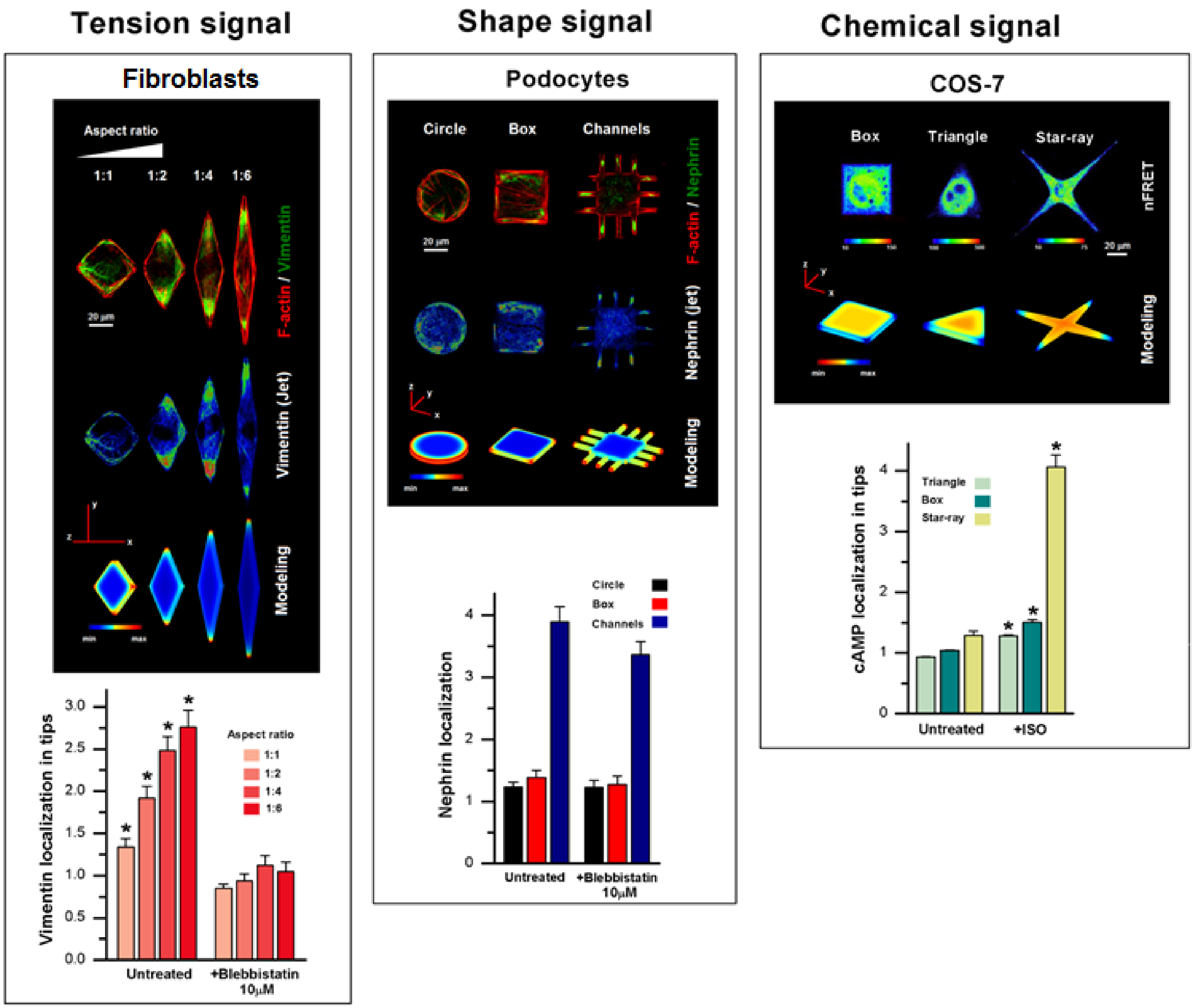

Supplementary Figure 5: 3-D micropatterned cells showing different localization patterns of proteins in fibroblasts, podocytes and COS-7 cells, driven by contractile tension, shape, and chemical signals, respectively. Fibroblasts on rhombic patterns stained for vimentin (green) and F-actin (red) show increased vimentin localization in cell tips as the cell aspect ratio increased. Podocytes on square, circle, or channel micropatterns stained for nephrin (green) and F-actin (red) show nephrin localization within the channels; in square/circle-shaped podocytes, the localization was mainly on the cell periphery. Upon stimulation with isoproterenol (ISO), COS-7 cells on square, triangle, or star shapes showed the presence of cAMP microdomains in sharp edges. Reaction-diffusion simulations (3-D slab models on middle row) show close agreement with the experimental observations. Quantification of protein localization in different patterns (histograms on the bottom row) show that vimentin localization increases with aspect ratio, which is abolished upon treatment with 10 µM blebbistatin. Nephrin shows high localization within channels which is not affected by blebbistatin treatment. cAMP localization in COS-7 cells shows shape-dependent changes only when stimulated with isoproterenol. All values given as mean ± SEM; n = 40, chosen randomly from 4 different slides, cultured independently (* $p < 0.01$ vs. untreated cells; one-way ANOVA with post-hoc Tukey).

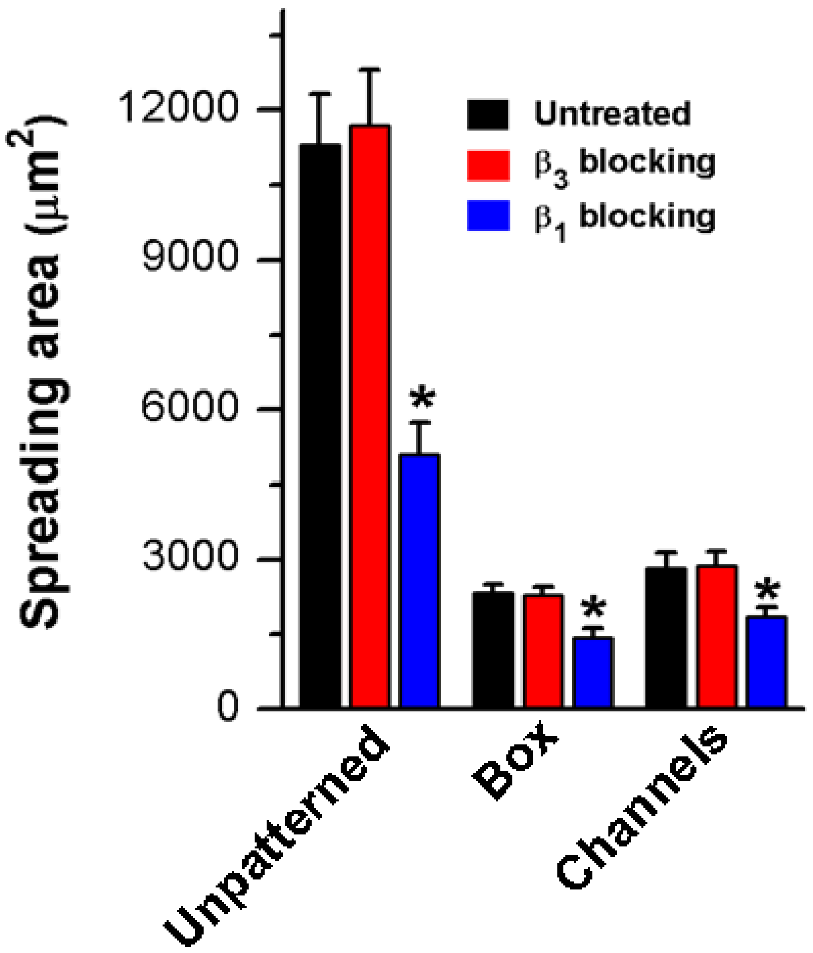

Supplementary Figure 6: Spreading area of unpatterned and micropatterned podocytes with and without blocking of integrin β_3 or β_1 prior to plating. Values given as mean ± SEM; n = 80, chosen randomly from eight different slides cultured independently (*****p < 0.01 vs. untreated; one-way ANOVA with post-hoc Tukey).

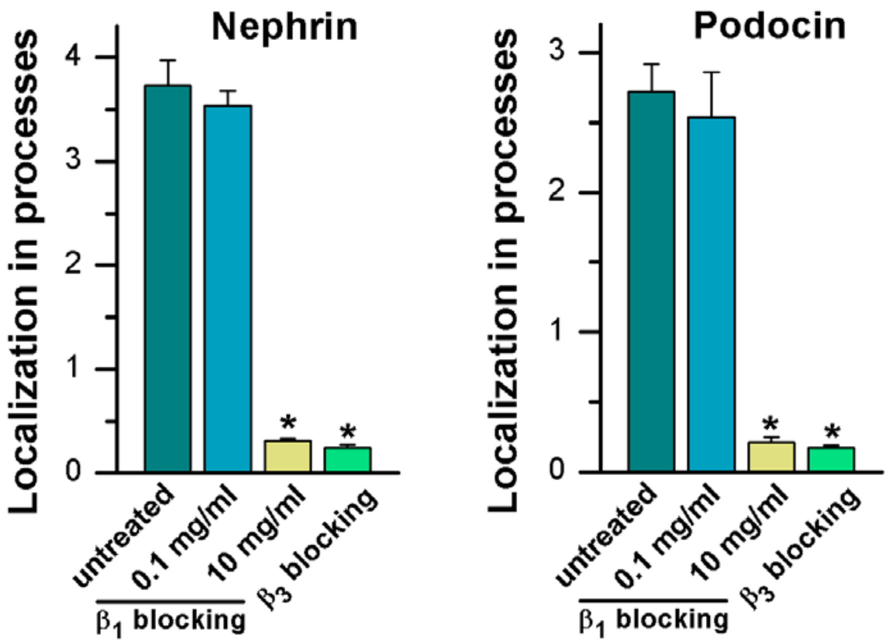

Supplementary Figure 7: Quantitative analysis of nephrin and podocin localization in processes in podocytes plated on channel micropatterns and treated with either integrin β_1 or β_3 blocking antibodies. For untreated cells and for cells treated with a low dose of integrin β_1 antibody that prevents signaling but not spreading, both markers show high localization ratio. Cells treated with high dose of integrin β_1 antibody show little localization due to limited spreading and loss of processes. Cells treated with the integrin β_3 antibody show little or no localization. Values given as mean ± SEM; n = 80, chosen randomly from 8 different slides cultured independently (*****$p < 0.01$ vs. previous concentration; one-way ANOVA with post-hoc Tukey).

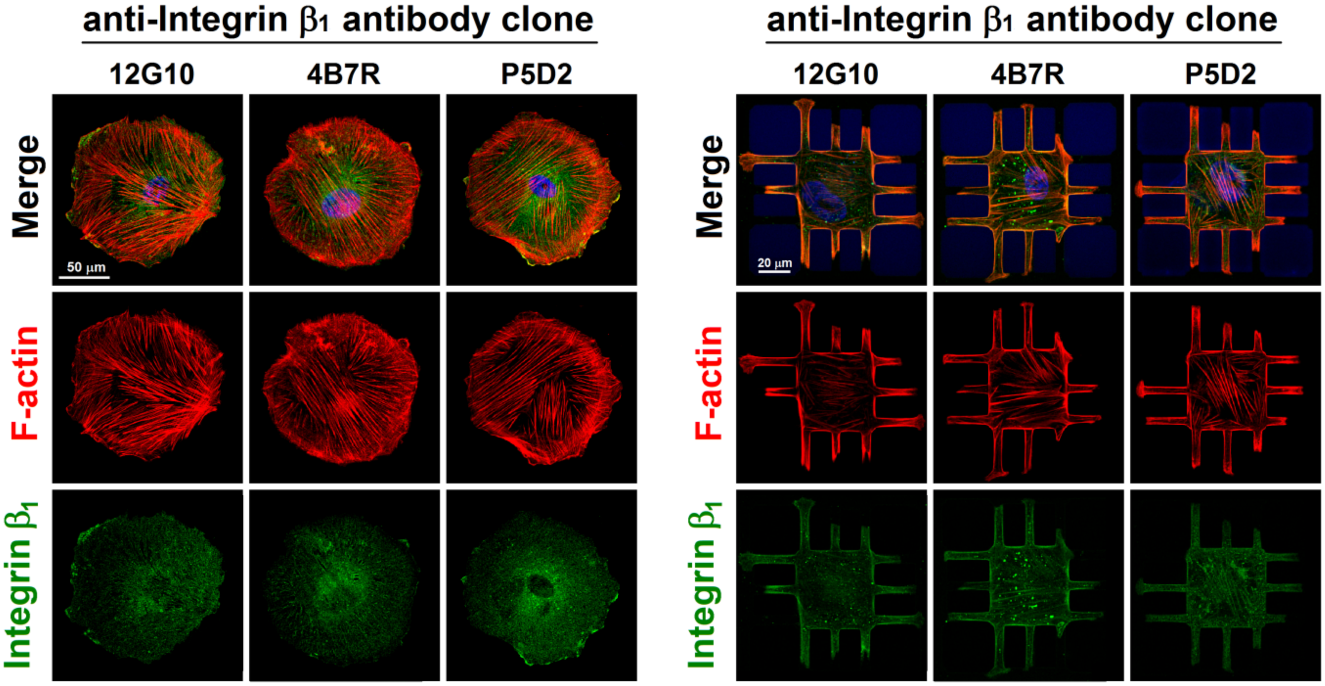

Supplementary Figure 8: Validation of integrin β_1 antibodies. Representative images of integrin β_1 (green) and F-actin (red) staining in podocytes plated on unpatterned (left) and micropatterned surfaces (right). Staining of integrin β_1 was performed using three different clones, 12G10, 4B7R, and P5D2. For unpatterned cells, integrin β_1 was distributed throughout the cell with localized clusters along the membrane edges. On patterned cells, integrin β_1 was expressed throughout the cell and along the cell edges. The staining patterns were found to be similar for all tested clones.

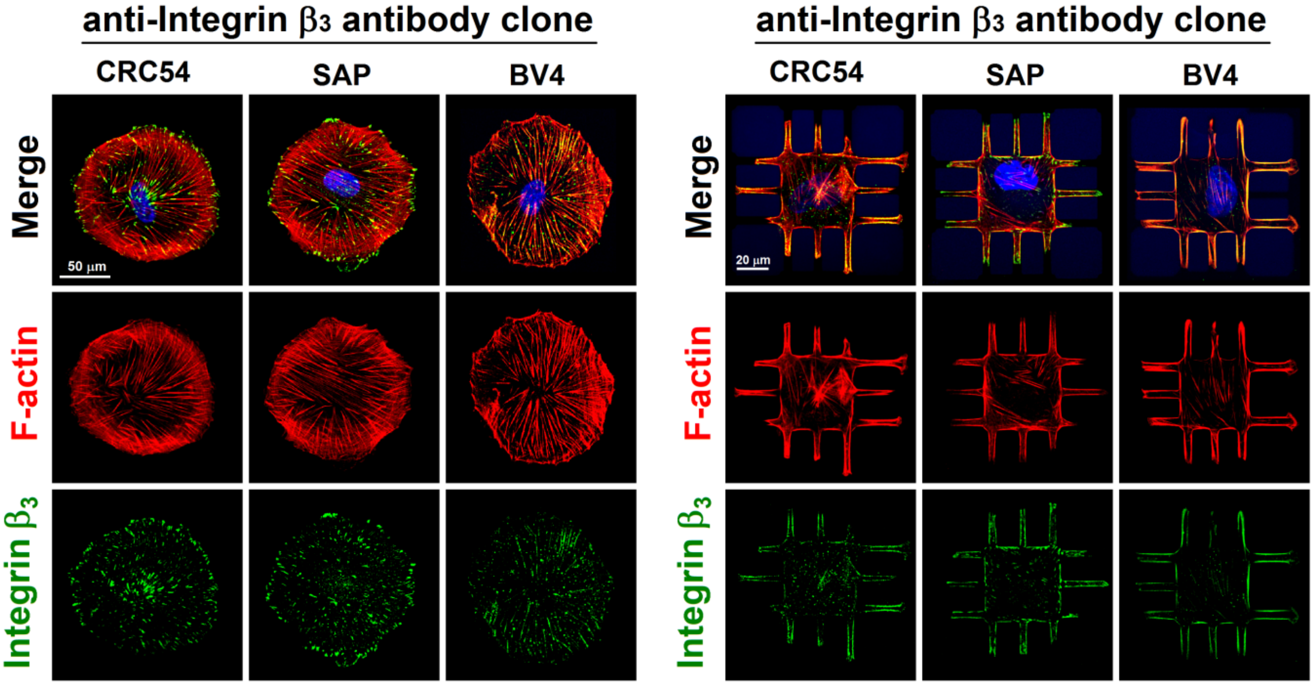

Supplementary Figure 9: Validation of integrin β_3 antibodies. Representative images of integrin β_3 (green) and F-actin (red) in podocytes plated on unpatterned (left) and micropatterned surfaces (right). Staining of integrin β_3 was performed using three different clones, CRC54, SAP, and BV4. For unpatterned cells, integrin β_3 showed strong clustering along actin bundles. On patterned cells, β_3 showed strong colocalization with actin and was mainly expressed along the cell edges. The staining patterns were found to be similar for all tested clones.

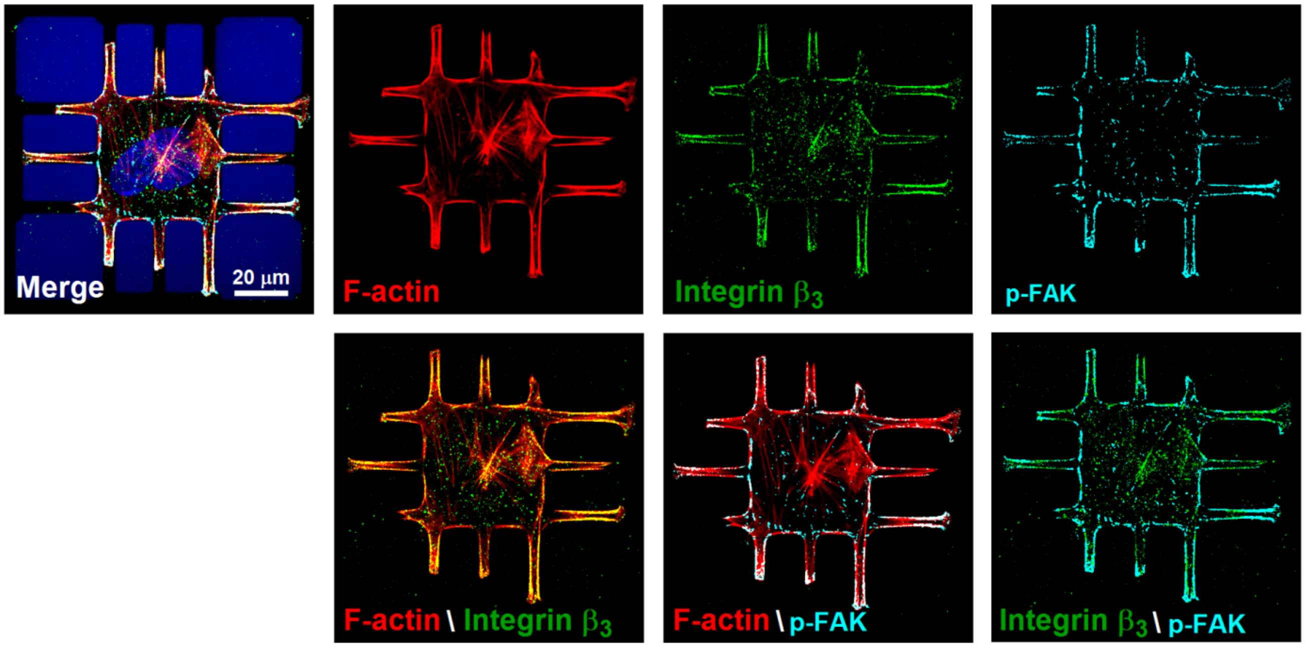

Supplementary Figure 10: Staining of activated FAK in micropatterned podocytes. Representative images of p-FAK (cyan), integrin β_3 (green), and F-actin (red) staining in podocytes plated on micropatterned surfaces. Integrin β_3 was expressed along actin stress fibers and showed clear colocalization with focal adhesions.

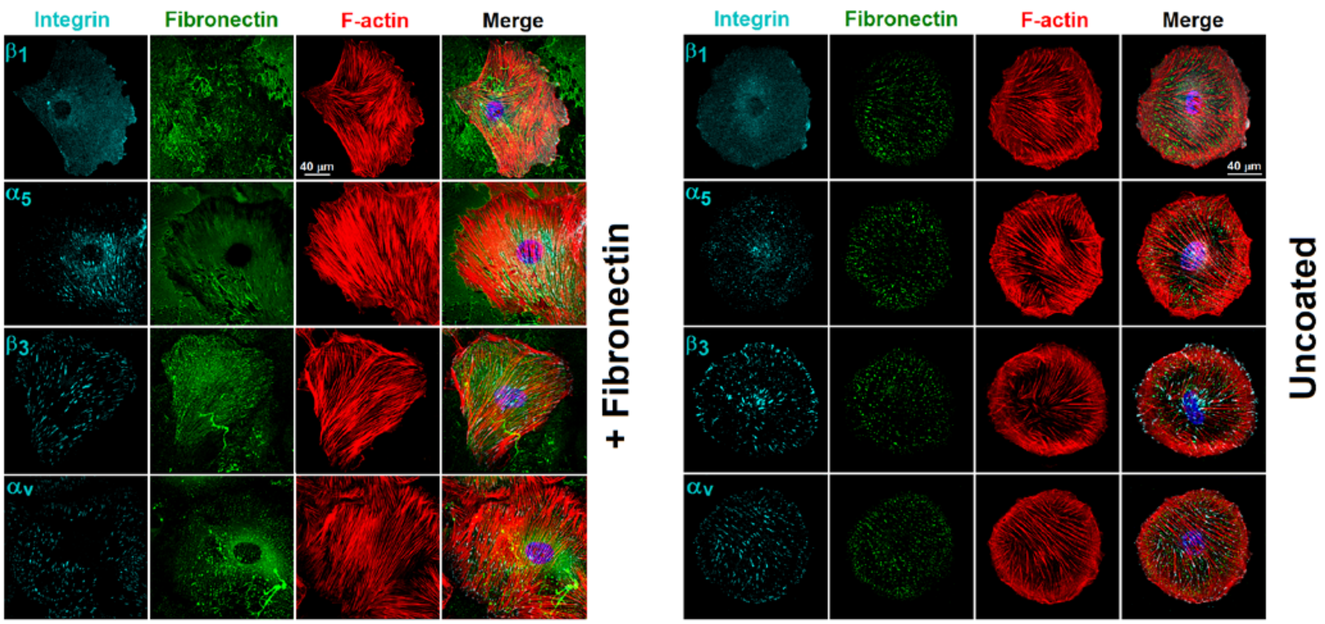

Supplementary Figure 11: Integrin staining in unpatterned podocytes. Representative images of integrin β_1, α_5, β_3, α_v (cyan), F-actin (red), and fibronectin (green) staining in podocytes plated on unpatterned surfaces with and without prior fibronectin coating. Cells plated on surfaces coated with fibronectin exhibit significantly higher spreading area compared to those plated on uncoated surfaces. Fibronectin promoted clustering of integrin $\alpha_v\beta_3$ along F-actin fibers.

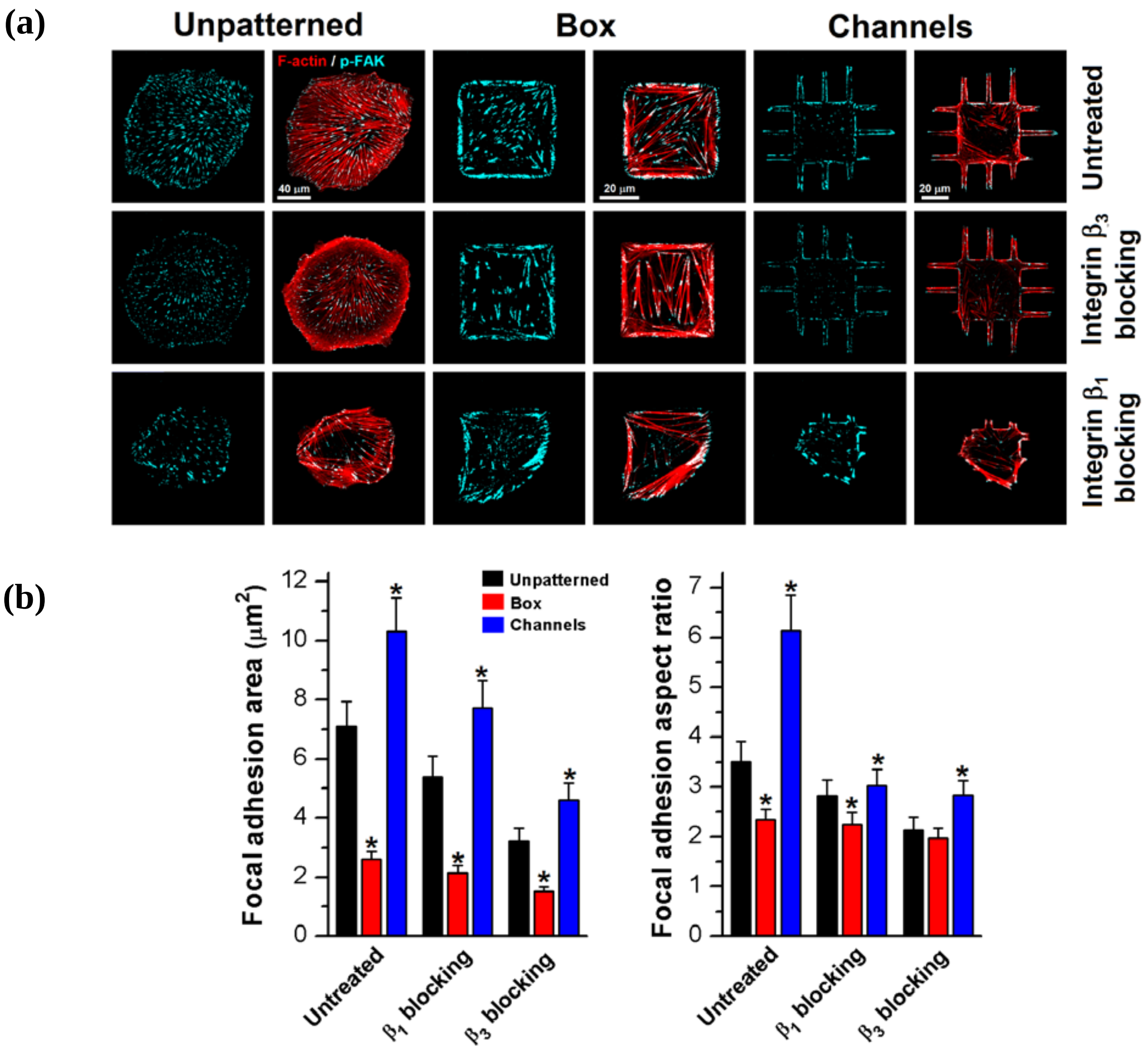

Supplementary Figure 12: Spatial analysis of FAK activation in unpatterned and micropatterned podocytes. **(a)** Podocytes were plated on unpatterned, box, or channel surfaces in the presence and absence of integrin blocking antibodies, and subsequently stained for phospho-FAK (green) and F-actin (red). For untreated cells, focal adhesions formed within the channels were larger than those in cells plated on box or unpatterned surfaces. Podocytes treated with β_3 blocking antibody show no morphological aberrations; however, the focal adhesions were much smaller than control cells. In contrast, podocytes treated with β_1 blocking antibody show smaller spreading areas with no effect on the size and location of focal adhesions. **(b)** Quantitative analysis of focal adhesion area and aspect ratio in podocytes plated on unpatterned, box, and channel surfaces and treated with either β_1 or β_3 blocking antibodies. Values given as mean ± SEM; n > 500, chosen randomly from 20 different cells taken from four different slides cultured independently (^$p < 0.05$, ***$p < 0.01$ vs. UNP).

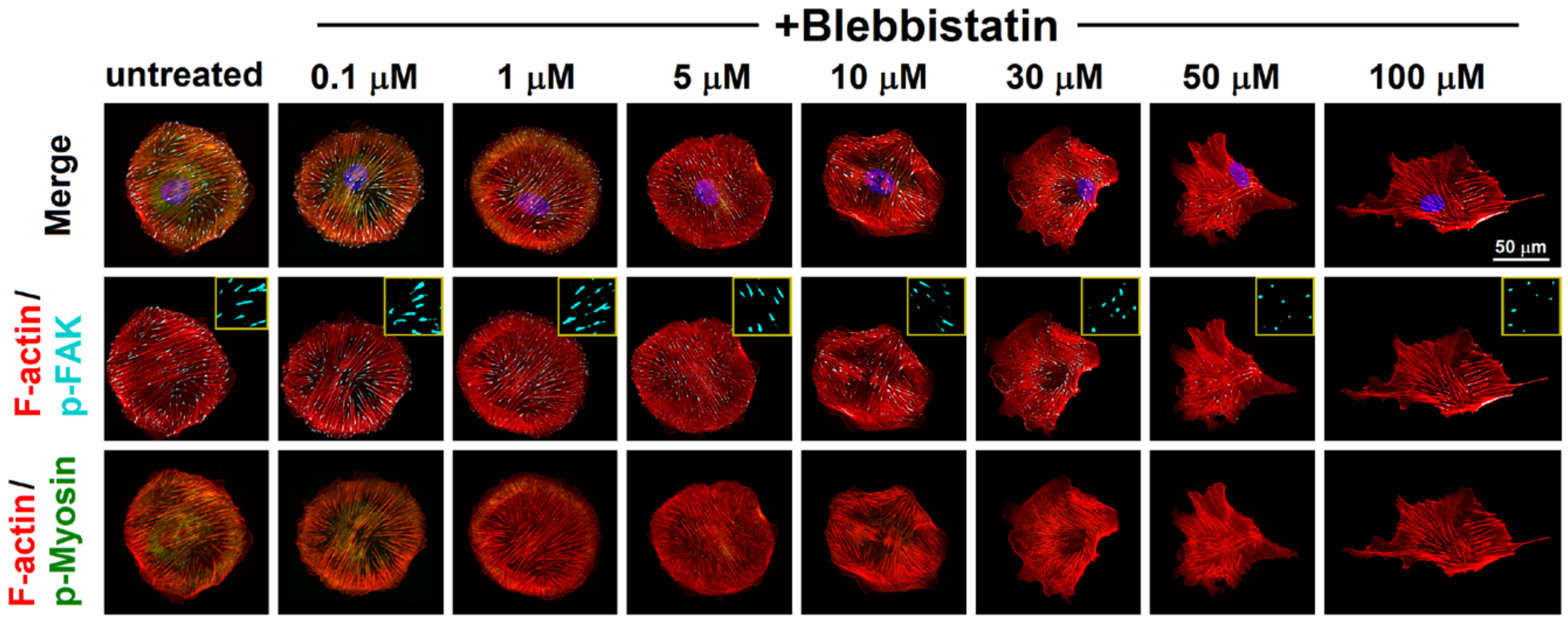

Supplementary Figure 13: Effect of blebbistatin on unpatterned podocytes. Unpatterned podocytes were treated with varying concentrations of blebbistatin from 0.1 to 100 µM for 12 hours prior to fixation and stained for F-actin (red), p-FAK (cyan), and p-myosin (green). Phospho-myosin intensities decreased gradually with increasing blebbistatin concentration, whereas cell shape and recruitment of p-FAK to focal adhesions were unaffected by concentrations lower than 10 µM.

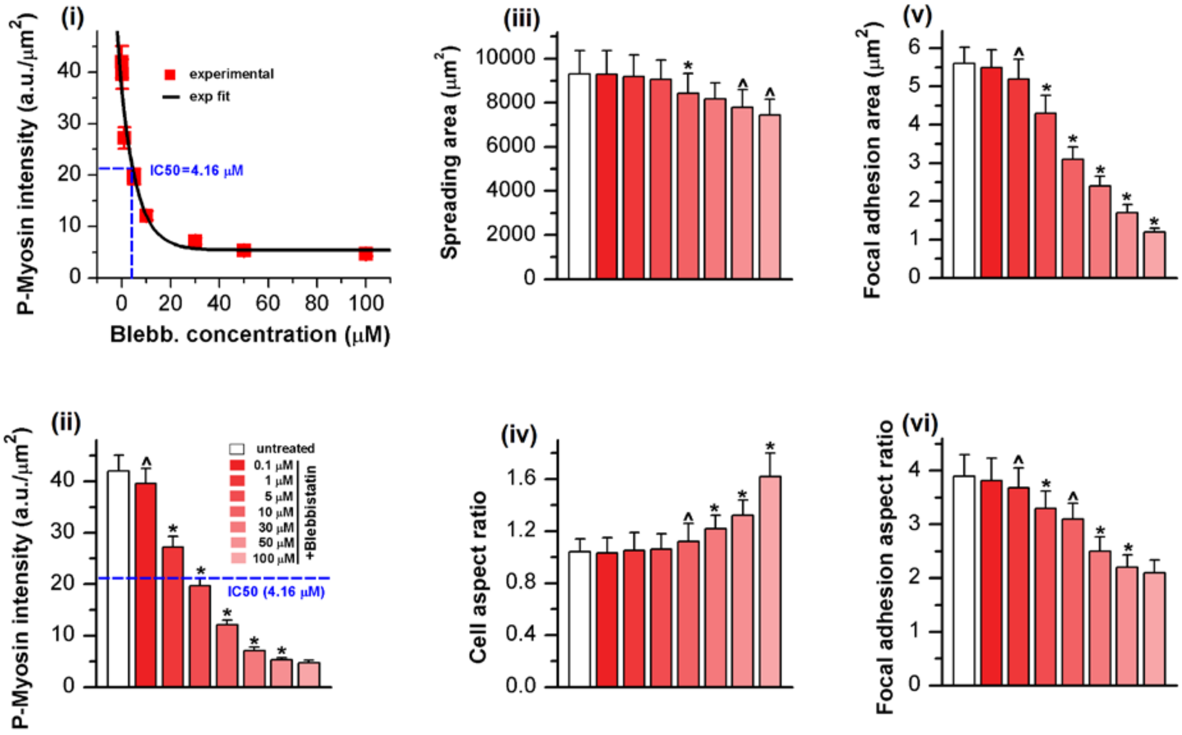

Supplementary Figure 14: Quantification of p-myosin levels in unpatterned podocytes treated with blebbistatin. As the concentration of blebbistatin increased, p-myosin intensity decreased gradually. Using exponential fit, calculated IC50 value was 4.16 μM, which agrees with prior measurements. At 10 μM, changes to the cell shape were minimal whereas changes to the focal adhesions were significant as early as 1 μM. Values given as mean ± SEM; n=20, chosen randomly from five different slides cultured independently (^$p < 0.05$, *$p < 0.01$ vs. previous concentration; one-way ANOVA with post-hoc Tukey).

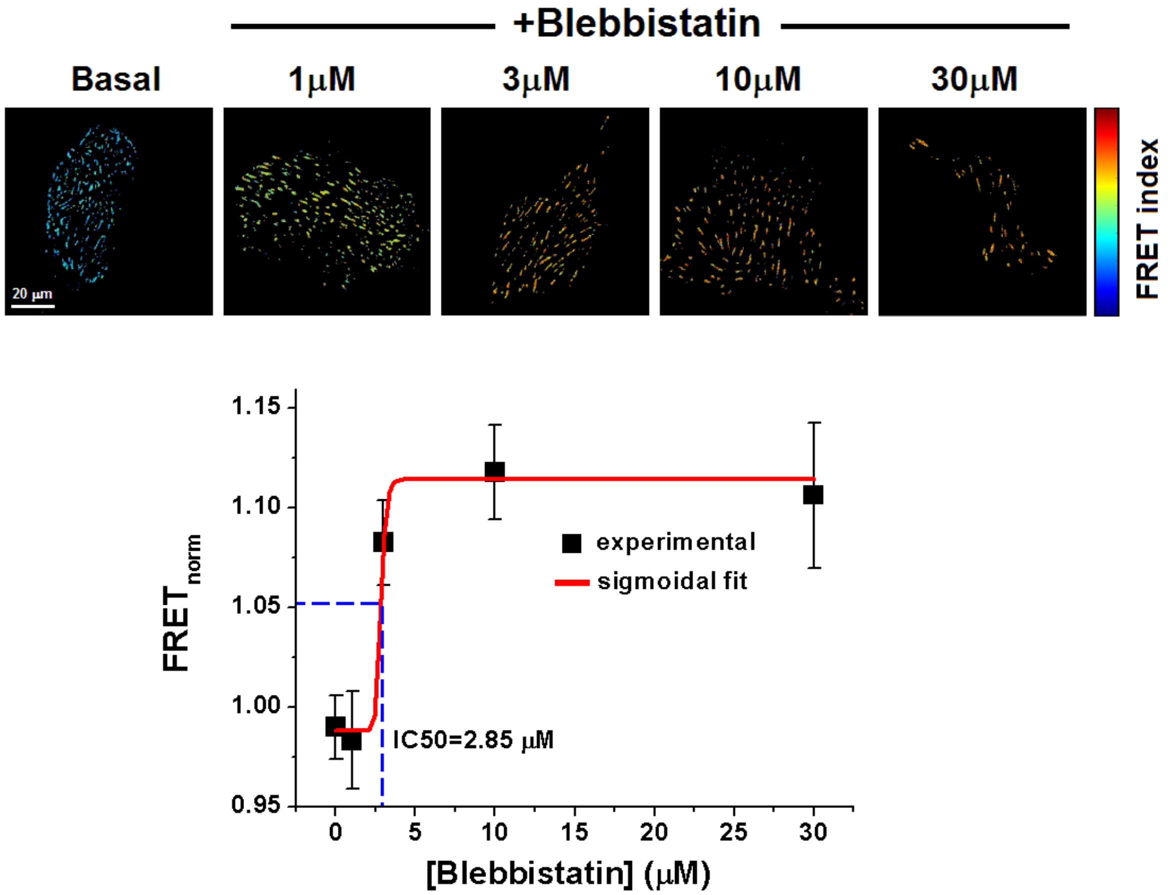

Supplementary Figure 15: Dose-dependent force response of podocytes to blebbistatin as measured by FRET vinculin tension sensor (VinTS). Human podocytes expressing VinTS fluorescent biosensor were treated with blebbistatin at varying concentrations (0, 1 µM, 3 µM, 10 µM and 30 µM) for two hours. **(Top panel)** Representative cells treated with the indicated blebbistatin concentration, showing normalized FRET ($FRET_{norm}$) indices of individual FAs. **(Bottom panel)** Dose response curve of mean $FRET_{norm}$ values from individual focal adhesions, signifying loss of traction forces with increasing blebbistatin concentrations. Sigmoidal fit shows half-maximal force disruption (IC_{50}) at 2.85 µM. (Mean ± SEM shown, n = 13-30 cells, >30 FAs per group).

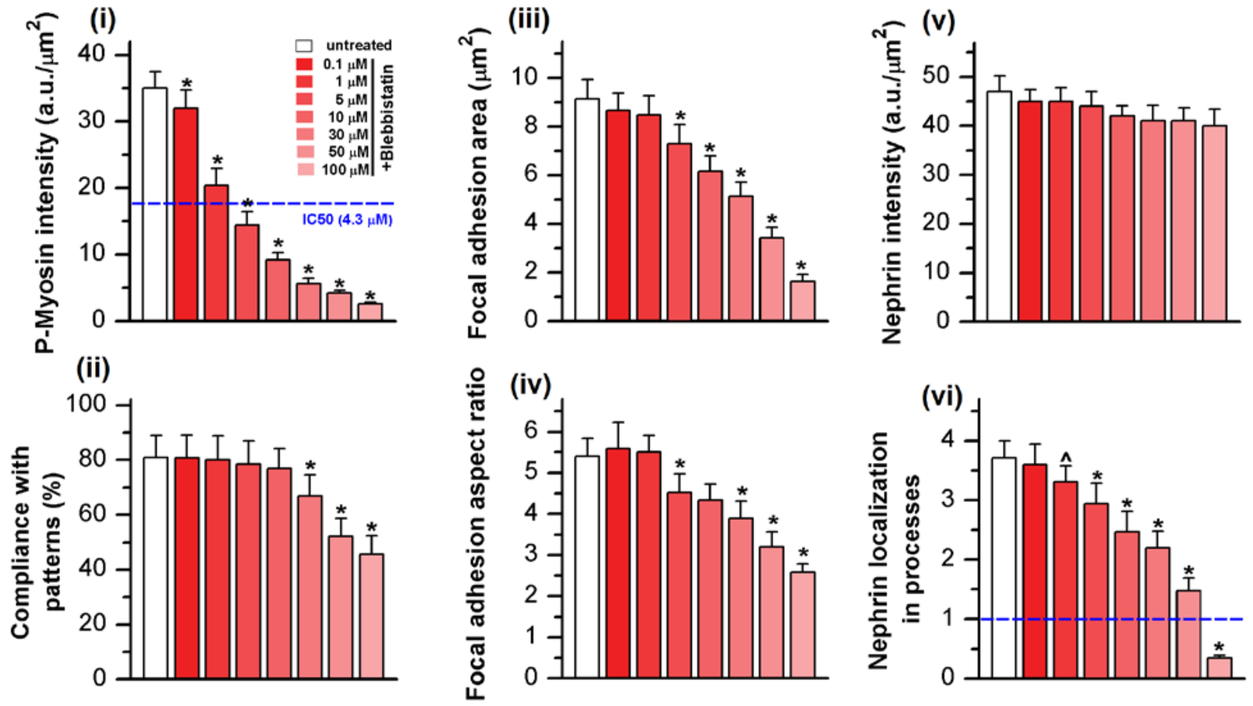

Supplementary Figure 16: Quantification of blebbistatin effects on patterned podocytes. As the concentration of blebbistatin increased, p-myosin intensity decreased gradually. This minimally affected recruitment of p-FAK to focal adhesion sites up to 10 μM. Nephrin localization showed a moderate decrease. At high concentration (100 μM), cell spreading and focal adhesion morphology showed a dramatic decrease leads to severe reduction in nephrin localization (<1). Values given as mean ± SEM; n=20, chosen randomly from five different slides cultured independently (^$p < 0.05$, *$p < 0.01$ vs. previous concentration; one-way ANOVA with post-hoc Tukey).

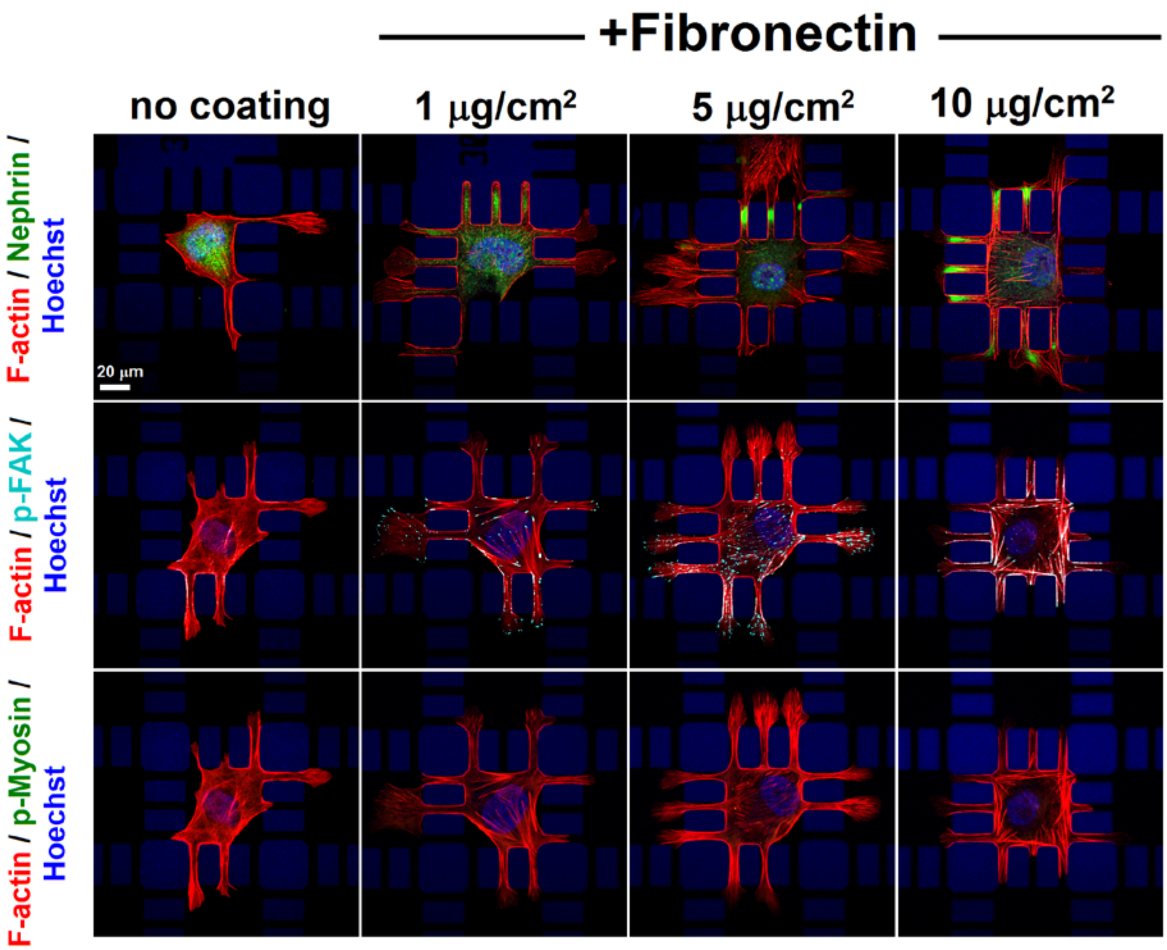

Supplementary Figure 17: Effect of fibronectin on micropatterned podocytes. Representative images of podocytes plated on channel micropatterns coated with fibronectin and treated with 100 µM of blebbistatin for 12 hours prior to fixation. Cells were stained either for nephrin (green) and F-actin (red) **(Top)**, p-FAK (cyan) and F-actin (red) **(Middle)**, or p-myosin (green) and F-actin (red) **(Bottom)**. Compliance with the micropatterns, recruitment of p-FAK to focal adhesions, and nephrin localization increased with fibronectin concentration, independent of myosin inhibition.

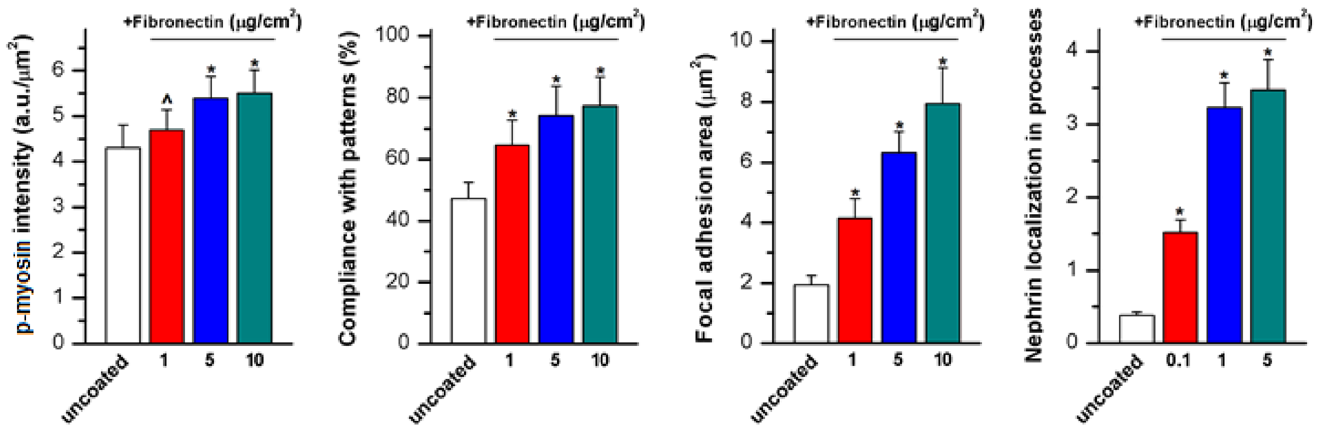

Supplementary Figure 18: Quantification of the effect of blebbistatin on podocytes plated on fibronectin-coated channel micropatterns. As the concentration of fibronectin increased, compliance of the cells with the patterns got higher, which in turn increased focal adhesion maturation. Nephrin localization within the processes was almost intact while cells plated on uncoated patterns showed insignificant localization. Values given as mean ± SEM; n=20, chosen randomly from 5 different slides cultured independently (^$p < 0.05$, *$p < 0.01$ vs. previous concentration; one-way ANOVA with post-hoc Tukey).

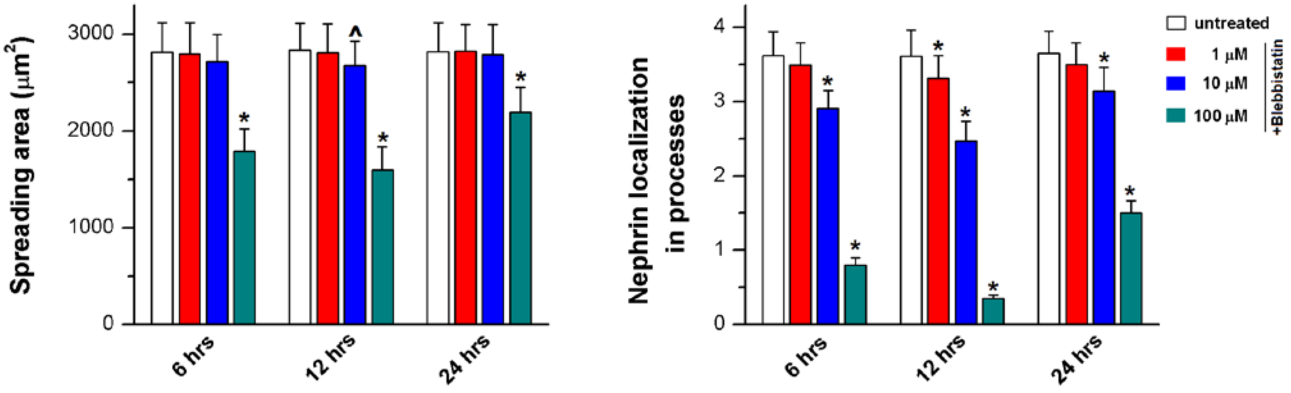

Supplementary Figure 19: Effect of blebbistatin concentration and incubation time on **(left)** cell spreading and **(right)** nephrin localization in peripheral processes. All measurements were performed on podocytes cultured on channel micropatterns for five days prior to the inhibition treatment. Values given as mean ± SEM; n=20, chosen randomly from five different slides cultured independently (^$p < 0.05$, *$p < 0.01$ vs. untreated, one-way ANOVA comparisons independent per time with post-hoc Tukey).

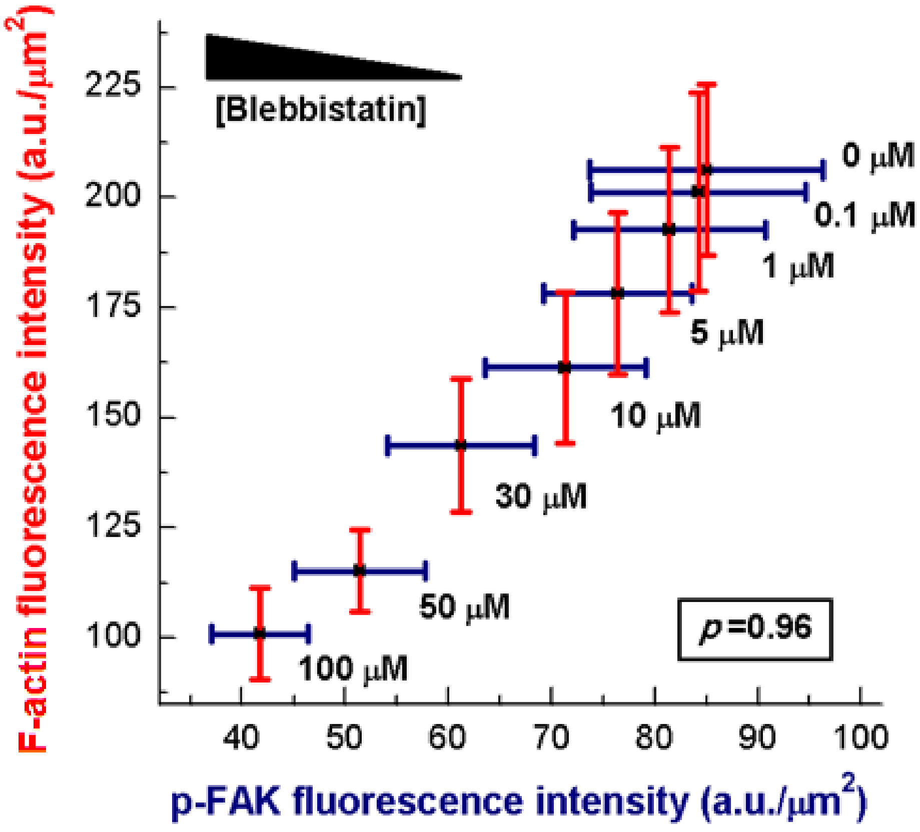

Supplementary Figure 20: Correlation between p-FAK (active FAK) and F-actin intensities within the focal adhesions in micropatterned podocytes. Strong correlation suggests that in micropatterned podocytes, focal adhesion maturation and morphology were mainly regulated by stress fiber architecture and less by contractile tension.

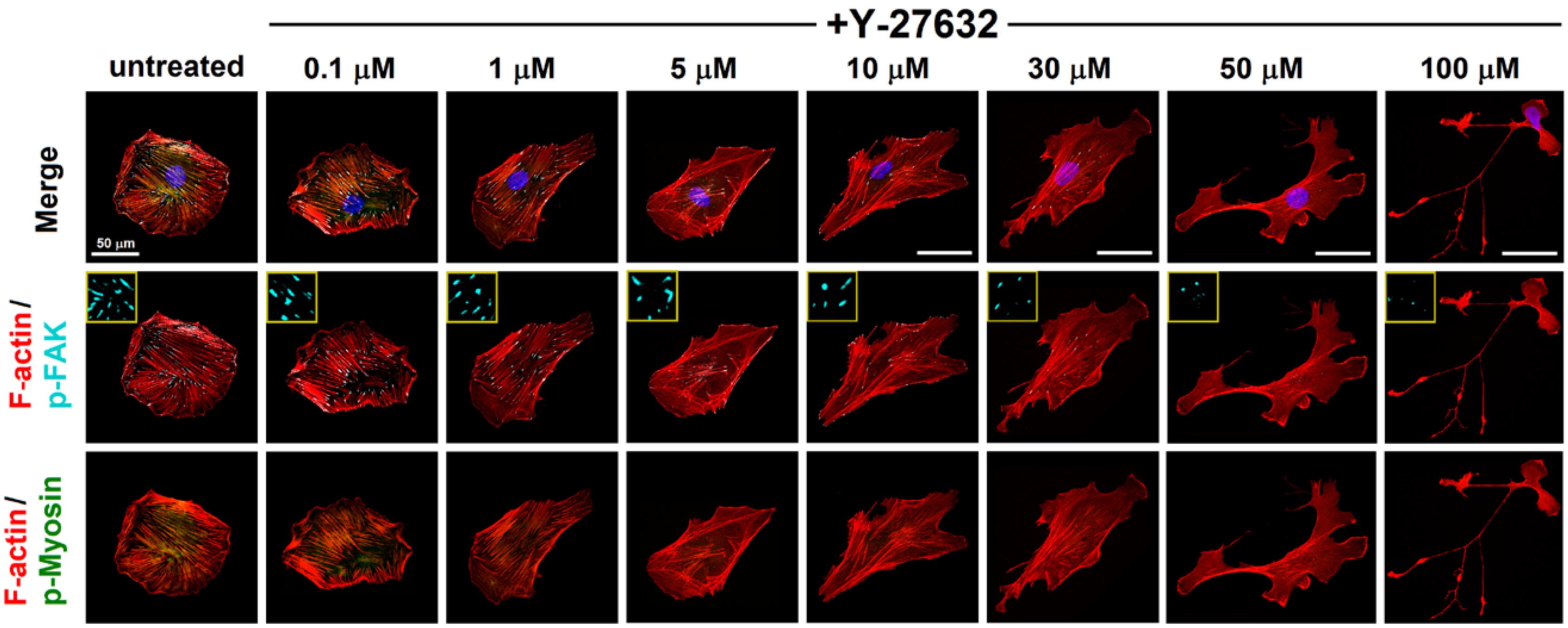

Supplementary Figure 21: Effect of Y-27632 on unpatterned podocytes. Unpatterned podocytes were treated with varying concentrations of the ROCK inhibitor Y-27632 from 0.1 to 100 μM for 12 hours prior to fixation and stained for F-actin (red), p-FAK (cyan), and p-myosin (green). Phospho-myosin intensity decreased gradually with increasing blebbistatin concentration, whereas cell shape and recruitment of p-FAK to focal adhesions were unaffected by blebbistatin up to 1 μM.

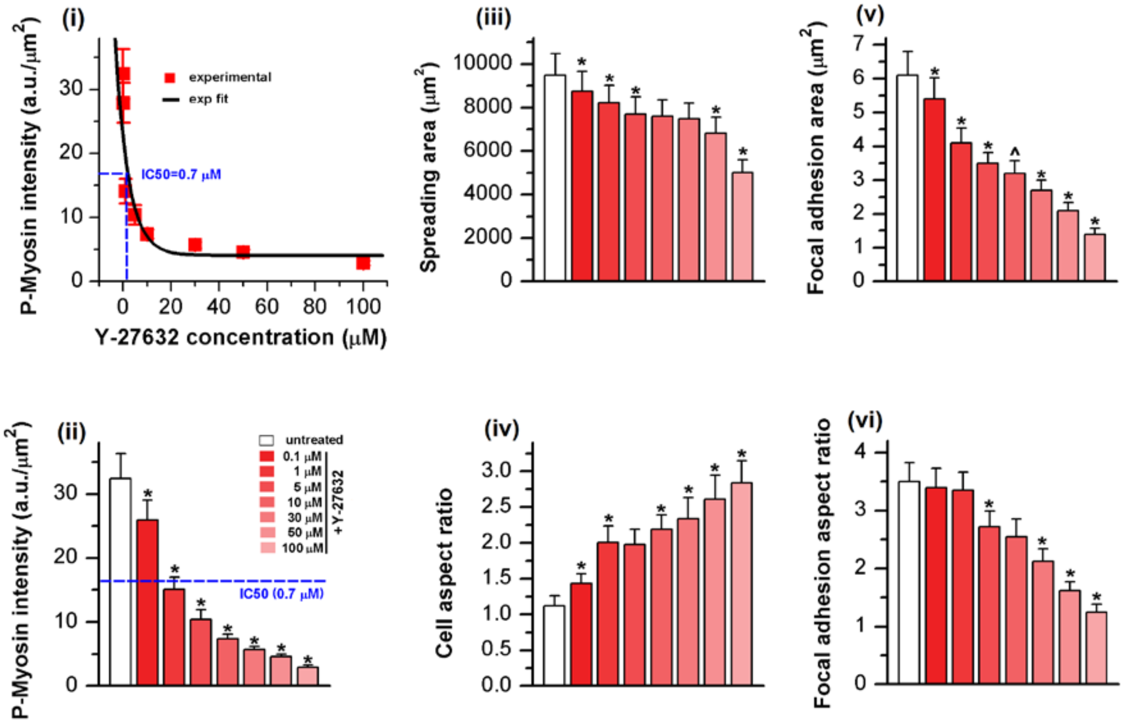

Supplementary Figure 22: Quantification of the effects of the ROCK inhibitor Y-27632 on unpatterned podocytes. IC50 value was 0.7 μM similar to previously reported values. Changes to cell shape were insignificant up to 5 μM whereas focal adhesions were affected at lower concentrations. Values given as mean ± SEM; n=20, chosen randomly from five different slides cultured independently (^p < 0.05, *p < 0.01 vs. previous concentration; one-way ANOVA with post-hoc Tukey).

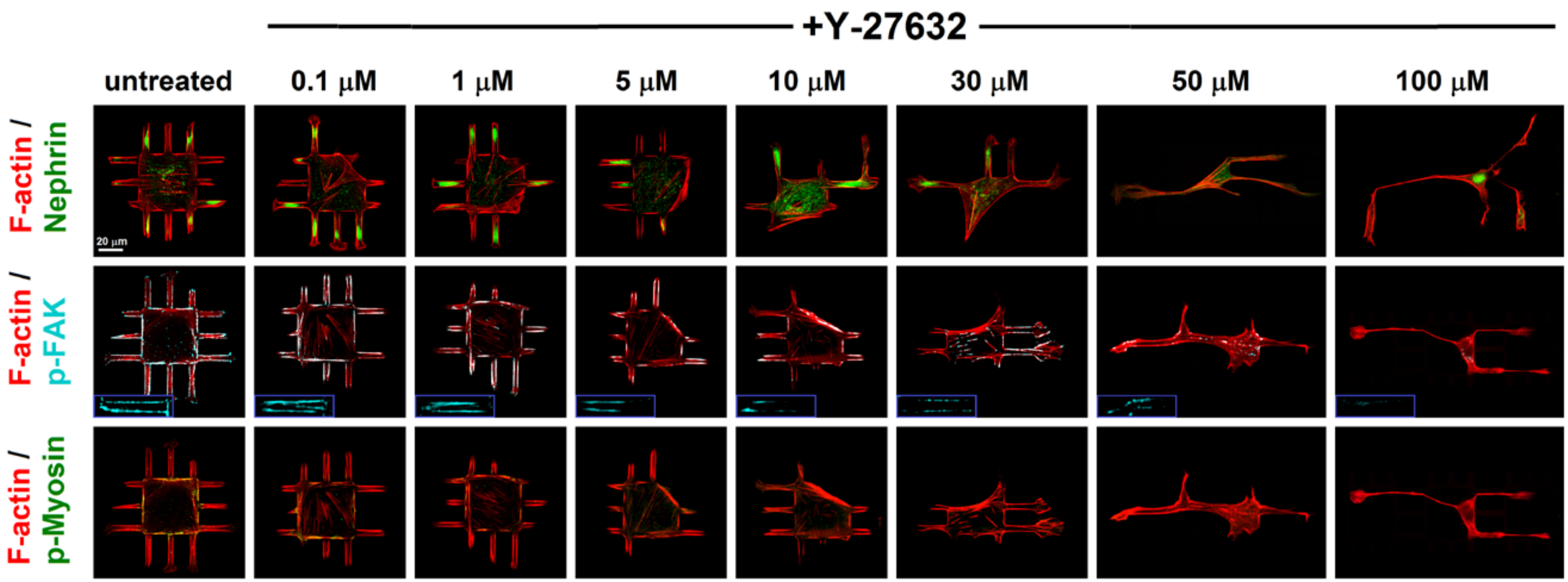

Supplementary Figure 23: Effect of Y-27632 on micropatterned podocytes. Micropatterned podocytes were treated with varying concentrations of the ROCK inhibitor Y-27632 from 0.1 to 100 μM for 12 hours prior to fixation and stained either for nephrin (green) and F-actin (red) **(Top)**, p-FAK (cyan) and F-actin (red) **(Middle)**, or p-myosin (green) and F-actin (red) **(Bottom)**.

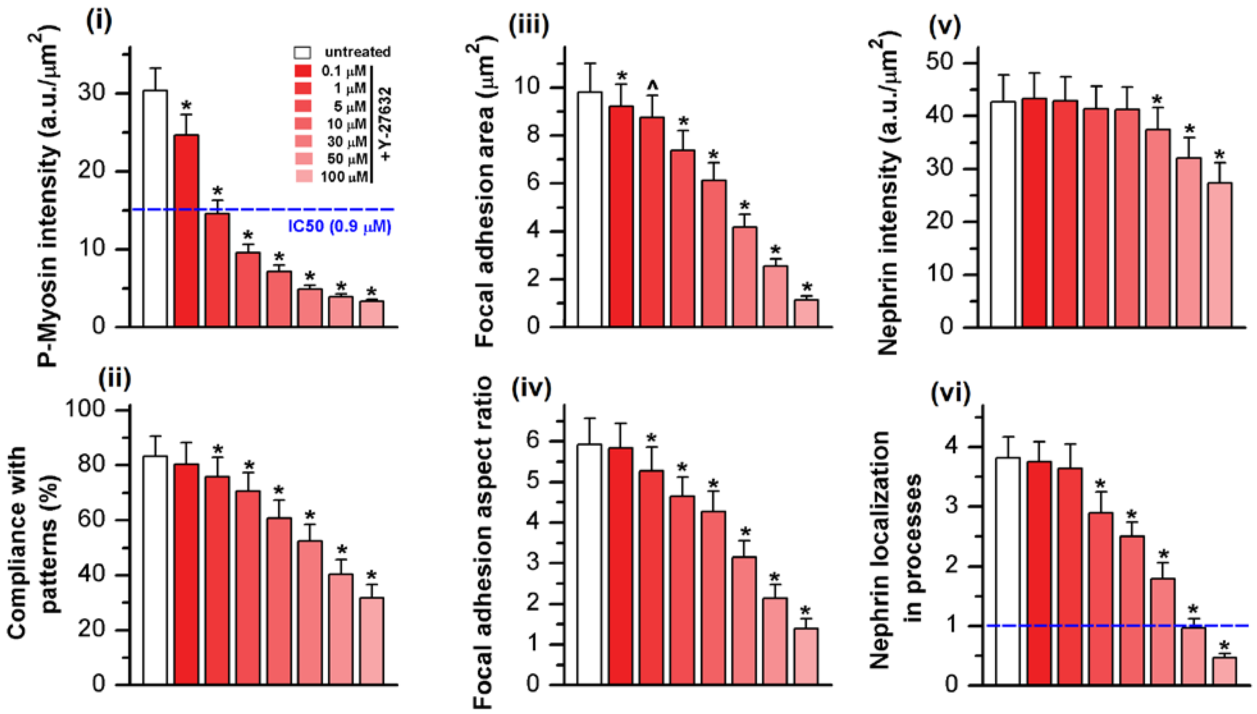

Supplementary Figure 24: Quantification of the ROCK inhibitor Y-27632 effects on micropatterned podocytes. As the concentration of blebbistatin increased, p-myosin intensity decreased gradually. This minimally affected recruitment of p-FAK to focal adhesion sites up to 5 μM. Nephrin localization showed a moderate decrease. At higher concentrations (>5 μM), cell spreading and focal adhesion morphology and nephrin localization were all affected significantly. Values given as mean ± SEM; n=20, chosen randomly from five different slides cultured independently (^p < 0.05, *p < 0.01 vs. previous concentration; one-way ANOVA with post-hoc Tukey).

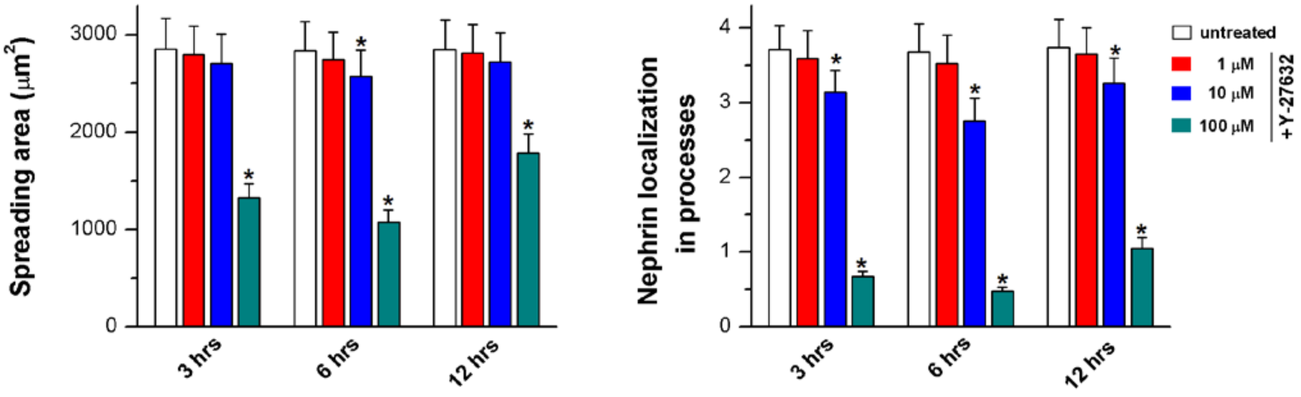

Supplementary Figure 25: Effect of Y-27632 concentration and incubation time on: **(Left)** cell spreading, and **(Right)** nephrin localization in peripheral processes. All measurements were performed on podocytes cultured on 3-D biochips for five days prior to treatment. Values given as mean ± SEM; n=20, chosen randomly from five different slides cultured independently (^p < 0.05, *p < 0.01 vs. untreated; one-way ANOVA for independent time points with post-hoc Tukey).

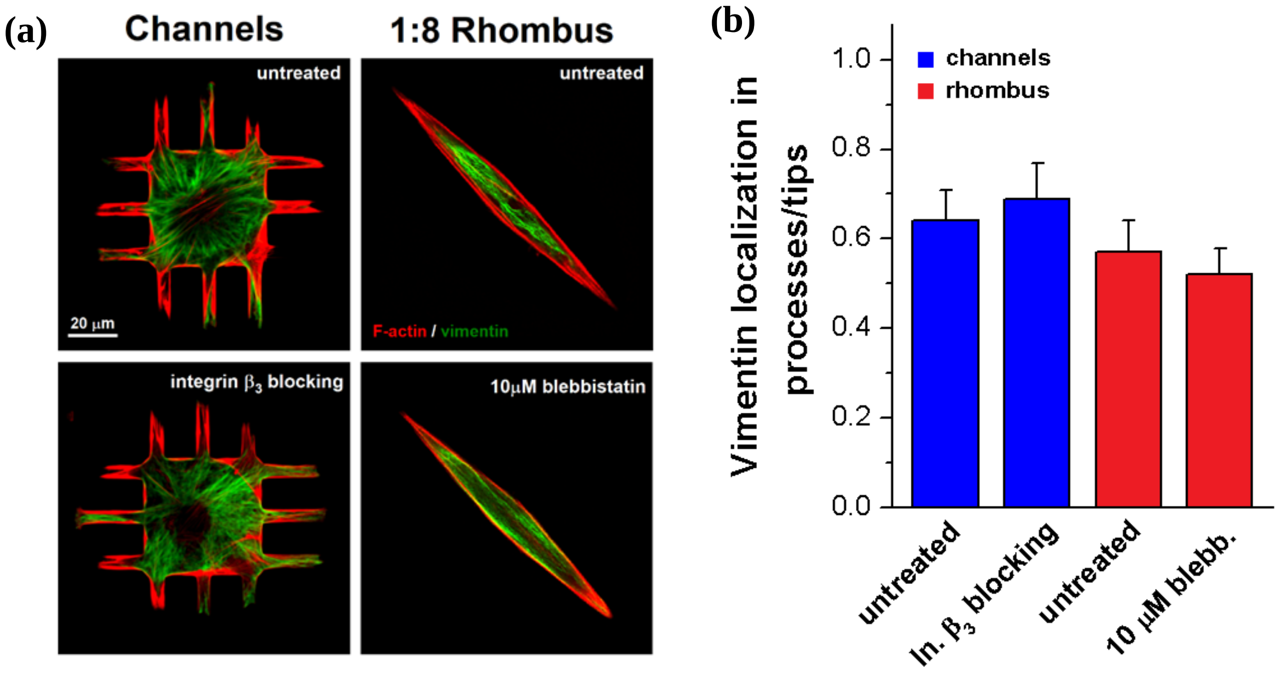

Supplementary Figure 26: Analysis of vimentin localization in micropatterned podocytes. **(a)** Representative immunofluorescence images showing podocytes plated on channel or rhombus micropatterns, treated either with integrin β_3 blocking antibodies or 10 µM blebbistatin, fixed after five days in culture, and stained for vimentin (green) and F-actin (red). **(b)** Quantitative analysis of vimentin localization in micropatterned podocytes treated with either integrin β_3 blocking antibodies (channels) or 10 µM blebbistatin (rhombus). Values given as mean ± SEM; n = 30 chosen randomly from three different slides cultured independently.

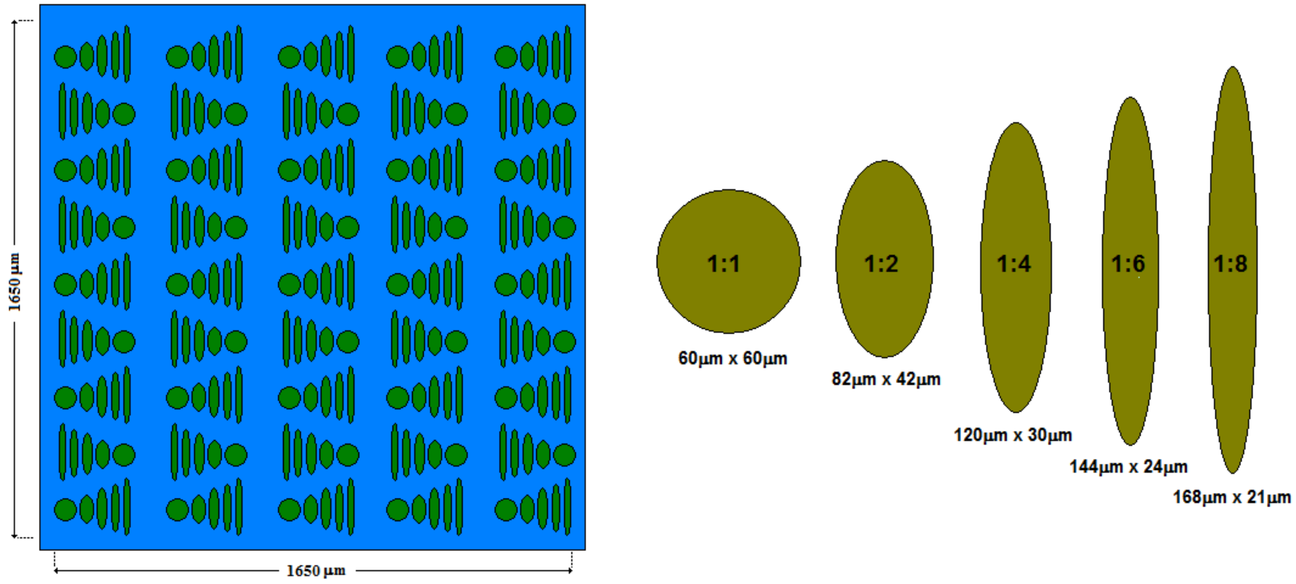

Supplementary Figure 27: (Left) Architecture of the ellipsoid micropatterns, and **(Right)** the geometrical characteristics of a single ellipsoid array.

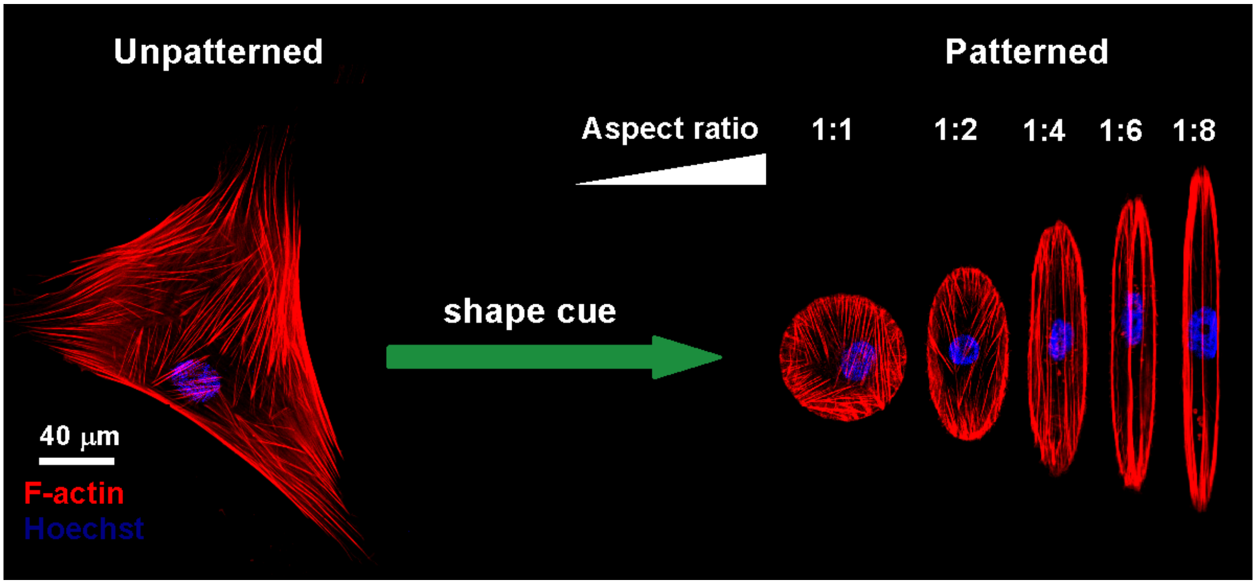

Supplementary Figure 28: Micropatterning of vascular smooth muscle cells (SMC). A typical SMC in culture showing hypertrophic morphology. Microfabrication was used to construct an array of ellipsoid shapes with constant spreading area and varying aspect ratios. SMCs plated on elongated ellipsoid shapes present morphologies closer to their *in vivo* state as characterized by the spindle shape. Cells were stained for F-actin (red) and nuclei (blue).

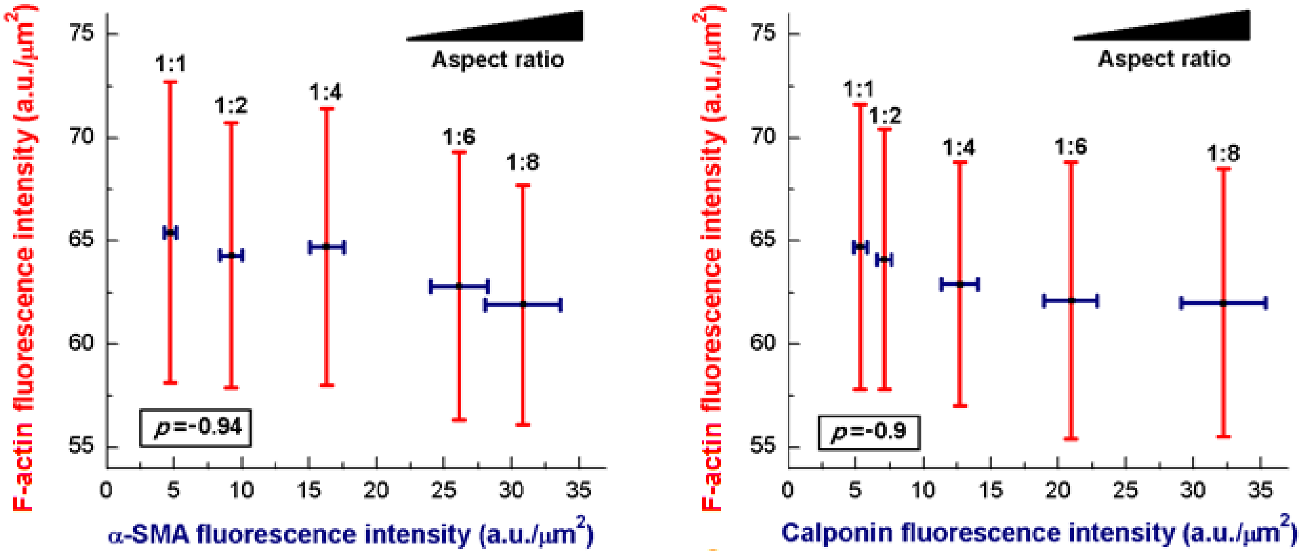

Supplementary Figure 29: Correlation between α-SMA (left) and calponin (right) with F-actin intensities in micropatterned SMCs. Negative correlation suggests that expression of α-SMA and calponin are independent of stress fiber formation as a function of the cell aspect ratio.

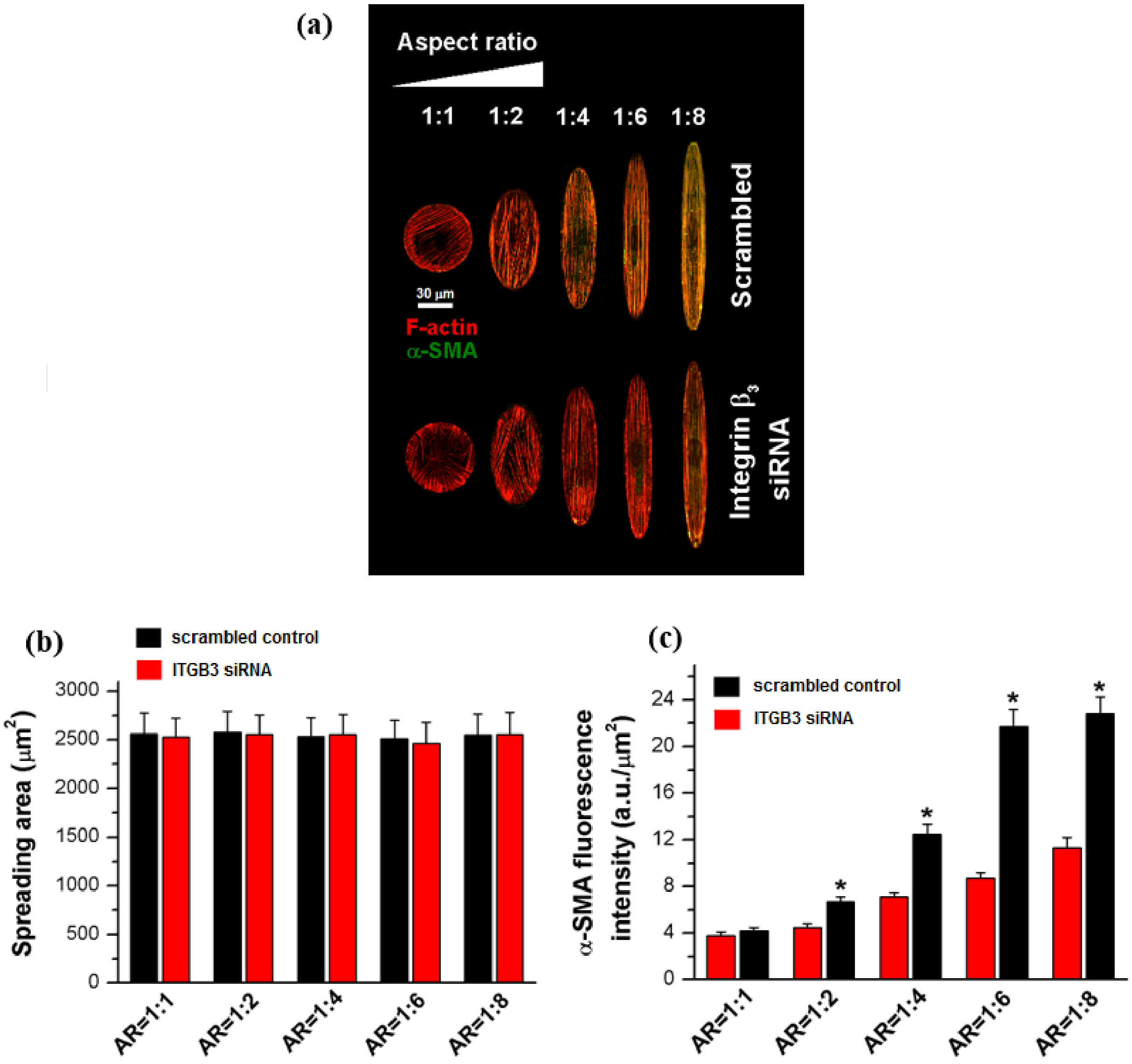

Supplementary Figure 30: Quantitative analyses of spreading area and α-SMA expression in SMCs plated on ellipsoid micropatterns and treated either with siRNA for integrin β_3 (ITGB3) or with scrambled siRNA (control). **(a)** Representative images showing F-actin (red) and α-SMA (green) in ITGB3 and control SMCs plated on ellipsoid micropatterns. In control SMCs, α-SMA expression increased with increasing aspect ratio. Under ITGB3 knockdown, α-SMA expression was roughly constant with increasing aspect ratio. **(b)** Spreading area of ITGB3 and scrambled control SMCs. No differences were observed between transfected and control cells. Values given as mean ± SEM; n = 80, chosen randomly from eight different slides cultured independently. **(c)** Quantitative analysis of α-SMA in ITGB3 and scrambled control SMCs plated on ellipsoid patterns. α-SMA expression increased with increasing aspect ratio while for ITGB3 cells the intensity levels less dependent on shape. Values given as mean ± SEM; n = 80, chosen randomly from four different slides cultured independently (*$p < 0.01$ vs. ITGB3 cells).

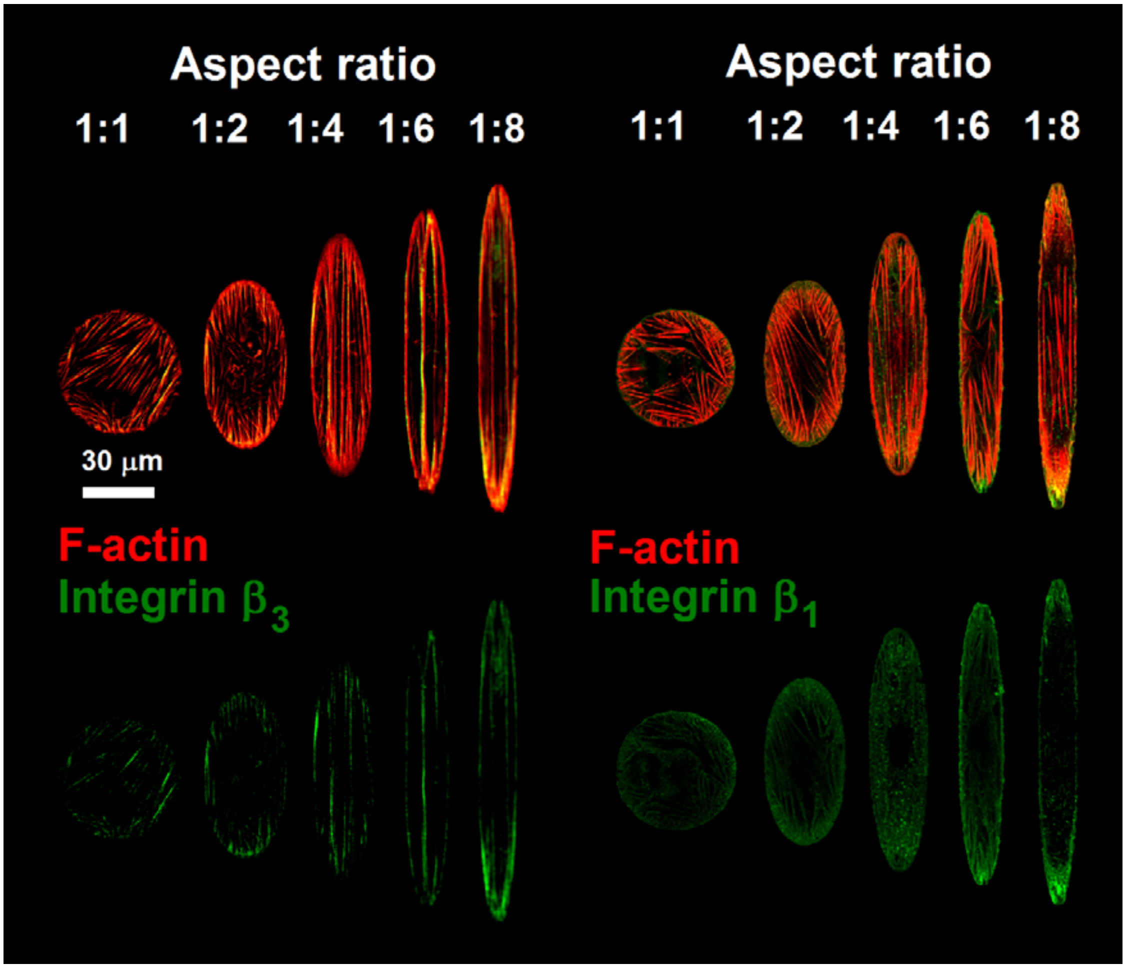

Supplementary Figure 31: Integrin staining in micropatterned SMCs. Representative images of SMCs plated on ellipsoid micropatterns and independently stained for either integrin β_3 or β_1 (green) and F-actin (red). Integrin β_3 showed expression along actin stress fibers for all tested shapes, while integrin β_1 was localized on the tips and cell periphery.

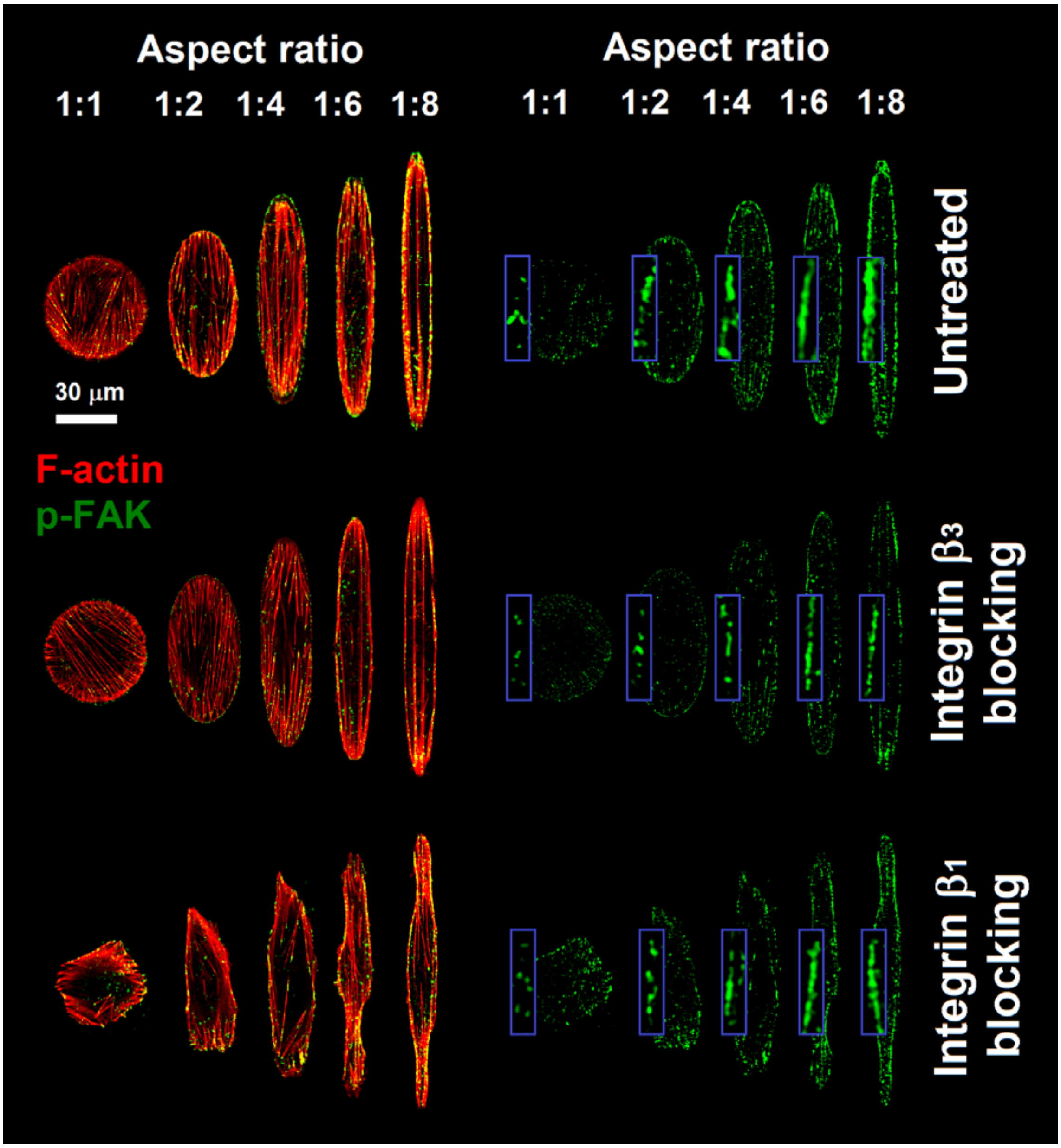

Supplementary Figure 32: p-FAK staining in micropatterned SMCs. Representative images of SMCs plated on the ellipsoid micropatterns, and stained for p-FAK (green) and F-actin (red). Untreated cells exhibited large focal adhesions along the cell periphery that aligned with actin bundles. Cells treated with β_1 blocking antibodies did not spread well; however, mature and large focal adhesions were detected with increasing aspect ratio. Those treated with integrin β_3 blocking antibodies showed compliance to the micropatterns; however, focal adhesions were smaller and less pronounced.

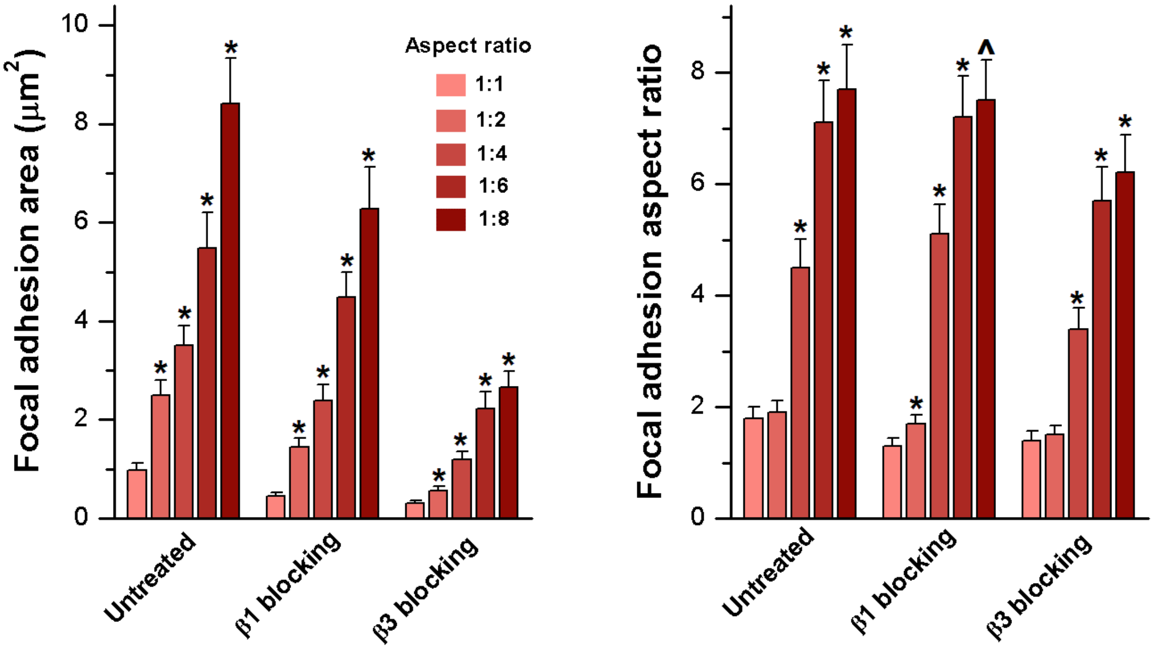

Supplementary Figure 33: Quantitative analysis of focal adhesion area and aspect ratio in SMCs plated on ellipsoid micropatterns and treated with either integrin β_1 or β_3 blocking antibodies. For the untreated cells, both area and aspect ratio increased with increasing aspect ratio of the cell. The same trend in focal adhesion area was observed for cells treated with integrin β_1 blocking antibody although focal adhesions area and aspect ratio were smaller. SMCs treated with integrin β_3 blocking antibody showed smaller focal adhesions. Values given as mean ± SEM; n > 500 focal adhesion sites, chosen from 20 random cells taken from four different slides, cultured independently (***p <0.01 vs. previous ratio).

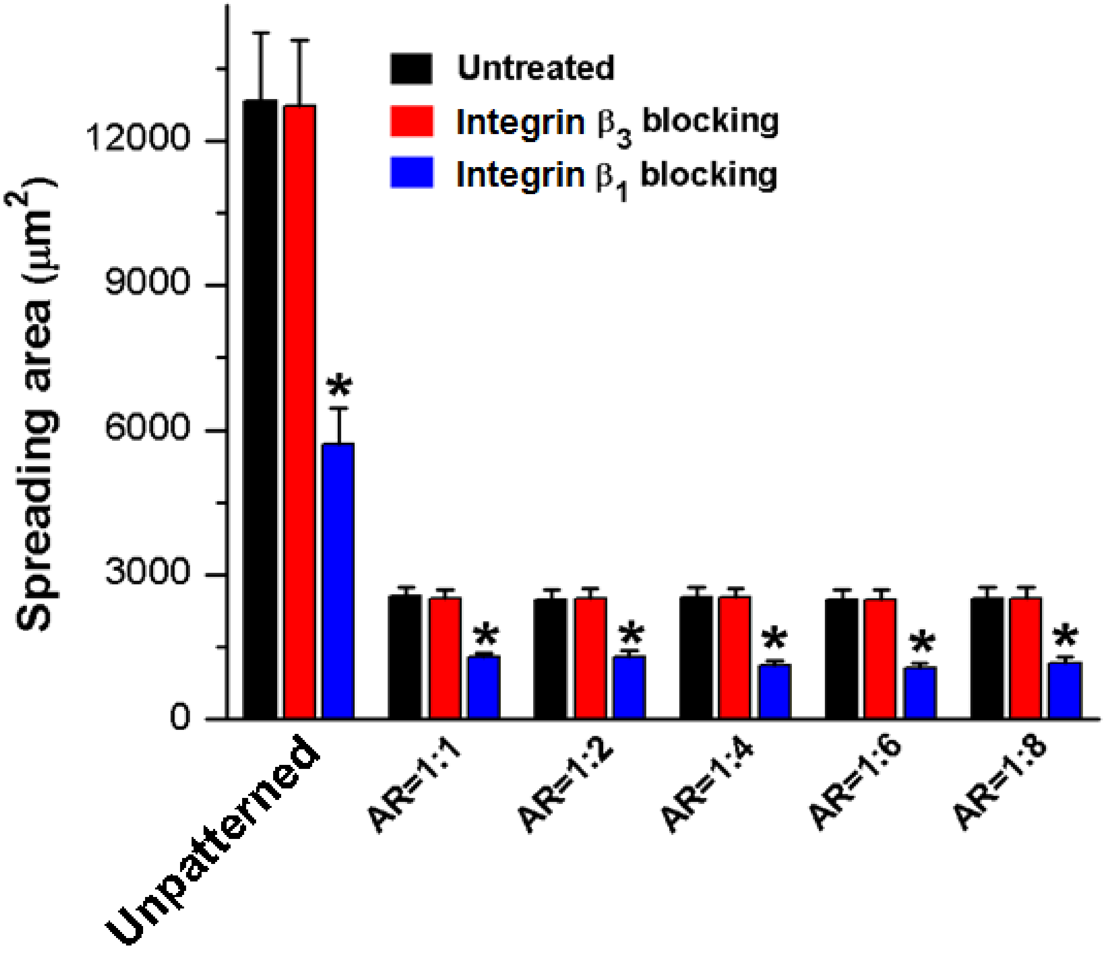

Supplementary Figure 34: Spreading areas of unpatterned and micropatterned SMCs with and w/o blocking of integrin β_3 or β_1, plated in micropatterns with varying aspect ratios (AR). Values given as mean ± SEM; n = 80, chosen randomly from eight different slides cultured independently (*****$p < 0.01$ vs. untreated; one-way ANOVA with post-hoc Tukey per each group).

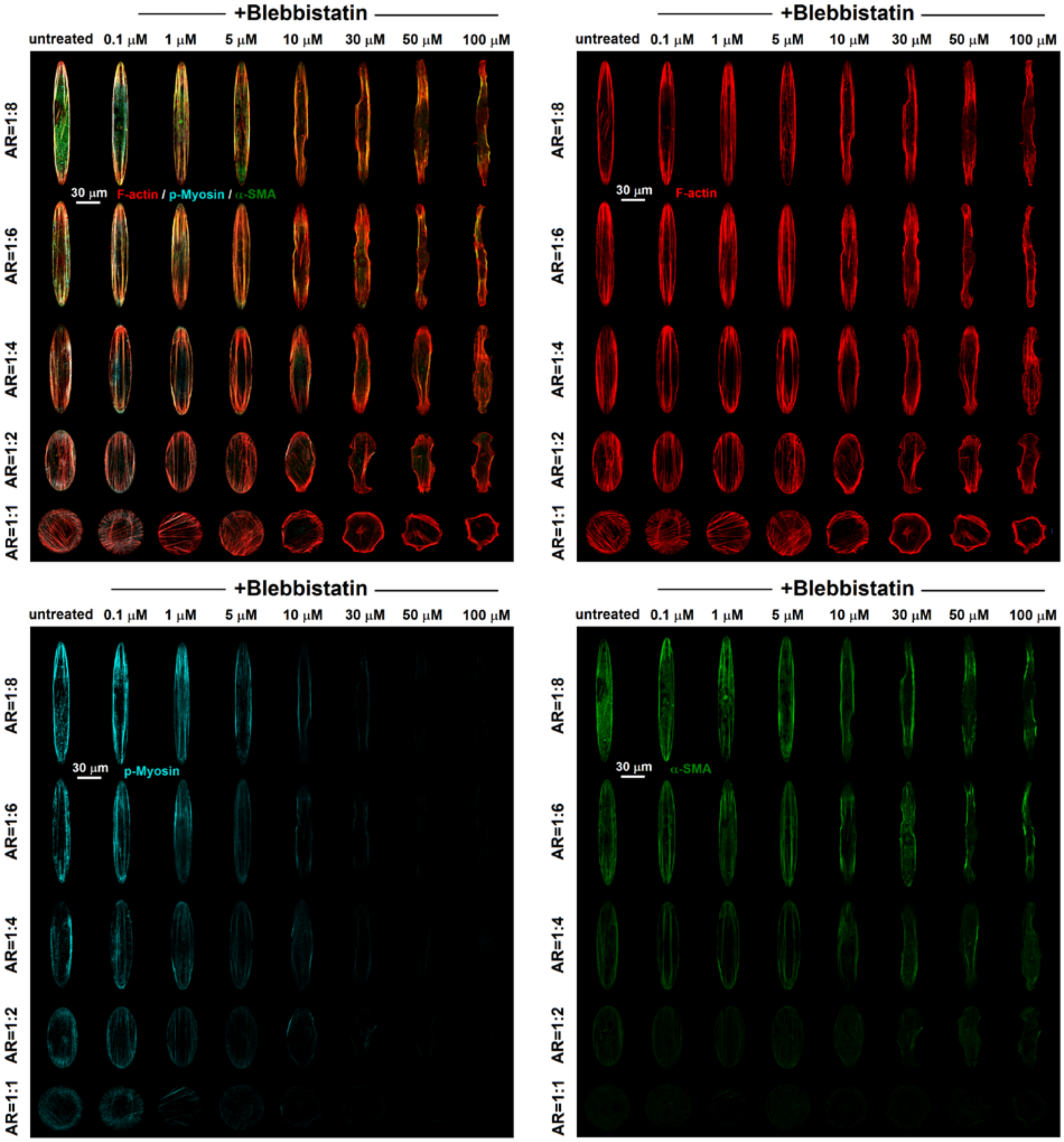

Supplementary Figure 35: Effect of blebbistatin on micropatterned SMCs. SMCs were treated with varying concentrations of blebbistatin from 0.1 to 100 μM for 12 hours prior to fixation and stained for F-actin (red), p-myosin (cyan) and α-SMA (green). Both p-myosin intensity and the compliance of the cells with the patterns decreased gradually with increasing blebbistatin concentration. α-SMA expression intensities were higher in patterned SMCs with increased aspect ratio and showed little change upon treatment with increasing blebbistatin concentration.

Note: This is an extended version of Figure 5 from the main text.

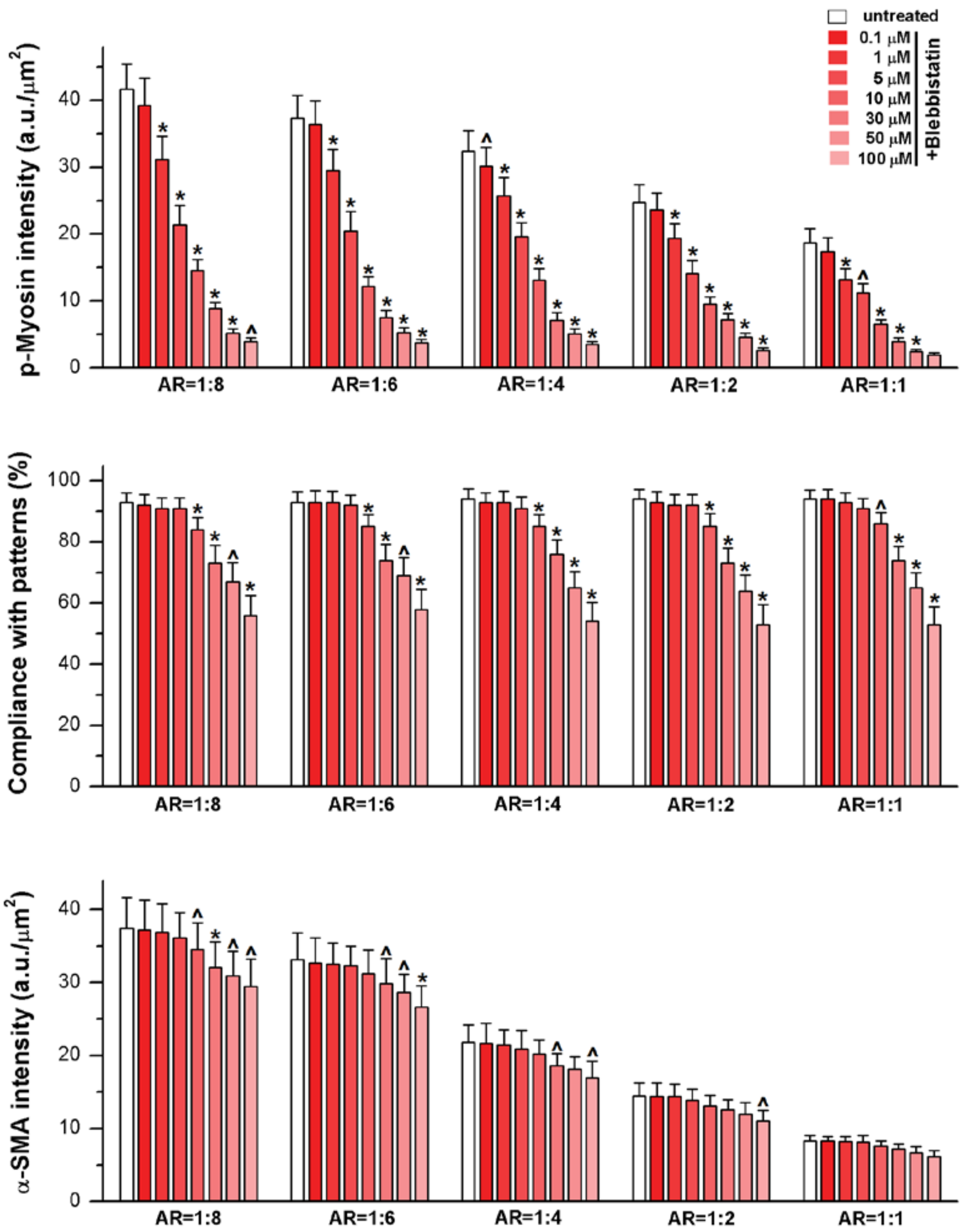

Supplementary Figure 36: Quantification of blebbistatin effects on micropatterned SMCs. Expression of α-SMA expressions correlated well with changes in cell shape. A clear trend between α-SMA intensity and the cell aspect ratio was observed, independent of blebbistatin concentration, suggesting that patterned SMCs regulated the expression of α-SMA, even when contractile tension was greatly lowered. Values given as mean ± SEM; n=20, chosen randomly from four different slides cultured independently (^$p < 0.05$, *$p < 0.01$ vs. previous concentration).

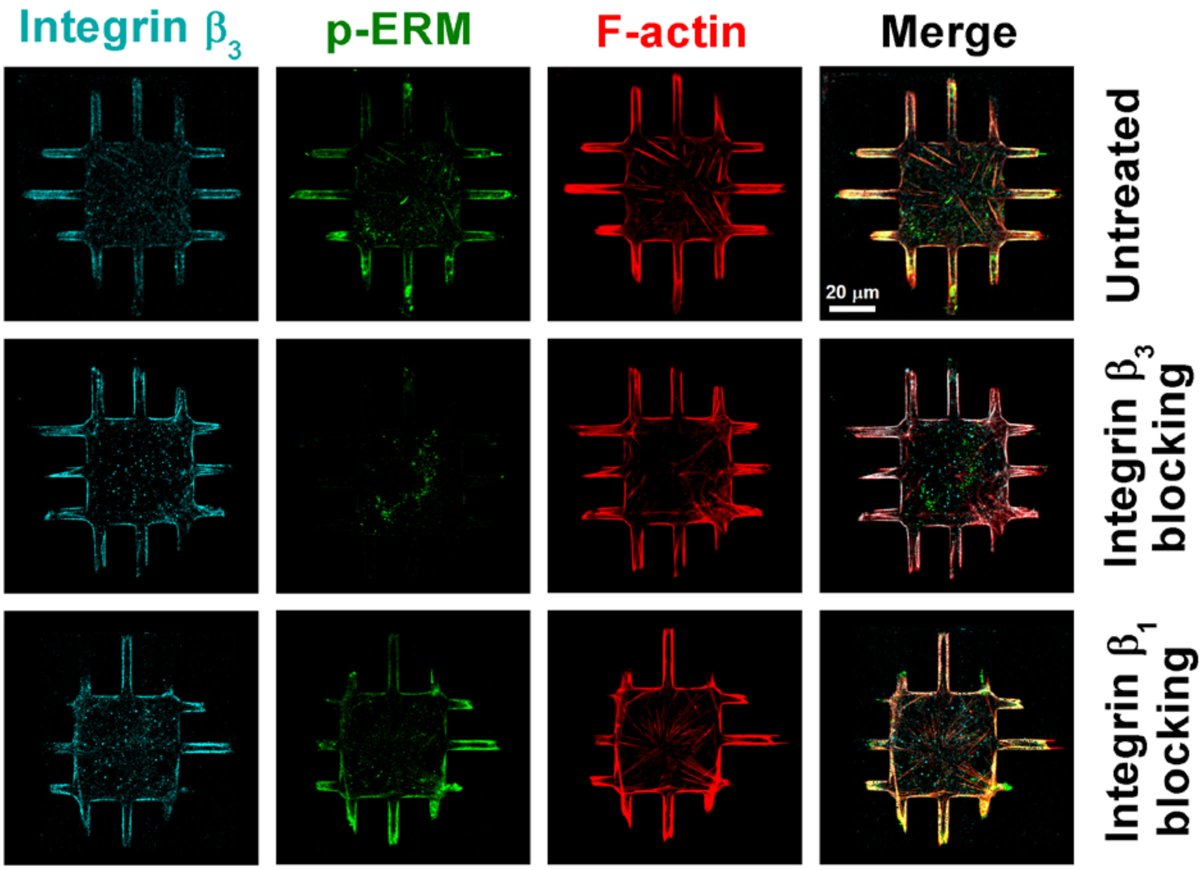

Supplementary Figure 37: Immunostaining p-ERM in micropatterned podocytes. Representative images of podocytes on channel micropatterns stained for F-actin (red), p-ERM (green), and integrin β_3 (cyan). p-ERM colocalized extensively with F-actin and integrin β_3 within the peripheral processes in untreated cells. Colocalization was observed in podocytes treated with β_1 blocking antibody; however, when podocytes were treated with β_3 blocking antibodies, colocalization was abolished, and p-ERM localized only within the cell body.

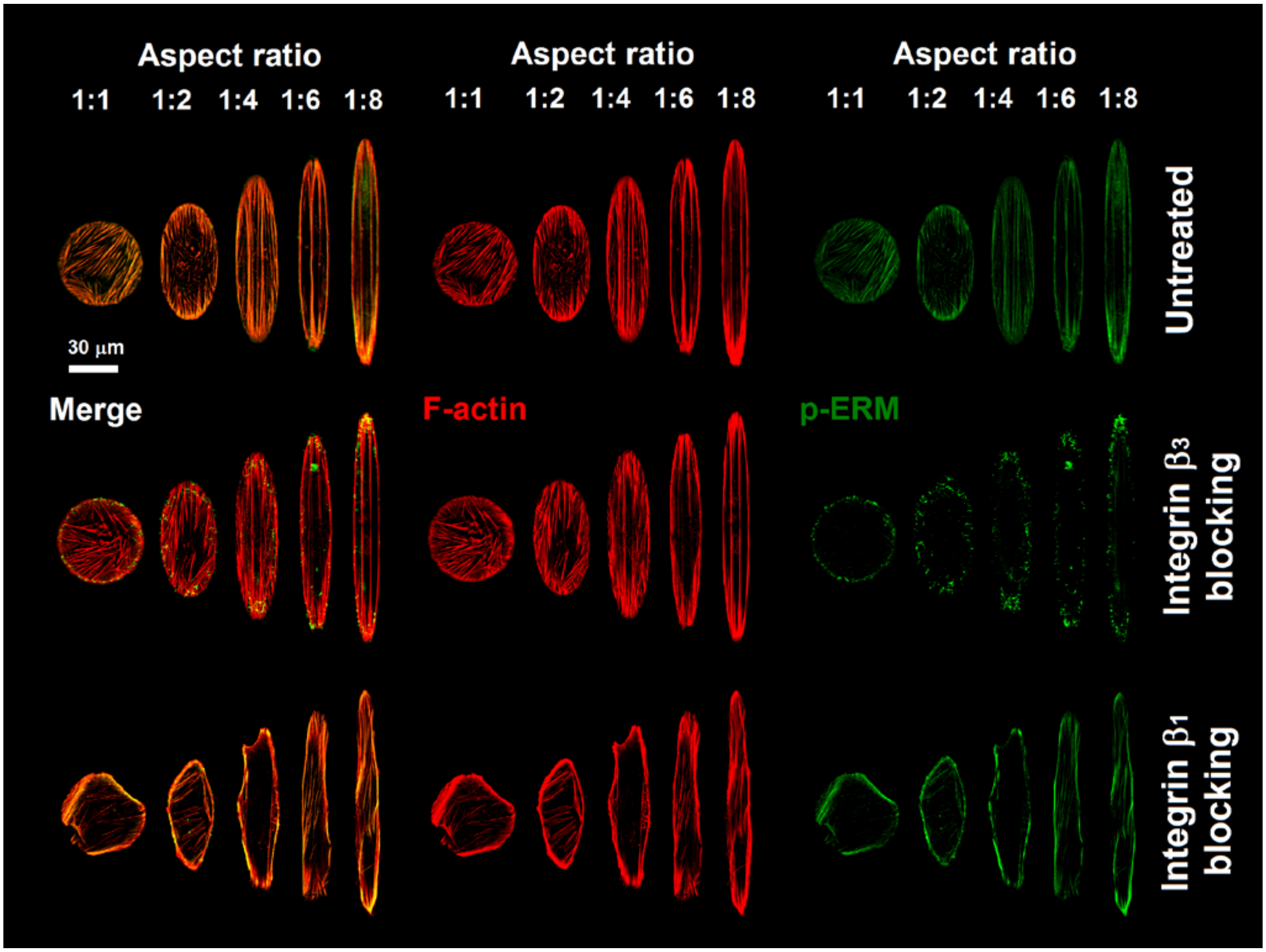

Supplementary Figure 38: Immunostaining for p-ERM in micropatterned SMCs. Representative images of SMCs on ellipsoid micropatterns with F-actin (red) and p-ERM (green) staining. For untreated cells, p-ERM colocalized with actin stress fibers. Cells treated with integrin β_3 blocking antibodies showed a significant change in the spatial distribution of p-ERM with little or no colocalization with actin fibers, whereas integrin β_1 blocking had no effect on p-ERM distribution despite its dramatic effect on cell spreading.

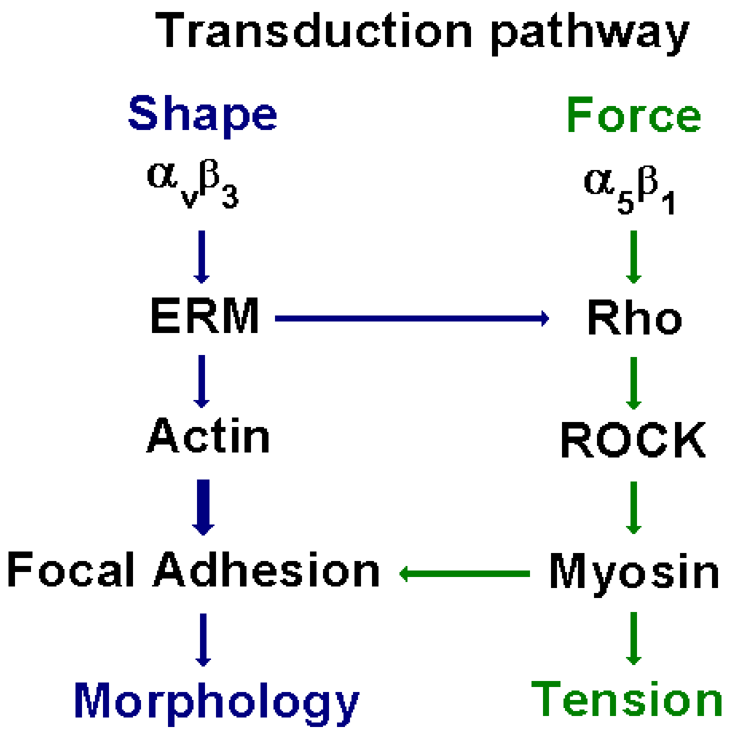

Supplementary Figure 39: Proposed minimal signaling network showing the interconnected pathways for tension and shape information processed by integrin β_1 and β_3.

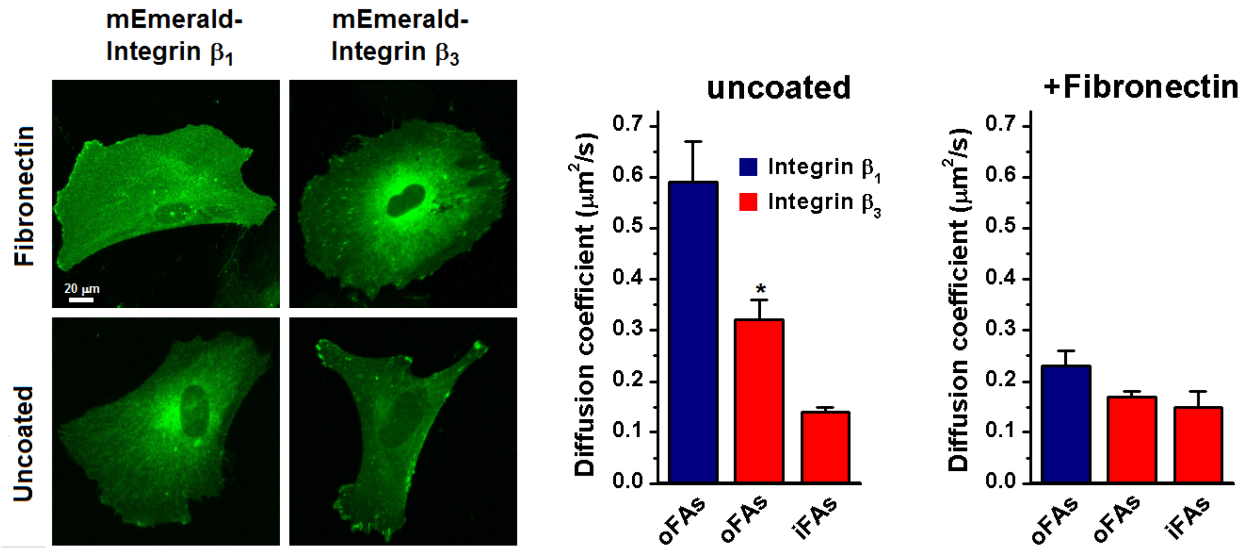

Supplementary Figure 40: Mobility of integrins in unpatterned podocytes. **(Left)** Representative images of podocytes transfected with either mEmerald-integrin β_1 or mEmerald-integrin β_3 and cultured on uncoated (bottom) or fibronectin-coated (top) substrates. **(Right)** Corresponding mean diffusion coefficients of integrin β_1 and β_3 inside (iFAs) and outside (oFAs) focal adhesions obtained from fluorescence correlation spectroscopy (FCS). Values given as mean ± SEM; for uncoated, n = 100 FA sites for integrin β_1 and n=127 for β_3. For fibronectin, n = 110 FA sites for integrin β_1 and n=145 for β_3. (*$p < 0.01$ vs. iFAs; one-way ANOVA with post-hoc Tukey).

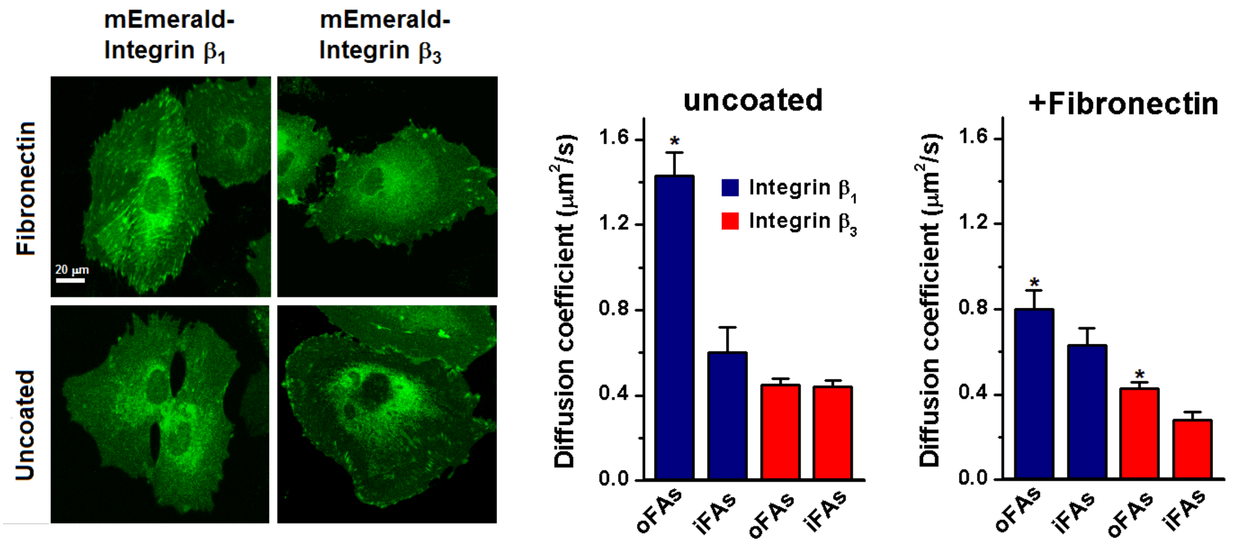

Supplementary Figure 41: Mobility of integrins in unpatterned SMCs. **(Left)** Representative images of SMCs transfected either with mEmerald-integrin β_1 or mEmerald-integrin β_3 and cultured on uncoated (bottom) or fibronectin-coated (top) substrates. **(Right)** Corresponding mean diffusion coefficients of integrin β_1 and β_3 inside (iFAs) and outside (oFAs) FAs obtained from FCS. Values given as mean ± SEM; for uncoated, n = 202 FA sites for integrin β_1 and n=263 for β_3. For fibronectin, n = 129 FA sites for integrin β_1 and n=143 for β_3. (*$p < 0.01$ vs. iFAs; one-way ANOVA with post-hoc Tukey).

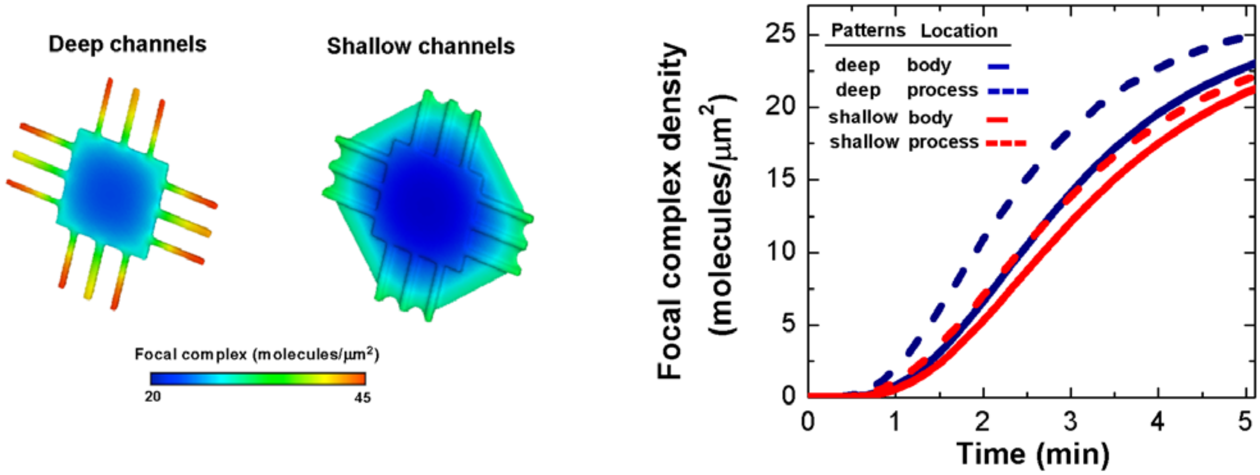

Supplementary Figure 42: Numerical simulations of micropatterned podocytes. **(Right)** Simulations using a partial differential equations-based model recapitulate the importance of 3-D shape whereby *in silico* cells that are in shallow micropatterns fail to generate spatial gradients in focal adhesion formation even though they are spread into channels in their basal surface. In contrast, *in silico* cells that are in deep micropatterns generate robust spatial gradients. **(Left)** Focal adhesion density at the membrane within cell body and the peripheral processes of simulated podocytes in deep or shallow micropatterns as a function of time.

Supplementary Tables

Uncoated	Mean diffusion coefficient ($\mu m^2/s$)			
	Integrin β_1 outside FAs	Integrin β_1 inside FAs	Integrin β_3 outside FAs	Integrin β_3 inside FAs
Podocytes	0.59±0.08	—	0.32±0.04	0.14±0.01
SMCs	1.43±0.11	0.6±0.12	0.45±0.03	0.44±0.03
MEFs[1]	—	—	—	—

Supplementary Table 1: Summary of diffusion coefficients of integrin β_3 and β_1 inside and outside FAs as measured for podocytes and SMCs plated on substrates without ECM coating (uncoated).

**+Fibronectin**	**Mean diffusion coefficient ($\mu m^2/s$)**			
	Integrin β_1 outside FAs	Integrin β_1 inside FAs	Integrin β_3 outside FAs	Integrin β_3 inside FAs
Podocytes	0.23±0.03	—	0.17±0.01	0.15±0.03
SMCs	0.8±0.09	0.63±0.08	0.43±0.03	0.28±0.04
MEFs[1]	0.3±0.005	0.09±0.005	0.29±0.004	0.08±0.002

Supplementary Table 2: Summary of diffusion coefficients of integrin β_3 and β_1 inside and outside FAs as measured for podocytes, SMCs, and MEFs plated on fibronectin coated substrates.

Cell	Type	Figure	Source
Podocytes	Human, kidney epithelial	1, 3, 4, 6, 7	[2]
HITB5 (SMC)	Human, internal thoracic artery	5, 6	Cellution Biosystems
A10 (SMC)	Rat, embryonic thoracic aorta	7	ATCC
Dermal fibroblasts	Human, foreskin	1	[3]
COS-7	Monkey, kidney fibroblasts	2	ATCC
Cardiac fibroblasts	Neonatal rat, heart	1	[4]
Foreskin fibroblasts	Human, foreskin	4	[3]

Supplementary Table 3: Summary of all cell types used in this study and their sources.

Antibody	Function	Source
Alpha actinin 4 (Rabbit)	IF 1:100	Abcam, cat # ab108198
Alpha smooth muscle actin (1A4, Mouse)	IF 1:100	*Abcam, cat #* ab7817
Calponin (EP798Y, Rabbit)	IF 1:100	*Abcam, cat #* ab46794
CD2AP (A599, Rabbit)	IF 1:50	*Cell Signaling, Cat #* 5478S
CD49e (NKI-SAM-1, Mouse)	IF 1:200	EMD Millipore, *cat #* CBL497
Collagen I	IF 1:200	*Abcam, cat #* ab34710
Fat1 (Rabbit)	IF 1:50	*Sigma, cat #* HPA023882
Fibronectin (Rabbit)	IF 1:200	*Sigma, cat #* F3648
Fyn (2A10, Rabbit)	IF 1:200	*Abcam, cat #* ab119855
Integrin beta 1 (P5D2, Mouse)	IF 1:200	*Abcam, cat #* ab24693
Integrin beta 3 (BV4, Mouse)	IF 1:100	*Abcam, cat #* ab7167
Integrin alpha V (LM142, Mouse)	IF 1:100	EMD Millipore, *cat #* MAB1978
Integrin beta 3 (B3A, Mouse)	Blocking 1:100	EMD Millipore, *cat #* MAB2023Z
Integrin beta 1 (6S6, Mouse)	Blocking 1:100	EMD Millipore, *cat #* MAB2253
Laminin (Rabbit)	IF 1:20	*Sigma, cat #* L9393
Nephrin (Ig-like domain 5, Rabbit)	IF 1:100	*Enzo, cat # 810-016-R100*
KIRREL (Rabbit)	IF 1:50	Sigma, cat # HPA030458
Phospho-FAK (pTyr397, Rabbit)	IF 1:50	*Cell Signaling, cat #* 3284S
Phospho-myosin (Ser19, Mouse)	IF 1:100	*Cell Signaling, cat #3675L*
Phospholipase C-epsilon-1 (Rabbit)	IF 1:50	*Sigma, cat #* HPA015598
Podocin (Rabbit)	IF 1:100	*Sigma, cat #* P0372
Synaptopodin (Mouse)	IF 1:50	*Progen Biotechnik, cat #* 65294
Vimentin (R28, Rabbit)	IF 1:50	*Cell Signaling, cat #* 3932S
ZO-1 (Rabbit)	IF 1:100	*Invitrogen, cat #* 40-2200

Supplementary Table 4: List of primary antibodies and dilutions used for immunofluorescence staining and functional blocking.

Supplementary Note 1: Reaction-Diffusion model

1.1 Derivation of analytical and computational framework

To address the question whether cell shape can modulate cellular phenotypes, we used partial differential equations to derive a reaction-diffusion model that quantified the effect of changing the cellular morphology on the spatial activation of membrane components in response to extracellular signaling.

The following biochemical reaction scheme is adopted: extracellular component A that is allowed to freely diffuse, interacts with a membrane component X to form an activated membrane component B at time $t = 0$, where:

$$A + X \rightleftarrows B \tag{1.1}$$

The initial distribution of the component X along the membrane is assumed to be uniform and its concentration $N_X[\text{molecules}/\mu\text{m}^2]$ is fixed with respect to time. B is a membrane component that is free to diffuse along the plane of the membrane with a diffusion coefficient $D_B[\mu\text{m}^2/s]$. Initially, the density of B is zero, and it is subject to degradation throughout the diffusion field with a degradation rate $k_d[1/\text{s}]$. The dynamics of A in the extracellular space is given by:

$$\frac{\partial C_A}{\partial t} = D_A \nabla^2 C_A \tag{1.2}$$

Where $C_A[\text{molecules}/\mu\text{m}^3]$ is the concentration of A in the extracellular space and $D_A[\mu\text{m}^2/s]$ is the diffusion coefficient of A. Similarly, the dynamics of B in the membrane is governed by:

$$\frac{\partial N_B}{\partial t} = D_B \nabla^2 N_B + k_{on} C_A N_X - \left(k_{off} + k_d\right) \cdot N_B \tag{1.3}$$

where $N_B[\text{molecules}/\mu\text{m}^2]$ stands for the concentration of B for any $t \geq 0$, $k_{on}[\mu\text{m}^3/\text{molecules} \cdot \text{s}]$ and $k_{off}[1/\text{s}]$ are the forward and backward reaction rate constants respectively. The boundary condition accounts for the balance between diffusive flux and reaction rate along the membrane is given by:

$$D_A(\text{n} \cdot \nabla C_A) = -k_{on} C_A|_{\partial\Omega} N_X + \left(k_{off} + k_d\right) \cdot N_B \tag{1.4}$$

where n is the unit vector normal at any point along the boundary, and $C_A|_{\partial\Omega}$ is the concentration of A at the membrane boundary. Additionally, N_B and N_X must satisfy periodic boundary conditions because the domain is closed.

The above equations were solved numerically in both 3-D and 2-D coordinate systems for all considered shapes (Supplementary Figures 5 and 43) using Matlab. For the model, a parabolic PDE of the below form has been adopted:

$$du_t - \nabla \cdot (c\nabla u) + au = f \tag{1.5}$$

with Neumann boundary conditions in the form:

$$\vec{n} \cdot (c(x,y,z)\nabla u) + q(x,y,z)u = g(x,y,z) \tag{1.6}$$

where $\vec{n}$ is a unit vector normal to the domain (directly outward, perpendicular to the tangent vector) and has a unit length. The volumetric domain was discretized into ~10^4 triangle elements and solved with a finite element solver with maximum allowable time-steps of 10 sec (tolerance of 0.01%). When a 2-D solution was adopted, equations 1.2-1.6 were reduced to their 2-D form (xy-plane) where $(x,y) \in \partial\Omega$. The cytoplasmic volume concentration C_A is reduced to a surface concentration $C_A[\text{molecules}/\mu\text{m}^2]$, the membrane surface densities N_X and N_B to line densities $N_X[\text{molecules}/\mu\text{m}]$ and $N_B[\text{molecules}/\mu\text{m}]$, and the forward reaction rate constant k_{on} is given by $k_{on}[\mu\text{m}^2/\text{molecules} \cdot \text{s}]$.

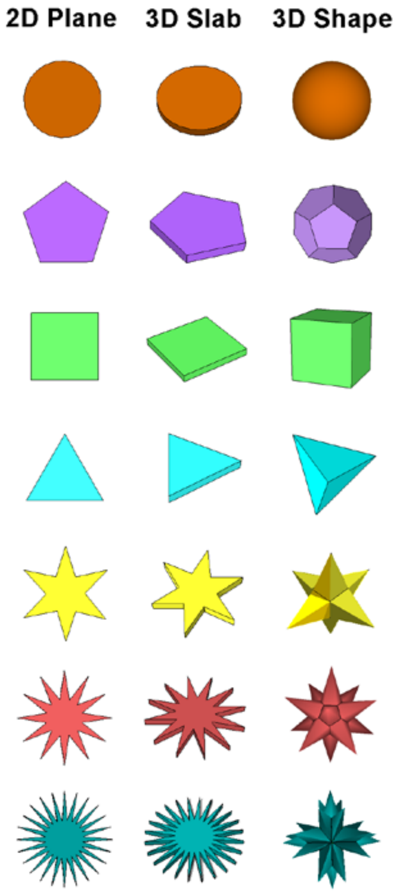

Supplementary Figure 43: Series of 2-D and 3-D geometries used to represent the distribution of shapes with a wide range surface area to volume ratios. For the 2-D plane geometries, a constant surface area of $2500\ \mu\text{m}^2$ is kept. For the 3-D slab geometries and 3-D shapes, a constant volume of $1.25 \cdot 10^5\ \mu\text{m}^3$ is kept for all shapes. Keeping the surface area and the volume constant allow us to generate a series of shapes with a linear surface-to-volume ratio.

1.2 Graphical solutions and biophysical interpretations

The 2-D geometries allow studying the effect of curvature variation along the membrane rather than the effect of volumetric reactions as taken into account in a 3-D system [5, 6]. The 3-D slab geometries stand mainly for the case of patterned cells, when a uniform height is kept. The full

3-D shapes demonstrate a more realistic case of *in vivo* cells that allow simulating the native volumetric balance between diffusive flux and reaction along the cellular membrane.

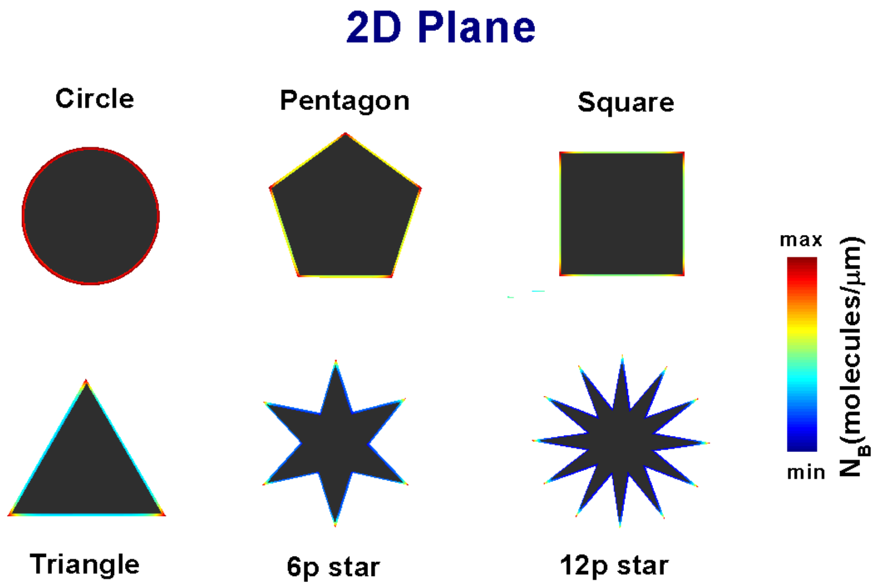

Supplementary Figure 44: 2-D plane models demonstrate the effect of membrane curvature on the distribution and of the membrane component B. The initial concentration of A in the extracellular space is 1 μM, and the initial density of B on the membrane is 0 molecules/μm.

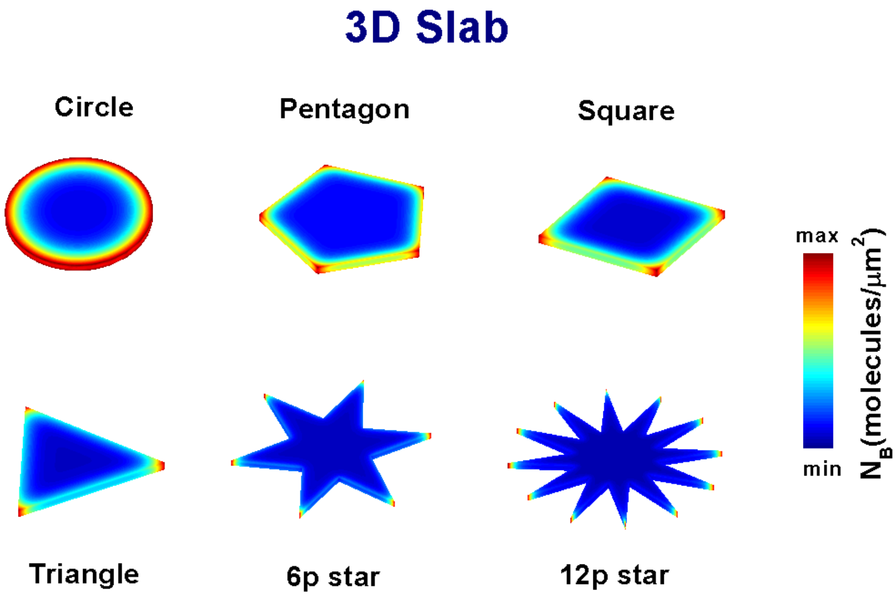

Supplementary Figure 45: 3-D slab models demonstrate the effect of membrane surface curvature on the distribution of the membrane component B. The initial concentration of A in the extracellular space is 1 μM, and the initial density of B on the membrane is 0 molecules/μm^2.

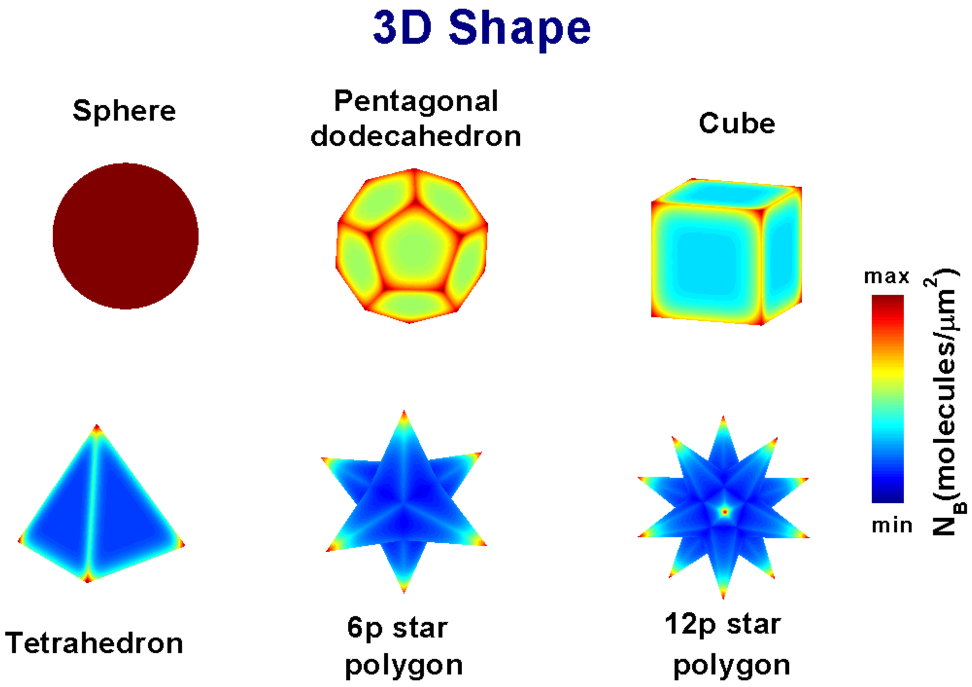

Supplementary Figure 46: 3-D shape models demonstrate the volumetric effect of membrane curvature on the distribution of the membrane component B. The initial concentration of A in the extracellular space is 1 μM, and the initial density of B on the membrane is 0 molecules/μm^2.

The 2-D and 3-D simulations presented in Supplementary Figures 44-46 are computed based on the solution of the reaction-diffusion equation 1.2. For all simulations, the volume component A is presented in the extracellular space and is free to interact with the cell membrane component X to activate the membrane component. The interaction depends on the global shape of the cell and on the local curvature of the membrane. The global diffusion and concentration values used to initiate the calculations are summarized in Supplementary Table 5.

For the circular 2-D geometry, there is no spatial variation of membrane component B along the membrane. However, as the geometry evolves, and the curvature of the membrane increases, the distribution of B becomes curvature dependent. Here, the concentration of B is higher at the tips than along the flat edges when a strong line gradient is observed. For the 3-D slabs, as the membrane curvature evolves, a strong gradient of B toward the center of the cell is observed where the highest distribution of B is found along the membrane sharp edges. Similarly, for the 3-D shapes, distinct membrane-domains of B built up on the volumetric tips as the morphology became more serrated. The density of B in the tips is 3-4 times higher compared to the density of B along the membrane in spherical geometry.

Generally, for serrated shapes with high membrane curvature (e.g. star polygon), the underlying mechanism for spatial specification is based on the dominancy of reaction over diffusion in membrane microdomains. In those areas, the relatively small contribution of diffusion is due to the limited volume of the domains[5]. As the membrane surface becomes sharper, the local volume decreases and the role of diffusion becomes less dominant, leading to a strong gradient buildup. Based on this, we can conclude that for more complex shapes, reaction has a stronger effect on

microdomain dynamics than diffusion regardless of the type of the cellular signaling. If the signal arises from interactions (i.e. reaction) between membrane bound components in the absence of a ligand, the high membrane curvature enhances reaction kinetics due to the decrease in the effect of diffusion. This finding suggests a mechanism for efflux of information from cell shape and indicates that cell shape can play an important role in shaping cellular phenotypes

Parameter	Value	Units	Compartment
k_{on}	1	$\mu M^{-1} \cdot s^{-1}$	Membrane
k_{off}	$5 \cdot 10^{-3}$	s^{-1}	Membrane
k_d	10^{-3}	s^{-1}	Membrane
$C_{A,t=0}$	1	μM	Extracellular space
$N_{X,t=0}$	10^{3}	$\text{molecules}/\mu m^2$	Membrane
$N_{B,t=0}$	0	$\text{molecules}/\mu m^2$	Membrane
D_A	1	$\mu m^2/\text{sec}$	Extracellular space
D_B	0.1	$\mu m^2/\text{sec}$	Membrane

Supplementary Table 5: Summary of global parameters used for reaction-diffusion simulations. Although all parameters are given in 3-D notations, same values were also used for the 2-D simulations with their matched units as defined in section 2.1. For simplicity, for all volumetric terms, we use molar representation instead of the number of molecules per cubic micrometer. The transformation between the two terms is given by, for 3-D: 1 μM $= 6.02 \cdot 10^{26}$ $\text{molecules}/\mu m^3$, and for 2-D: 1 μM $= 6.02 \cdot 10^{22}$ $\text{molecules}/\mu m^2$.

Supplementary Note 2: Cellular activation model

2.1 Derivation of analytical framework

Here, we derive the model for the cellular activation based on shape and tension signals, given in first section of our paper, in order to understand how they affect the maturation of phenotype as illustrated in Supplementary Figure 47. We assume two independent types of membrane receptors that can be activated by either force or shape signal, and theoretically calculate the activation rate of the receptors for varying 3-D shapes as shown in Supplementary Figure 43.

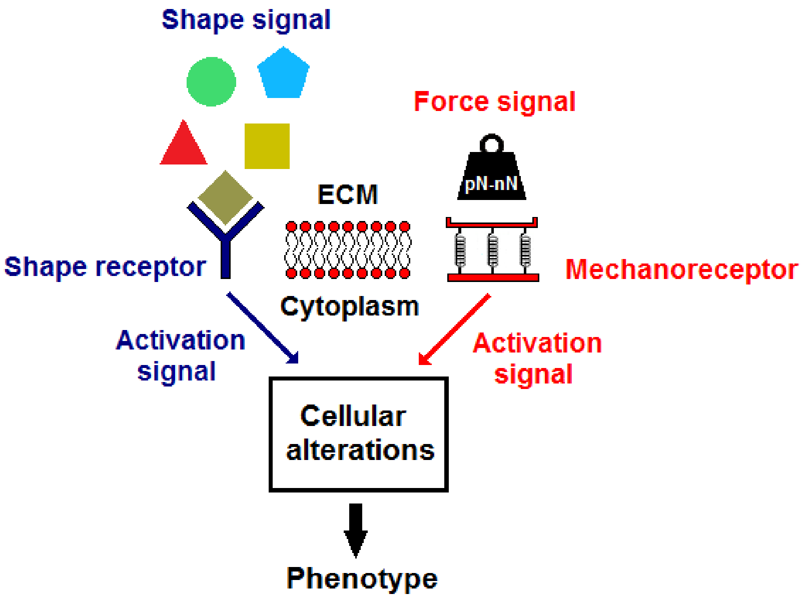

Supplementary Figure 47: Illustration of activation model. Both shape and force signals are assumed to contribute to the overall cellular phenotype by inducing specific cellular alterations. The model aims to clarify how the contributions of these two signals change with respect to cell shape. We use activation rates of the receptors as a measure to demonstrate the relative contribution of each.

Assuming the following reaction occurs on the cell membrane:

$$A + X \rightleftarrows X^* \tag{2.1}$$

where A, X, X^* stand for the concentrations of the extracellular ligand, inactive, and active membrane receptors respectively. The dynamic of X^* in the membrane is given by its corresponding rate equation:

$$\frac{dN_{X^*}}{dt} = k_{on}C_A N_X - k_{off}N_{X^*} \tag{2.2}$$

where C_A[molecules/μm^3] is the concentration of A in the extracellular space, N_X[molecules/μm^2] and N_{X^*}[molecules/μm^2] are the membrane concentrations of X and X^*, and k_{on}[μm^3/molecules · s], and k_{off}[1/s] are the forward and backward reaction rate constants respectively.

For long-term cell culture (days), such as those in this study, it is reasonable to assume that the biochemical ligand-receptor reaction has reached equilibrium, meaning that the number of activated receptors is fixed with respect to time; hence, the reaction rate $r = dX^*/dt$ equals zero. Using steady-state, the ratio of activated receptors X^* to the total receptors $X_T = (X + X^*)$, is given by:

$$\frac{X^*}{X_T} = \frac{C_A}{1/K_a + C_A} \quad (2.3)$$

where $K_a[\mathrm{M}^{-1}]$ is the association rate constant defined as:

$$K_a = \frac{k_{on}}{k_{off}} \quad (2.4)$$

Next, we drive the expressions for the association rate K_a as a function of the adopted shape morphologies presented in the Supplementary Figure 43. We considered shape and tension-driven mechanisms, which represent the response of the membrane receptors to geometrical and physical cues.

2.2 Shape-driven model

2.2.1 Surface-to-volume effect

Shape effect mainly operates by modulating the effective membrane area versus the cytoplasmic volume, also known as the surface-area-to-volume ratio (SA/V). The reaction rate of many biochemical processes are affected by the SA/V ratio and cells with high SA/V ratio will react faster because more membrane surface is available to react[6]. High SA/V ratio provides a strong driving mechanism to accelerate biochemical processes by minimizing the free energy. From a biological point of view, increased cellular surface area leads to increased rate of reaction. A larger surface area per volume ratio increases the areas of contact between ligand-receptor pairs, meaning that the larger the membrane surface compared with the cytoplasmic volume, the faster the rate of reaction and hence the probability of ligand-receptor binding.

	2-D Plane			3-D Slab			3-D Shape		
**Shape**	**Circum. [μm]**	**Area [μm^2]**	**CS/A [μm^{-1}]**	**Area [μm^2]**	**Volume [μm^3]**	**SA/V [μm^{-1}]**	**Area [μm^2]**	**Volume [μm^3]**	**SA/V [μm^{-1}]**
sphere	176	2500	0.070	5408	7500	0.721	12090	$1.25\cdot10^5$	0.096
pentagon	190	2500	0.076	5564	7500	0.742	13279	$1.25\cdot10^5$	0.106
square	200	2500	0.080	5600	7500	0.746	15000	$1.25\cdot10^5$	0.120
triangle	228	2500	0.091	5681	7500	0.757	18014	$1.25\cdot10^5$	0.144
6p star	336	2500	0.134	6122	7500	0.816	25134	$1.25\cdot10^5$	0.201
12p star	697	2500	0.278	7099	7500	0.946	52279	$1.25\cdot10^5$	0.418
24p star	1332	2500	0.533	9040	7500	1.205	99330	$1.25\cdot10^5$	0.794

Supplementary Table 6: C/SA and SA/V ratios for all considered geometries

The C/SA (circumference-to-surface area) and SA/V ratios for the geometries presented in Supplementary Figure 43 are given in Supplementary Table 6 and summarized graphically in Supplementary Figure 48. For the 2-D plane geometries, a constant surface area of 2500 μm^2 is kept. For the 3-D slab geometries, and the 3-D shapes, a volume of $1.25 \cdot 10^5$ μm^3 is kept constant. Keeping surface area or volume constant allows generation of a series of shapes with a linear circumference-to-surface or surface-to-volume ratio as shown in Supplementary Figure 49.

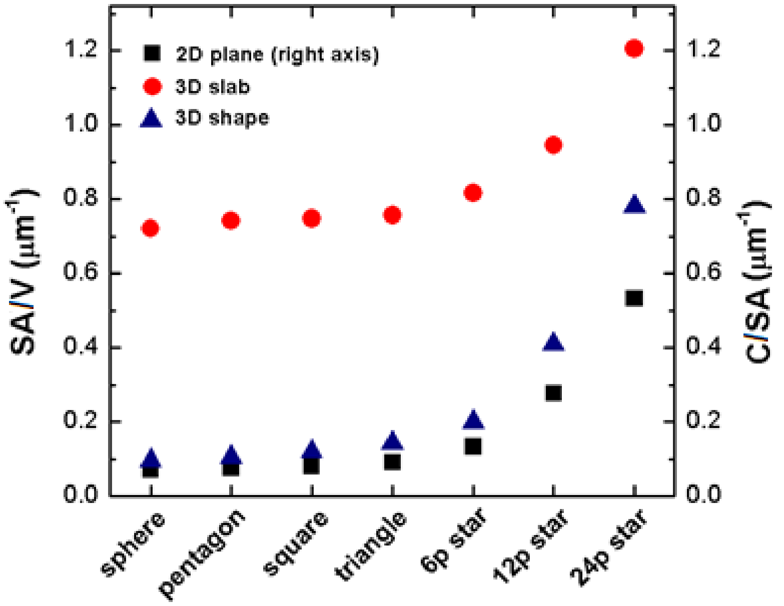

Supplementary Figure 48: Evolution of C/SA and SA/V ratios as function of 2-D and 3-D geometries respectively.

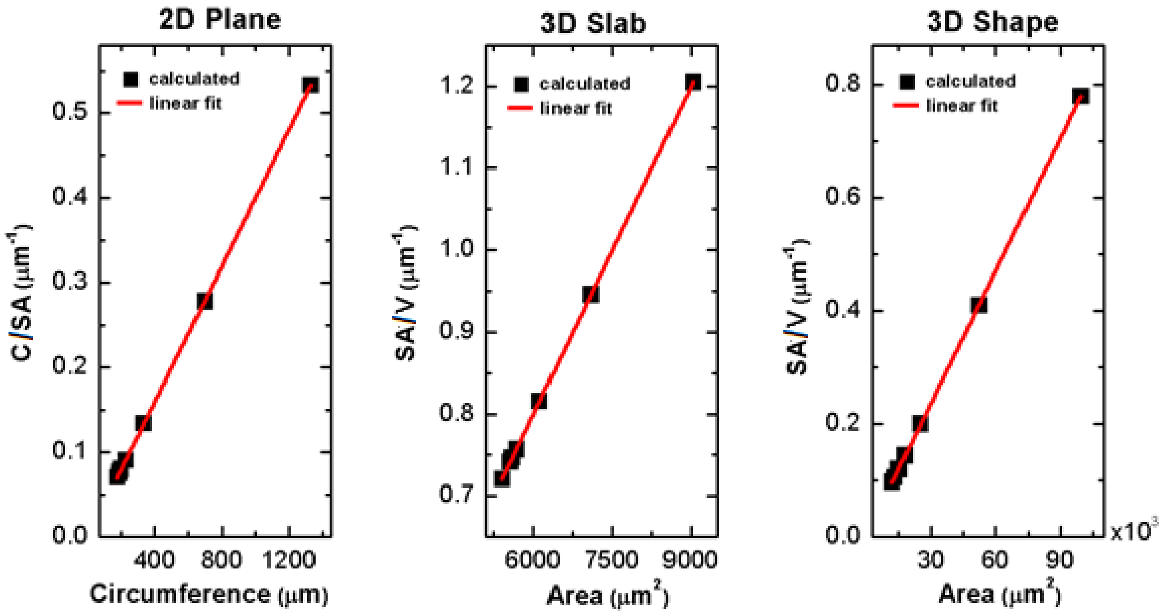

Supplementary Figure 49: C/SA and SA/V ratios as functions of surface area and volume. All parameters are listed in Supplementary Table 6 and are based on the geometries shown in Supplementary Figure 43.

2.2.2 Analytical definition of shape-dependent rate constant

For bounded chemical system, as in the case of shelled cells, the SA/V ratio affects the chemical reaction rate when employing a geometric model with isotropic properties. Here we consider the case of a second order biochemical reaction (Eq. 2.1) occurring on the membrane (surface of the considered shape) of 3-D volumetric shapes as shown in Supplementary Figure 43 (right column). The given 3-D shapes allow simulating a realistic volumetric space to study how the kinetics of a biochemical reaction is affected by cell shape. For a heterogeneous reaction, uninfluenced by mass transfer effects, the reaction is characterized by the specific forward rate constant $k_{on}(S_V)$[5] which takes into account the morphological variations of the edges and is given by:

$$k_{on}(S_V) = k_{on}\eta \tag{2.5}$$

where S_V stands for a given SA/V ratio, k_{on} is the native forward rate constant of the reaction and η is the effectiveness factor which is a dimensionless quantity defined as the ratio of the rate of reaction onto the shaped membrane to the rate at which reaction occurs in a continues unbounded space. The effectiveness factor accounts for the effect which the membrane curvature and cell morphology have on the chemical reaction rate and is given by:

$$\eta = \frac{\tanh \phi}{\phi} \tag{2.6}$$

where ϕ is a dimensionless quantity known as the Thiele modulus which describes the ratio between the diffusion and reaction components within a reaction-diffusion system. Generally, when $\phi \ll 1$, diffusion presents a negligible resistance while reaction is dominant. In contrast, when $\phi \gg 1$, the diffusion effect is dominant over reaction.

For a given closed boundary, the Thiele modulus is given by:

$$\phi = \frac{1}{S_V} \sqrt{\frac{k}{D_e}} \tag{2.7}$$

where S_V is the SA/V ratio, k $[1/\mathrm{s}]$ is the specific rate constant, and D_e $[\mu\mathrm{m}^2/\mathrm{s}]$ is the effective diffusion constant. Respectively, the modified shape dependent association rate constant $K_a(S_v)$ for any given SA/V ratio is given by:

$$K_a(S_V) = \frac{k_{on}(S_V)}{k_{off}} = S_V \cdot \frac{k_{on}}{k_{off}} \cdot \frac{\tanh\left(\frac{1}{S_V}\sqrt{\frac{k}{D_e}}\right)}{\sqrt{\frac{k}{D_e}}} \tag{2.8}$$

The integration of the Thiele modulus into the $K_a(S_V)$ term represents the change in the forward reaction rate in response to the geometrical constrains that shaped the cell boundary (the plasma membrane).

The physical basis for the differential distribution along the cell boundary is governed, respectively, by the biochemical and biophysical processes that are represented by the reaction-diffusion components. The process of free diffusion works toward homogenizing concentration gradients; i.e., Fickian diffusion eliminates concentration gradients over a length scale. On the other hand, chemical reactions are occurring everywhere along the cell membrane, and the local surface-to-volume ratio establishes concentration differences along the membrane. The balance between these two processes is represented by the Thiele modulus as shown graphically in Supplementary Figure 50. The Thiele modulus decreases exponentially with the increased SA/V ratio, which means that the reaction rate shows dominancy over diffusion. The distribution of the activated receptor fraction (represented by localization ratio) along the membrane is shown to follow precisely after the Thiele modulus. The localization rate sharply increases as the Thiele modulus gets smaller (reaction rate increases). At high SA/V, the reaction to diffusion ratio is nearly constant; hence, the localization phenotype reaches a plateau.

In addition to the Thiele modulus, the effectiveness factor η is used to describe how well the biochemical system responds to the shape constrains. As shown in Supplementary Figure 51, the local reaction rate along the boundary depends strongly on the local curvature (given by SA/V ratio) and found to growth sharply with the increased SA/V. However, as the SA/V becomes very high, the reaction rate becomes nearly constant meaning that further increase in the membrane curvature can not affect the reaction rate, hence, the biochemical response is fixed in respect to the morphological constraints.

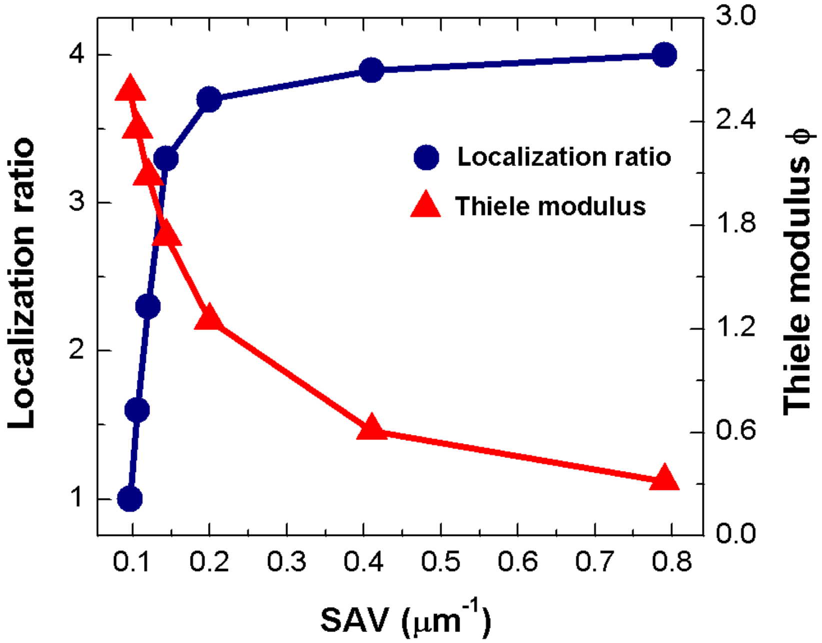

Supplementary Figure 50: Thiele modulus and localization ratio as function of SA/V ratio. The Thiele modulus values were calculated analytically and are based on the 3-D shapes presented in Supplementary Figure 43 ($D_e = 0.08$ $\mu m^2/s$, $k = 5 \cdot 10^{-3}$ $1/s$) . The localization ratio was computed based on the surface distribution of the activated receptors on the membrane and represents the ratio of densities between the edges and the center.

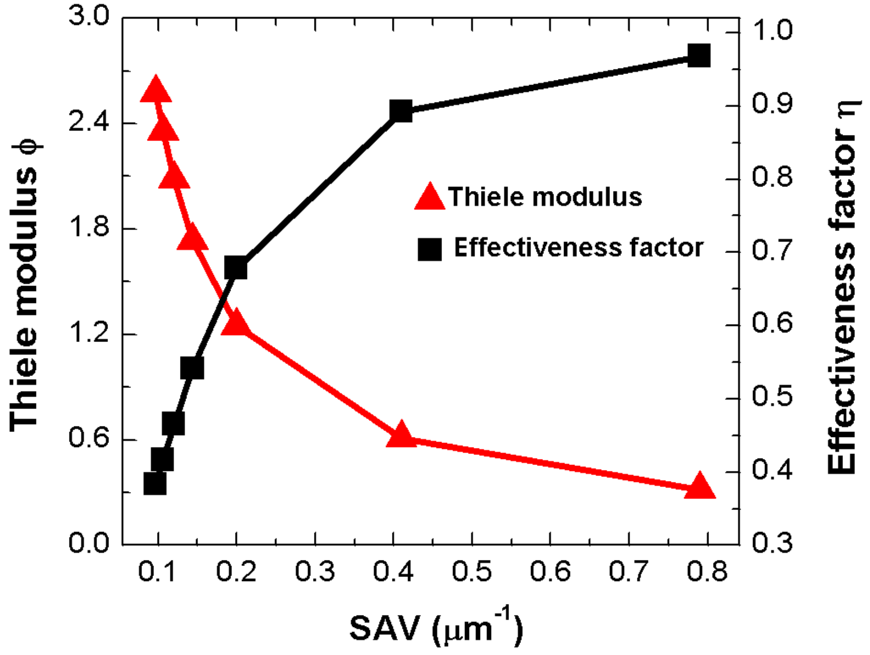

Supplementary Figure 51: Thiele modulus and effectiveness factor as function of SA/V ratio. The values were calculated analytically and are based on the 3-D shapes presented in Supplementary Figure 43 ($D_e = 0.08\ \mu\mathrm{m}^2/\mathrm{s}\ , k = 5 \cdot 10^{-3}\ 1/\mathrm{s}$).

2.3 Tension-driven model

2.3.1 Introduction and definitions

Here, we model the effect of cellular forces on the activation of tension-activated mechanoreceptors. The main goal of the model is to show how endogenous (internal) forces activate membrane mechanoreceptors in response to the evolving cellular shape. The activation of the receptors is then translated to changes in the force dependent association rate constant $K_a(f)$ as described in details at the next section under the title "catch-bond model". In our model, we assume that a constant contractile force $F_c[\mathrm{pN}/\mu\mathrm{m}^2]$ is present along the actin cytoskeleton in response to a mechanical load $Q[\mathrm{pN}/\mu\mathrm{m}^2]$. The load Q represents the net mechanical resistance due to applied forces (ECM, musculoskeletal, blood flow, blood pressure, etc.) against which the cell needs to react. Mechanical loads in cells are built particularly along the focal adhesions [7]. The cytoskeleton adheres to the focal adhesions from the inner side of the membrane and acts as a load-bearing network to allow transduction of mechanical signals throughout the cell [8]. Hence, the cytoskeleton bridges the extracellular space and the cytoplasm, allowing the transduction of mechanical signals in a fast and efficient manner. In response to the applied mechanical signals, cytoskeleton generates internal (endogenous) mechanical force, F_c, mostly through myosin ratcheting on actin filaments that allows the cell to mechanically balance the applied forces and to maintain its structural integrity. Endogenous forces are mainly contractile such that the cell homogeneously pulls its exterior towards its centroid. In contrast, applied forces are mainly directed outward from the cell [7, 9]. Hence, we can assume that the

directionality of the endogenous force, which is built in response to the resistance load Q, is in agreement with this biomechanical framework.

Tension, generated by the cytoskeleton in response to applied mechanical loads from the extracellular space, has been shown to be sufficient for mechanical activation of integrin mechanoreceptors [10]. Hence, endogenous forces, such as contractile tension generated by myosin motors, are primary used as an activation source [7]. It was shown by many studies that when myosin based contractility is inhibited (e.g., using blebbistatin), the activation of integrin mechanoreceptors is reduced dramatically, and that mechanotransduction due to applied forces is mostly lost [11, 12]. This suggests that the cell response to applied forces depends on the intact contractile activity of the actin cytoskeleton in order for the mechanoreceptors to generate a response.

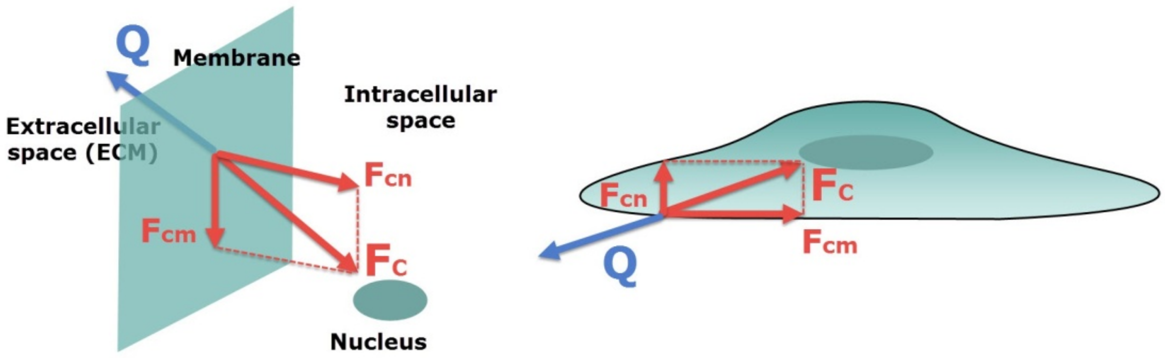

Supplementary Figure 52: Equilibrium of forces in a cell. (**Right**) Cells are exposed to multiple types of loads Q, such as shear and tensile forces acting through the extracellular space. The force generated by the contractile cytoskeleton F_c works to mechanically balance the applied forces and to maintain cellular integrity. (**Left**) Zoomed view of a single membrane bound focal adhesion where external and endogenous forces (F_c and Q) drive stress at the mechanoreceptor leading to increased activity and the initiation of signaling along the actin cytoskeleton.

2.3.2 Biophysical concept

For the modeling framework, we assumed that there is non-zero tension along the cytoskeleton in response to extracellular load acting on the cell membrane. This architecture acts as a network throughout the cell of cables under tension interconnected by the soft cellular membrane. The cables are under a constant force F_c to mechanically balance the applied load Q to maintain structural integrity. Here, we assume that the cytoskeletal force F_c is in equilibrium with the applied load. Generally, in cells, there are very few states of force disequilibrium that lead to acceleration (a 1 pN disequilibrium, less than the magnitude required to break most receptor-ligand bonds, would accelerate a cell at $\sim 1 \text{ m} \cdot \text{s}^{-2}$). In focal adhesions, the vector F_c is dissected to the tangent (F_{cm}) and normal (F_{cn}) vectorial components with respect to the cell membrane (Supplementary Figure 52). Due to the balance of applied and endogenous forces, the component F_{cm}, which lays in plane with the membrane, induces tensional stress in the focal adhesions (as also shown experimentally, see Refs.[7, 10, 13, 14]) leading to conformational changes that result in stress signaling response. Focal adhesions are highly dynamic structures that grow or shrink due to the turnover of their components in response to forces exerted by or through the surrounding

matrix[15]. This tension is known to induce specific mechanical response of tension-dependent receptors that are able to increase the affinity to their ligand when force is applied to the receptor-ligand bond[10] (also see Supplementary Figure 53). Thus, applied forces must be sensed by increased stress and must result in generation of internal forces at a similar magnitude. Under zero net force, the cell experiences stress and responds mechanically by deforming the membrane that in turn increases mechanotransduction by enhancing the receptor-ligand binding. Next, we used the 3-D shape geometries presented in Supplementary Figure 43 in order to calculate the fraction of force that acts on the plane of the membrane. The shear membrane force is then assumed to be directly related to the enhance activation of the mechanoreceptors.

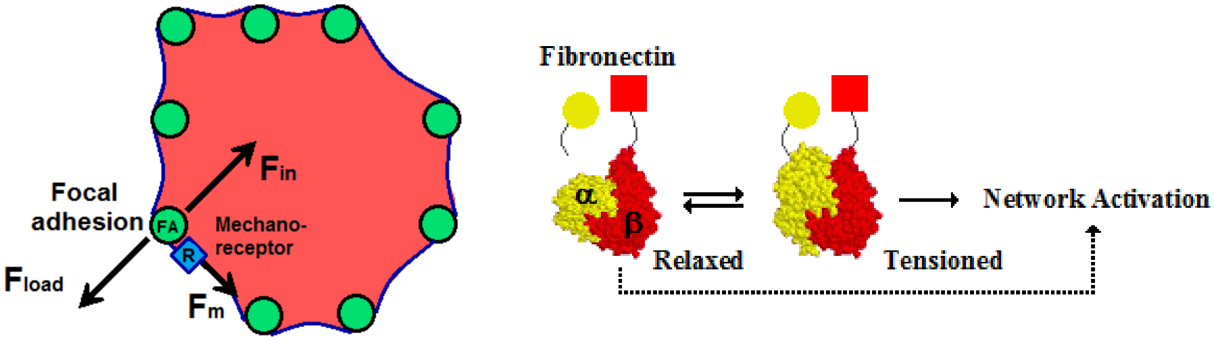

Supplementary Figure 53: Mechanism of force sensing and of tension-dependent receptors. **(Left)** External load is sensed by focal adhesions (FAs) that in response generate an internal force along the cytoskeleton (F_{in}). Due to the balance of forces across the FAs, the in-plane membrane component F_m induces a tensional force across the FA site and induces the activation of the mechanoreceptors. **(Right)** When force is applied to the mechanoreceptors, a conformational change occurs within the receptor site which allows enhancing the binding to its ligand. In turn, the stabilized receptor-ligand interaction enhances the activation of internal signaling.

2.3.3 Cellular measures

The model assumes 3-D cells in native tissue that experience continued forces mainly from the surrounding matrix[7, 16, 17]. Here, the applied load Q is entirely contributed by the tension extracted by the surrounding ECM which is the main source of mechanical stresses in tissues[14]. ECM driving forces are mainly tensile in nature and pull the cell outward against the plasma membrane[8, 16]. The threshold magnitude forces that trigger cellular responses appear to be in the pN to nN range per focal adhesion[10 18]. Typical loads generated by the ECM are usually characterized by a magnitude of few hundreds of piconewtons per unit area[7, 19]. This range of forces is in agreement with reported measurements of cell-generated contractile forces and show magnitudes from $0.1 - 1$ nN per focal adhesion under force equilibrium[9, 20]. Therefore, here we use a value of $200\ \mathrm{pN}/\mu\mathrm{m}^2$ to characterize the ECM load. Assuming a typical focal adhesion area of $2\ \mu\mathrm{m}^2$ (as also measured in this study for podocytes and SMCs), it yields a typical net force of ~0.4 nN per focal adhesion, which is satisfactory to allow the intact activation of mechanotransduction in cells[7].

Forces are not transmitted continuously throughout the entire cell membrane; this is because the membrane is not evenly anchored to the external matrix. Rather, the cell attaches to the external matrix by physically coupling its actin skeleton to the focal adhesion complexes onto the membrane which physically bridge between the inner and outer phases of the cells. Therefore, the model is fundamentally based on loads developed onto and sensed by focal adhesions (FAs),

hence, we must take into account a realistic arrangement of the FA sites on the cell membrane. Since the model is based on 3-D cellular morphologies, we can assume a similar case as showed in the pioneering work of Yamada and colleagues[17], where 3-D cells *in vivo* present multi-interactions with the surrounding environment. In this work, FA sites were found to be located throughout the whole membrane when the interactions with the ECM are not limited to the apical or basal projection of the cell as usually found in culture (standard *in vivo* culturing). Hence, here we assumed that the spatial distribution of FAs onto the membrane for each of the considered shapes is homogenous (constant distance between sites) where the site density was taken to be $0.1\ \mathrm{FAs}/\mu\mathrm{m}^2$ as measured previously[21]. The FA area was taken to be $2\ \mu\mathrm{m}^2$ and assumed to be constant throughout the whole membrane.

2.3.4 Analytical and computational methodology

To quantify the tensional force onto the adhesion sites, scalar integration of the filamentous tension component F_{cm}, over all focal adhesion sites onto the membrane was performed. Cartesian coordinates were built on the plane of a membrane to facilitate the integration (Supplementary Figure 54). The X-axis is defined to be in line with the edge and the Z-axis is normal to the membrane. The XY-*plane* defines the location of all points within the given membrane section. Since the vector F_{cm} lays on the in the XY-*plane*, we can dissect the vector to its vectorial components F_{cmx} and F_{cmy}, where: $\bar{F}_{cm} = \bar{F}_{cmx} + \bar{F}_{cmy}$.

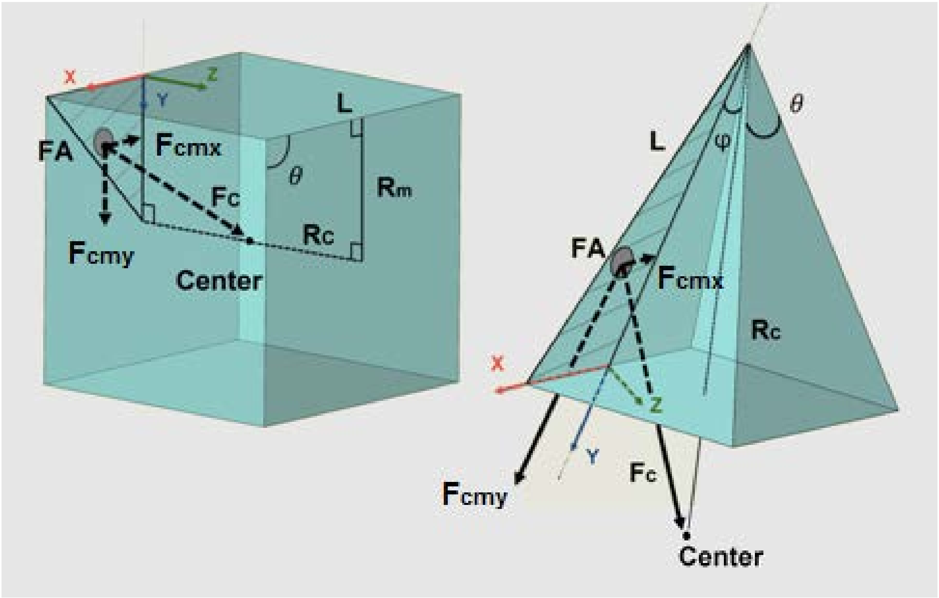

Supplementary Figure 54: Geometric definitions **(Left)** standard polyhedron geometry used for sphere, pentagon-dodecahedron, cube, and tetrahedron. **(Right)** stellated polyhedron used for 6-pointed, 12-pointed, and 24-pointed stars.

For a standard polyhedron geometry, which here includes icosahedron, pentagon-dodecahedron, cube and tetrahedron, the membrane section is considered as a superficial polygon. Respectively, the centroid is defined as: $(0, R_m, R_{in})$, where $R_m[\mu\mathrm{m}]$ is the inner radius of superficial polygon with an edge length defined as $L[\mu\mathrm{m}]$, and $R_{in}[\mu\mathrm{m}]$ is the inner radius of the polyhedron. For any given point on the membrane section $(x, y, 0)$, the vector F_{cm} that points towards the

centroid is denoted as $(-x, R_m - y, R_{in})$, the corresponding vectorial components F_{cmx} and F_{cmy} are expressed as:

$$F_{cmx} = |F_c| \frac{-x}{\sqrt{x^2 + (R_m - y)^2 + R_{in}^2}} \quad (2.9)$$

$$F_{cmy} = |F_c| \frac{R_m - y}{\sqrt{x^2 + (R_m - y)^2 + R_{in}^2}} \quad (2.10)$$

where $|F_c|$ is vectorial norm, R_{in}[μm] is the inner radius of the polygon, and R_m[μm] is the inner radius of superficial polygon defined as:

$$R_m = \frac{L}{2} \cdot tan\left(\frac{\theta}{2}\right) \quad (2.11)$$

where θ[rad] is the angle of the tip at the intersection of the edges, and L[μm] is the length of the polygon edge (geometrical parameters of polyhedron shapes are summarized in Supplementary Table 7).

**Shape**	$\boldsymbol{L}$**[μm]**	$\boldsymbol{R_{in}}$**[μm]**	$\boldsymbol{R_c}$**[μm]**	$\boldsymbol{\theta}$**[rad]**	$\boldsymbol{\varphi}$**[rad]**
sphere	38.5	29.1	-	1.05	-
pentagon	25.4	17.4	-	1.9	-
cube	50	25	-	1.6	-
Pyramid	102	20.8	-	1.05	-
6p star	65.9	-	72	0.44	0.13
12p star	65.9	-	81.2	0.23	0.16
24p star	65.9	-	81.9	0.35	0.1

Supplementary Table 7: Geometrical parameters use for regular polyhedrons and stellated polyhedrons.

For the case of stellated polyhedron (stars) geometry, built onto a standard polyhedron, which holds for 6-pointed, 12-pointed, and 24-pointed polyhedron, the membrane section is characterized as superficial side equilateral triangle and the centroid is given by the coordinates $(0, A, B)$. For any given point onto the membrane section$(x, y, 0)$, the vector F_{cm} that points towards the centroid is denoted as $(-x, A - y, B)$, the corresponding vectorial components F_{cmx} and F_{cmy} are expressed as:

$$F_{cmx} = |F_c| \frac{-x}{\sqrt{x^2 + (A - y)^2 + B^2}} \quad (2.12)$$

$$F_{cmy} = |F_c| \frac{A - y}{\sqrt{x^2 + (A - y)^2 + B^2}} \quad (2.13)$$

where:

$$A = R_c \cdot \cos(\varphi) - L \cdot \cos\left(\frac{\theta}{2}\right) \tag{2.14}$$

$$B = R_c \cdot \sin(\varphi) \tag{2.15}$$

where R_c[μm] is the circumradius radius of the stellated polygon with the edge length L[μm], θ[rad] is the angle between two adjacent edges, and φ[rad] is the angle between the circumradius and any given superficial side (geometrical parameters of stellated polyhedrons are summarized in Supplementary Table 7).

Since any two symmetrically distributed vectors F_{cmx} and F_{cmy} across the symmetric membrane section counterbalance each other (e.g. two force vectors on the symmetric positions across the midline of a triangular side), the scalar integration of F_{cmx} and F_{cmy} is only performed within the boundaries of the triangular bounded area. The shaded areas in Supplementary Figure 54 show examples of bounded membrane area where scalar integration is performed. The tension F_{FA} [N] generated onto a given FA site is then the product of this integration given by:

$$F_{FA} = \iint \left| F_{cmx} + F_{cmy} \right| dS \tag{2.16}$$

In order to calculate the average tensional force $\langle F_{FA} \rangle$, we calculated the force for all given FAs and then averaged by the total number of sites N (summarized in Supplementary Table 8), where:

$$\langle F_{FA} \rangle = \frac{1}{N} \cdot \sum_{i=1}^{N} F_{FA}^{i} \tag{2.17}$$

The average force as function of the evolving SA/V increases exponentially (Supplementary Figure 55). Since the mechanoreceptors are physically attached with the FA complexes, we assume that the force generated on the FA is transmitted entirely to activating receptors. Hence, these forces are taken as an input for the activation of the catch-bond model described in the next section. Calculated loads and forces for the FAs are summarized in Supplementary Table 8.

**Shape**	**Number of FAs**	**Mean force per FA $\langle F_{FA} \rangle$[pN]**	**Mean load per FA $\langle Q_{FA} \rangle$ [pN/μm^2]**	**Total membrane force F_M[nN]**	**Mean membrane load $\langle Q_M \rangle$ [pN/μm^2]**
sphere	120	148	74	18	1.48
pentagon	130	150	75	20	1.51
square	150	154	77	23	1.54
triangle	180	161	80	29	1.61
6p star	250	171	85	43	1.71
12p star	520	205	102	107	2.05
24p star	990	334	167	332	3.33

Supplementary Table 8: Calculated load and force values.

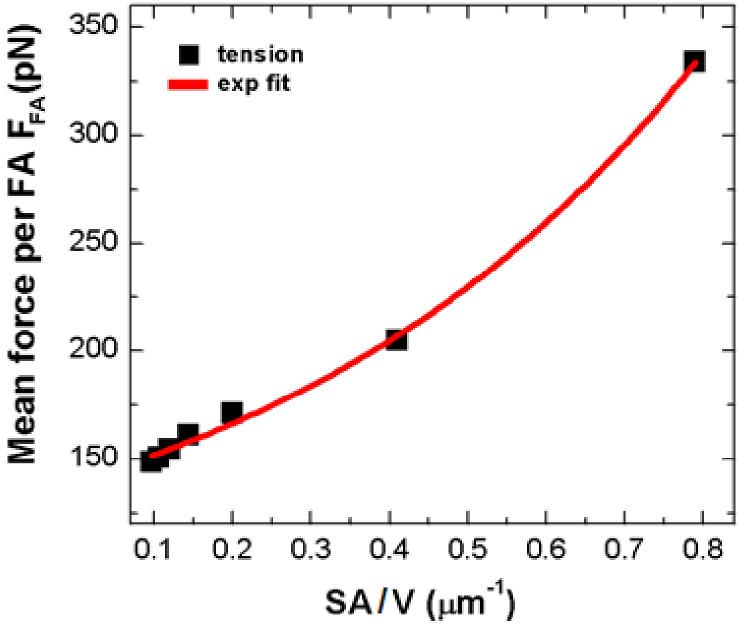

Supplementary Figure 55: Mean tension $\langle F_{FA} \rangle$ as function of SA/V ratio. The calculated force values are used as input for the activation of the catch-bond model.

2.4 Catch-bond model

Following the tension analysis, the interaction between the tensile force and the membrane mechanoreceptors X is described by adapting the catch-bond mechanism for cellular activation[22]. Catch bonds are used as mechanical clutches and known to respond to external loads or tensile forces. This mechanism allows the system to increase bond strength to initiate intercellular signals to control homeostasis. When a receptor-ligand interaction occurs on the membrane, the bond could be subject to mechanical forces. This is the case for anchoring bonds that are stretched in response to mechanical tension generated by the contractile cytoskeleton (endogenous force) and the ECM (applied force). This was shown to be sufficient for mechanical activation of many mechanoreceptors such as integrins[10].

Generally, under tensile mechanical force, bonds would slip apart (less stable) also known as "slip bonds"; however, it was shown that chemical bonds can become longer lived under tensile forces, leading to the term "catch bonds"[23]. Catch bonds allow cells to stabilize their receptor-ligand adhesion with the extracellular space when forces would otherwise pull the bonds apart. A practical example is integrins that are known to function as mechanoreceptors. Integrins are expressed on cells in an inactive from and show low affinity with slow on-rate (k_{on}) and fast off-rate (k_{off}) to their ligand; however, they become highly active under tension and show fast on and slow off rates[10, 24].

The effect of tensile forces on receptor-ligand interaction can be understood using the potential barrier ΔE_0 of the reaction. The larger is the height of the barrier separating the bound state from the dissociated state, the longer is the bond lifetime. Following Bell's model[25], applied force f[N] induces linear changes in the barrier height ΔE[m · N], when:

$$\Delta E(f) = \Delta E_0 \pm \Delta x \cdot f \tag{2.18}$$

where $\Delta x[\mathrm{m}]$ is the barrier width. The sign in front of the barrier width determines the direction of the force. If the sign is negative, the force pulls the ligand out, as shown by Bell. The situation describes slip bonds, since force promotes bond breaking. On the other hand, if the sign is positive, the force pushes the ligand into the receptor, and the complex behaves as a catch bond[23]. It should be noted however that under sufficient force, catch bonds would transition to slip bonds. There are several number of models commonly used to describe the catch-slip transition. In this study, we use the bond-deformation model[22, 26]. This model argues that force lowers the minimum potential energy by changing the receptor-ligand bond structure, and these conformational changes correlate with increased bond lifetimes. Generally, proteins can undergo structural changes during bond formation or when subjected to tensile forces. In the context of receptor-ligand, this can enhance or weaken the interactions. This motivates the functional form of the deformation energy $\Delta E[\mathrm{m} \cdot \mathrm{N}]$ given by:

$$\Delta E(f) = E_m \left(1 - \exp\left(\frac{-f}{f_0}\right)\right) \tag{2.19}$$

where $f[\mathrm{N}]$ is the applied tensile force, and $E_m[\mathrm{m} \cdot \mathrm{N}]$ is the energy limit when conformational saturation is achieved under the force $f_0[\mathrm{N}]$. Putting together equation 2.2 with the Bell term leads to the following off-rate constant $k_{off}(f)$, when:

$$k_{off}(f) = k_{off}^0 \cdot \exp\left[-\frac{\Delta E(f) - f \cdot \Delta x}{k_B \cdot T}\right] \tag{2.20}$$

where $k_{off}^0[1/\mathrm{s}]$ is the off-rate constant at zero force, $\Delta E[\mathrm{m} \cdot \mathrm{N}]$ is the energy barrier given by equation 2.19, $f[\mathrm{N}]$ is the applied tensile force, $\Delta x[\mathrm{m}]$ is the length difference between the equilibrium and the transition states, $k_B[\mathrm{m}^2 \cdot \mathrm{kg} \cdot \mathrm{s}^{-2} \cdot \mathrm{K}^{-1}]$ is the Boltzmann constant and $T[\mathrm{K}]$ is the temperature.

Following the general frame we developed in Equation 2.1-2.4 at the beginning of Supplementary Note 2, the modified tension-dependent association rate constant $K_a[\mathrm{M}^{-1}]$, which represents the ratio between on and off rates of the receptor-ligand interaction, is

$$K_a(f) = \frac{k_{on}}{k_{off}(f)} = K_a^0 \cdot \exp\left[\frac{\Delta E(f) - f \cdot \Delta x}{k_B \cdot T}\right] \tag{2.21}$$

where $k_{off}(f)$ is replaced with the term in equation 2.20, and $K_a^0 = k_{on}/k_{off}^0$ is the association rate constant at zero force. $K_a(f)$ is calculated independently for each of the 3-D shapes presented in Supplementary Figure 43. The parameters used for the calculations are summarized in Supplementary Table 9. The tensile force f associated with each of the shapes was taken based on equation 2.17, and it is assumed to represent a constant membrane tension that acts

uniformly on the mechanoreceptor X to induce force-dependent activation that enhances the generation of membrane fraction X^*.

Parameter	Value	Units
T – Temperature	298	K
k_B - Boltzmann constant	$1.38 \cdot 10^{-23}$	$m^2 \cdot kg \cdot s^{-2} \cdot K^{-1}$
Δx - Distance between states	5	Å
f_0 - Force limit	200	pN
E_m- Energy limit	400	Å · pN

Supplementary Table 9: Parameters used for the catch-bond model

Supplementary Note 3: Control theory model

3.1 Derivation of analytical framework

Here, we derive the solution for the optimal control problem, where we find a control parameter $\alpha(t)$ that allows minimization of time *t* to achieve a mature phenotype $\bar{X}^*(T) = \left[X_s^*(T), X_f^*(T)\right]$, where $X_s^*(T)$ and $X_f^*(T)$ represent the phenotypical contribution of the respective shape and tension signals to the overall phenotype. The optimal control problem is stated as:

$$\min_{\alpha(t)} \int_0^T dt \tag{3.1}$$

The dynamics of the variables are based on the scheme presented in Figure 2B in the main text, and they are mathematically described using the below differential equations:

$$\dot{X}_s^*(t) = \alpha(t)\beta\tilde{k}_s X_s^*(t)\left(\frac{c - X_s^*(t)}{c}\right) \tag{3.2}$$

$$\dot{X}_f^*(t) = \big(1 - \alpha(t)\big)\beta\tilde{k}_s X_s^*(t)\left(\frac{c - X_s^*(t)}{c}\right) + X_f^*(t)\big(\tilde{k}_f - \gamma\big) \tag{3.3}$$

where $k_s[1/s]$ and $k_f[1/s]$ are the rates by which the shape-dependent phenotype $X_s(t)$ and tension-dependent phenotype $X_f(t)$ are changed in respect to the activation of the cell by the extracellular cues; $\gamma[1/s]$ is the natural decay (degradation) rate of the phenotype, and β represents the contribution from outside-in biochemical signals. Biochemical signals are known to affect the way by which shape signals are translated by the cell[27]. Hence, biochemical cues can affect the rate by which the phenotype is changed with respect to the extracellular cues; therefore, β is used as a correction factor that allows adjusting the actual rates in the system. The term β ranges from 0 to 1. When $\beta = 0$, the biochemical dependence is maximal, hence, no shape effect is observed; when $\beta = 1$, the interdependence between shape and biochemical signals is insignificant, and the shape effect is independent of the chemical stimulation. *C* is the carrying capacity, which is the maximum sustainable phenotypic intensity that the cell can express. We assume that cellular phenotypes are bounded, meaning that any given phenotype cannot rise unrestrictedly and an upper level for the phenotypical appearance is always valid.

For simplicity we define:

$$\frac{X_s^*(t)}{c} = X_s(t)\,, \qquad \frac{X_f^*(t)}{c} = X_f(t)\,, \qquad \beta\tilde{k}_s = k_s\,, \qquad \big(\tilde{k}_f - \gamma\big) = k_f \tag{3.4}$$

The state equations become:

$$\dot{X}_s(t) = \alpha(t)k_s X_s(t)\big(1 - X_s(t)\big) \tag{3.5}$$

$$\dot{X}_f(t) = \big(1 - \alpha(t)\big)k_s X_s(t)\big(1 - X_s(t)\big) + X_f(t)k_f \tag{3.6}$$

where the control parameter $\alpha(t)$ must satisfy the constraint:

$$0 \leq \alpha(t) \leq 1 \tag{3.7}$$

We define:

$$\overline{\dot{X}}(t) = f\big(\bar{X}(t), \alpha(t)\big)\ ,\ \ \bar{X}(t) = \Big(X_s(t), X_f(t)\Big)^T \tag{3.8}$$

And the terminal manifold is given by:

$$\varphi[\bar{X}(t)] = \bar{X}(T) - \overline{X^{\#}} = 0 \tag{3.9}$$

where $X_s^{\#}$, $X_f^{\#}$ are the shape and tension-driven phenotypes at time $t = T$. At next stage, we define the Hamiltonian in the form:

$$H = \lambda_0 + \bar{\lambda}\bar{X}(t) = \lambda_0 + \lambda_1 X_s(t) + \lambda_2 X_f(t) \tag{3.10}$$

Using equations 3.5 and 3.6, we get:

$$H = \lambda_0 + (\lambda_1 - \lambda_2)\alpha k_s X_s(t)\big(1 - X_s(t)\big) + \lambda_2\big[X_f(t)k_f + k_s X_s(t)\big(1 - X_s(t)\big)\big] \tag{3.11}$$

Following Pontryagin's minimum principle, optimum control is obtained by minimizing H subject to the following conditions:

$$H\big[\bar{\lambda}(t), \overline{X^{\#}}(t), \alpha^{\#}(t)\big] = 0, \quad \lambda_0(t) = \text{Constant} \geq 0, \ \ t \in [0, T] \tag{3.12}$$

where $\overline{X^{\#}}(t)$ is the solution to equation 3.8. Based on equation 3.1 3, we define:

$$\sigma(t) = \lambda_1 - \lambda_2 \tag{3.13}$$

Equation 3.11 can be minimized by setting the value of $\alpha^{\#}(t)$ according to:

$$\alpha^{\#}(t) = \begin{cases} 0 & \sigma(t) > 0 \\ 1 & \sigma(t) < 0 \end{cases} \tag{3.14}$$

This solution is known as "bang-bang" and depends on the sign of the switching function $\alpha^{\#}(t)$.

The costate variables follow the costate equations:

$$\dot{\lambda}_1(t) = -H_{X_s} = -\sigma(t)\alpha k_s\big(1 - 2X_s(t)\big) - \lambda_2 k_s\big(1 - 2X_s(t)\big) \tag{3.15}$$

$$\dot{\lambda}_2(t) = -H_{X_f} = -\lambda_2 k_f \tag{3.16}$$

To calculate $\sigma(t)$ following equation 3.14, we have to consider the two possibilities $\alpha(T) = 0$ and $\alpha(T) = 1$.

Case I: at $t = T,\ \lambda_1(T) - \lambda_2(T) > 0$; and hence: $\alpha(T) = 0$. Since $\sigma(t) > 0$ on some terminal interval $[\tilde{t}, T]$, $\dot{\sigma}(t)$ must be positive for some $t \in [\tilde{t}, T]$, therefore:

$$\begin{aligned}\dot{\sigma}(T) = \dot{\lambda}_1(T) - \dot{\lambda}_2(T) &= -\sigma(T)\alpha k_s\big(1 - 2X_s(T)\big) - \lambda_2 k_s\big(1 - 2X_s(T)\big) + \lambda_2 k_f \\ &= \underbrace{-\sigma(T)\alpha k_s\big(1 - 2X_s(T)\big)}_{=0} - \lambda_2 k_s\big(1 - 2X_s(T)\big) + \lambda_2 k_f > 0\end{aligned} \tag{3.17}$$

From 3.12-3.14 we get:

$$\lambda_2(t) = \lambda_2(T)\mathrm{e}^{-k_f(t-T)} \tag{3.18}$$

$$\lambda_2(T) = \frac{-1}{k_f X_f(T) + k_s X_s(T)\big(1 - X_s(T)\big)} < 0 \tag{3.19}$$

and hence, from 3.17 we get:

$$k_s^* > k_f \ ,\ \ k_s^* = k_s\big(1 - 2X_s(t)\big) \tag{3.20}$$

Thus, $\sigma(t)$ will cross zero only when $k_s^* > k_f$ when a single switch occurs at $t = \tau$. From here we can show that:

$$\lambda_1(t) = \lambda_1(T) + \frac{k_s}{k_f}\big(1 - 2X_s(T)\big)\frac{1 - \mathrm{e}^{-k_f(t-T)}}{k_f X_f(T) + k_s X_s(T)\big(1 - X_s(T)\big)} \tag{3.21}$$

$$\sigma(t) = \lambda_1 - \lambda_2 = \lambda_1(T) + A\big(1 - \mathrm{e}^{-k_f(t-T)}\big) - \lambda_2(T)\mathrm{e}^{-k_f(t-T)} \tag{3.22}$$

where:

$$A = \frac{k_s\big(1 - 2X_s(T)\big)}{k_f\big[k_f X_f(T) + k_s X_s(T)\big(1 - X_s(T)\big)\big]} \tag{3.23}$$

From 3.22 at $t = \tau$, we can calculate the switching time:

$$\tau = T - \frac{1}{k_f}\ln\left(\frac{\lambda_1(\mathrm{T}) + \mathrm{A}}{\lambda_2(\mathrm{T}) + \mathrm{A}}\right) \tag{3.24}$$

The external control is given by:

$$\alpha(t) = \begin{cases} 1 & 0 < t < \tau \\ 0 & \tau < t < T \end{cases} \tag{3.25}$$

Integrating the state equations over the interval $0 < t < \tau$ yields:

$$X_s(t) = \frac{1}{1 + \left(\frac{1}{X_s(0)} - 1\right) \mathrm{e}^{-k_s t}} \tag{3.26}$$

$$X_f(t) = 0 \tag{3.27}$$

and for $\tau < t < T$:

$$X_s(t) = X_s(T) \tag{3.28}$$

$$X_f(t) = \frac{k_s}{k_f} X_s(T)(X_s(T) - 1)\left(1 - \mathrm{e}^{-k_f(t-\tau)}\right) \tag{3.29}$$

The switching time τ and the final time T are given by:

$$\tau = \frac{1}{k_s} \ln\left(\frac{X_s(0)}{X_s(T)} \cdot \frac{1 - X_s(T)}{1 - X_s(0)}\right) \tag{3.30}$$

$$T + \tau = \frac{1}{k_f} \ln\left(1 - \frac{k_f X_f(T)}{k_s X_s(T)(X_s(T) - 1)}\right) \tag{3.31}$$

Case II: at $t = T,\ \lambda_1(T) - \lambda_2(T) < 0$, and hence: $\alpha(T) = 1$. Since $\sigma(t) < 0$ on some terminal interval $[\tilde{t}, T]$, $\dot{\sigma}(t)$ must be negative for some $t \in [\tilde{t}, T]$, therefore:

$$\begin{aligned} \dot{\sigma}(T) = \dot{\lambda}_1(T) - \dot{\lambda}_2(T) = -\sigma(T)\alpha k_s\big(1 - 2X_s(T)\big) - \lambda_2 k_s\big(1 - 2X_s(T)\big) + \lambda_2 k_f \\ = -\lambda_1 k_s\big(1 - 2X_s(T)\big) + \lambda_2 k_f < 0 \end{aligned} \tag{3.32}$$

and since $\lambda_1(T) < \lambda_2(T)$, from equation 3.32 we get:

$$k_f > k_s^*\ ,\ \ k_s^* = k_s\big(1 - 2X_s(t)\big) \tag{3.33}$$

Thus, $\sigma(t)$ will cross zero only when $k_f > k_s^*$ when a single switch occurs at $t = \tau$. From here we can show that:

$$\lambda_1(T) = -\frac{\lambda_2(T) k_f X_f(T) + 1}{k_s X_s(T)\big(1 - X_s(T)\big)} \tag{3.34}$$

$$\lambda_1(t) = \lambda_1(T)\mathrm{e}^{-B(t-T)}\ , \qquad B = k_s\big(1 - 2X_s(0)\big) \tag{3.35}$$

at $t = \tau$, a switch occurs and $\lambda_1(\tau) = \lambda_2(\tau)$, using 3.18 the switching time τ is given by:

$$\tau = T + \frac{1}{B - k_f} \ln\left(\frac{\lambda_1(\mathrm{T})}{\lambda_2(\mathrm{T})}\right) \tag{3.36}$$

The external control is given by:

$$\alpha(t) = \begin{cases} 0 & 0 < t < \tau \\ 1 & \tau < t < T \end{cases} \tag{3.37}$$

Integrating the state equations over the interval $0 < t < \tau$ yields:

$$X_s(t) = X_s(0) \tag{3.38}$$

$$X_f(t) = \frac{k_s}{k_f} X_s(0)(X_s(0) - 1)\left(1 - \mathrm{e}^{-k_f t}\right) \tag{3.39}$$

and for $\tau < t < T$:

$$X_s(t) = \frac{X_s(0)}{X_s(0) - (X_s(0) - 1)\mathrm{e}^{-k_s(t-\tau)}} \tag{3.40}$$

$$X_f(t) = X_f(\tau)\mathrm{e}^{k_f(t-\tau)} \tag{3.41}$$

The switching time τ and the final time T are given by:

$$\tau = \frac{1}{k_f} \ln\left(1 - \frac{k_f}{k_s X_s(0)(X_s(0) - 1)} \cdot \frac{X_f(T)}{\mathrm{e}^{k_f(T-\tau)}}\right) \tag{3.42}$$

$$T - \tau = -\frac{1}{k_s} \ln\left(\frac{X_s(0) - \frac{X_s(0)}{X_s(T)}}{X_s(0) - 1}\right) \tag{3.43}$$

3.2 Estimation of control parameters: Nonlinear least squares (NLS) fit

Estimation of the control parameters from the measured data is necessary in order to obtain quantitatively accurate information regarding the phase transitions of the examined cells. A general and powerful method for data fitting involves nonlinear least squares (NLS) fit. The theory of least squares curve fitting assumes that the expected variances of the experimental observations will all be equal. Since this technique gives confidence intervals for both the real and imaginary values it allows exact calculations of the matched weighting factors. Purpose of weighting is to give a transformation of the data so as to ensure effective equality of the variances. The inclusions of weighting factors lead to a reduction in the confidence intervals for

the parameters. Although the reduction in error is a useful feature of the method, it is not a requirement.

NLS is an extension of ordinary least squares method. Achievement of a successful fit automatically ensures that the Kramers-Kronig relations hold. NLS fits avoid most of the weaknesses of known graphical methods since NLS fits both real and imaginary parts simultaneously and yields the parameters associated with all, rather than half, of the data. It also provides the confidence limits for all the estimated parameters, showing which parameters are well determined and which are deviated from the common range. The best fit parameters are generally determined by minimizing the sum of squares of the errors using a minimization function as given by:

$$S(\bar{P}) = \sum_{i=1}^{N} W_i \left[X_{fit}(t_i, \bar{P}) - X_{so}\right]^2 \qquad (3.44)$$

where X_{fit} and X_{so} stand for the fitted and the source spectrum respectively, N is the number of sampling points, W is the weighting factor related to the standard deviation by $1/\sigma^2$ and $\bar{P} = [P_1, P_2, P_3, \dots, P_K]$ stands for the K fitted parameters.

As in any nonlinear fitting procedure, there is a problem in ensuring that a local minimum found in the parameter space is the global minimum[28]. This problem becomes more serious when the number of unknown parameters is large. Generally, no algorithm can ensure that the minimum found is indeed the absolute; thus, it is very important to have a logical initial estimate that relies heavily on the native values of the fitted parameters. In this work, the minimizing of $S(\bar{P})$ has been performed using a slightly modified form of the Levenberg-Marquardt algorithm (LMA), which provides a numerical solution to the minimization problem over a space of parameters of the function[29]. LMA interpolates between the Gauss-Newton algorithm (GNA) and the method of gradient descent. LMA is more robust than GNA such that it finds a solution even if it starts very far off the final minimum in many cases; however, it tends to be slower than GNA.

3.3 Levenberg-Marquardt Algorithm (LMA)

Like other numeric minimization algorithms, the LMA is an iterative procedure. To start a minimization, an initial guess for the parameter vector $\bar{P}$ should be decided and marked accordingly as $\bar{P}_0$. $S(\bar{P})$ can then be expend in respect to $\bar{P}_0$ in the parameter space, when:

$$S(\bar{P}) = a + \bar{b} \cdot \bar{P} + \frac{1}{2} \bar{P} \cdot E \cdot \bar{P} + \cdots \qquad (3.45)$$

where:

$$\begin{cases} a = S(\bar{P}_0) \\ \bar{b} = \left.\dfrac{\partial S}{\partial P_i}\right|_{\bar{P}_0} \\ E_{ij} = \left.\dfrac{\partial^2 S}{\partial P_i \partial P_j}\right|_{\bar{P}_0} \end{cases}$$

where E is the Hessian matrix. When the size of $\bar{P}$ is relatively small, the first three terms in Equation 3.45 are a good approximation to $S(\bar{P})$ so the gradient can be given by:

$$\Delta S(\bar{P}) = \bar{b} + E \cdot \bar{P} \tag{3.46}$$

Defining $\bar{P}_{min}$ which brings $S(\bar{P})$ to its minimum, Equation 3.46 can be written as:

$$\Delta S(\bar{P}_{min}) = \bar{b} + E \cdot \bar{P}_{min} = 0 \tag{3.47}$$

Subtracting Equations 3.46 from 3.47 yields:

$$\bar{P}_{min} - \bar{P} = \delta\bar{P} = -E^{-1} \cdot \Delta S(\bar{P}) \tag{3.48}$$

Equation 3.48 represents the transformation from the trial parameters $\bar{P}$ to the minimizing parameters $\bar{P}_{min}$. In most cases, the following equality is chosen:

$$\delta\bar{P}_j = -a \cdot b_j \ , \qquad j = 1, \dots, K \tag{3.49}$$

Equations 3.48 can now be written as:

$$\sum_{i=1}^{K} \delta P_i E_{ij} = -b_j \ , \qquad j = 1, \dots, K \tag{3.50}$$

Equations 3.49 and 3.50 use as the basic equations for minimization of $S(\bar{P})$, when:

$$b_i = \sum_{i=1}^{N} W_i \left[X_{fit}(t_i, \bar{P}) - X_{so}\right] \cdot \frac{\partial X_{fit}}{\partial P_i} \tag{3.51}$$

$$E_{ij} = \sum_{i=1}^{N} W_i \cdot \frac{\partial X_{fit}}{\partial P_i} \cdot \frac{\partial X_{fit}}{\partial P_j} \tag{3.52}$$

where the second derivative terms are dismissed since they are small enough to be negligible. In practice, the inclusion of higher derivatives can destabilize the procedure of the iteration.

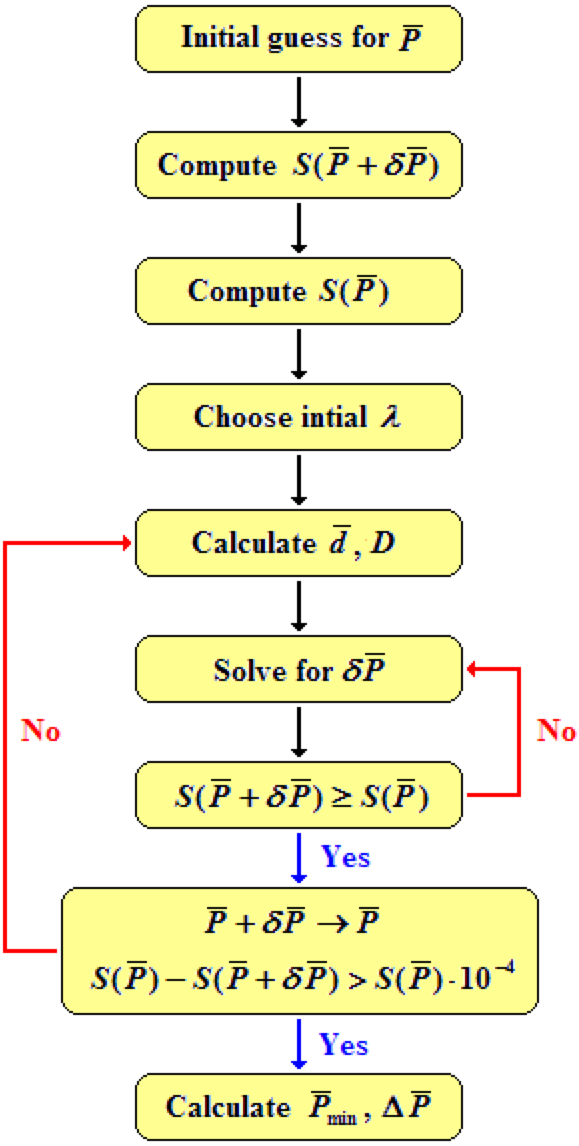

Supplementary Figure 56: Flow diagram of the Levenberg-Marquardt algorithm.

In order to find $\bar{P}_{min}$, equations 3.49 and 3.50 are transformed to:

$$\sum_{i=1}^{K} \delta P_i E'_{ij} = -b_j \ , \qquad j = 1, \dots, K \tag{3.53}$$

where:

$$\begin{cases} E'_{ij} = E_{ij}(1+\lambda)\,, \ i = j \\ E'_{ij} = E_{ij} \qquad\qquad , \ i \neq j \end{cases}$$

At each step, $\delta\bar{P}$ is solved by means of Gauss elimination with full pivoting. Once $\bar{P}_{min}$ is obtained, the confidence limits of the estimated parameters are calculated, when:

$$\Delta P_i = \pm\sqrt{C_{ii}} \tag{3.54}$$

where ΔP_i is the standard deviation and C_{ii} is the ith diagonal element of the covariance matrix $C = 2E^{-1}$, which gives information about the various parameters that are well determined. The overall algorithm and the iterative procedure is described by a block diagram and given in Supplementary Figure 56.

Supplementary Note 4: Signaling model

Integrin activation in different subcellular regions and the immediate downstream recruitment of active focal adhesion kinase, which initiates the focal adhesion formation, were modeled as a system of partial differential equations (PDEs) in three dimensions using the Virtual Cell suite (http://www.nrcam.uchc.edu/). Reversible binding reactions for integrin activation and focal complex formation were described by mass action kinetics, whereas the autophosphorylation of focal adhesion kinase by active integrin, activation of ERM, RhoGEF, RhoA and talin were approximated with irreversible Michelis-Menten kinetics.

Reversible reactions governed by mass action kinetics were characterized using PDEs for which binding of species *A* and *B* is described as:

$$\frac{\partial A}{\partial t} = D_A \nabla^2 A - k_1[A][B] + k_{-1}[AB] \tag{4.1}$$

where k_1 and k_{-1} are the forward and reverse reaction rate constants, respectively, and D_A is the diffusion coefficient of species *A*.

For enzyme-mediated reaction that are approximated by irreversible Michaelis-Menten kinetics, for which an enzyme *E* is operating on species *A*, reaction rates were described by:

$$\frac{\partial A}{\partial t} = D_A \nabla^2 A - \frac{k_{cat}[E][A]}{K_m + [A]} \tag{4.2}$$

where k_{cat} and K_m are the turnover number and the Michelis-Menten constant, respectively.

The kinetic parameters were calculated from experimentally measured literature values as referenced in Supplementary Table 10. The cell geometries for the shallow and deep channel micropatterns were approximated from experimentally observed morphologies as shown in Figure 1E in the main text. They were then discretized into 200 x 200 x 9 μm volumes with 404,010 elements of 1.0 μm isotropic step size and resolved with Sundials variable time-step CVODE stiff solver with maximum allowable time-steps of 100 ms.

Supplementary Table 10: Parameters for the compartmentalized partial differential equations-based model of integrin activation.

Reaction No	Reaction Name	Substrate *(type)*	Catalyst *(type)*	Product *(event)*	Km (µM)	Kcat (1/s)	Kf (1/µM.s)	Kr (1/s)	Biological Ref. Kinetic Ref.
**1**	Integrin activation by talin	Integrin_Off *receptor*	Talin_on *cytoskeletal*	Integrin_On *binding*			0.033	0.0042	[30] [31]
**2**	FAK auto-phosphorylation	FAK *kinase*	Integrin_On *receptor*	pFAK *phosphorylation*	6.7	0.9375			[32] [33]
**3**	ERM activation by Src	ERM *adaptor*	pFAK *kinase*	pERM *phosphorylation*	6.7	0.9375			[34] [33]
**4**	Formation of focal adhesion	pERM *kinase*	Integrin_On *receptor*	Focal_adhesion *binding*			0.033	0.0042	[35] [36]*
**5**	Positive feedback activating talin	Talin_off *cytoskeletal*	Focal_adhesion *complex*	Talin_on *enzymatic*			0.17	0.1	[37] [38]**
**6**	Activation of RhoGEF	RhoGEF *GEF*	pFAK *kinase*	pRhoGEF *phosphorylation*	6.7	0.9375			[39] [33]
**7**	Activation of RhoA	RhoGDP *GTPase*	pRhoGEF *GEF*	RhoGTP *enzymatic*	500	75			[40] [41]
**8**	Hydrolysis of RhoA	RhoGTP *GTPase*	RhoGAP *GAP*	RhoGDP *enzymatic*	1.79	1.61			[42] [43]
**9**	Localization of nephrin	U_Nephrin *membrane*	RhoGTP *GTPase*	Nephrin *phosphorylation*		10			[44, 45] ***

Molecule Name	Location Start	Location End	Diffusivity ($\mu m^2/s$)	Initial Concentration	Concentration Units	Conc. Ref. Diff. Ref.
Integrin (on/off)	*Membrane*	*Membrane*	*0.01*	0/180	(molecules/μm^2)	[46] [47]*
Talin (on/off)	*Cytoplasm*	*Membrane*	*1.0*	0.001/0.999	(µM)	[48]* [49]*
FAK (pFAK)	*Cytoplasm*	*Membrane*	*5.5*	1.0 (0)	(µM)	[48]* [49]*
ERM (pERM)	*Cytoplasm*	*Membrane*	*0.5*	1.0 (0)	(µM)	[48]* [49]*
RhoA (GTP)	*Cytoplasm*	*Cytoplasm*	*5.5*	0 (1.0)	(µM)	[48]* [49]*
RhoGEF (p)	*Cytoplasm*	*Cytoplasm*	*1.0*	1.0 (0)	(µM)	[48]* [49]*
RhoGAP	*Cytoplasm*	*Cytoplasm*	*1.0*	1.0 (0)	(µM)	[48]* [49]*
Nephrin (U)	*Membrane*	*Membrane*	*0.01*	0 (180)	(molecules/μm^2)	
Focal_Adhesion	*Cytoplasm*	*Membrane*	*0.0*	0	(molecules/μm^2)	

* estimates based on parameters for similarly sized proteins
** kinetics reflect activation through PIPKI and PIP2
*** assumed relationship; linearly translated with estimated time constant

Supplementary References

1. Rossier, O. *et al.* Integrins β1 and β3 exhibit distinct dynamic nanoscale organizations inside focal adhesions. *Nature cell biology* **14**, 1057-1067 (2012).

2. Saleem, M.A. *et al.* A conditionally immortalized human podocyte cell line demonstrating nephrin and podocin expression. *Journal of the American Society of Nephrology* **13**, 630-638 (2002).

3. Lee, E.J., Holmes, J.W. & Costa, K.D. Remodeling of engineered tissue anisotropy in response to altered loading conditions. *Ann Biomed Eng* **36**, 1322-1334 (2008).

4. Lee, E.J., Kim, D.E., Azeloglu, E.U. & Costa, K.D. Engineered cardiac organoid chambers: toward a functional biological model ventricle. *Tissue engineering. Part A* **14**, 215-225 (2008).

5. Peacock, D. & Richardson, J.F. *Chemical Engineering, Volume 3: Chemical and Biochemical Reactors and Process Control*, Vol. 3. (Butterworth-Heinemann, 1994).

6. Rangamani, P. *et al.* Decoding information in cell shape. *Cell* **154**, 1356-1369 (2013).

7. Chen, C.S. Mechanotransduction–a field pulling together? *Journal of cell science* **121**, 3285-3292 (2008).

8. Vogel, V. & Sheetz, M. Local force and geometry sensing regulate cell functions. *Nature reviews Molecular cell biology* **7**, 265-275 (2006).

9. Tan, J.L. *et al.* Cells lying on a bed of microneedles: an approach to isolate mechanical force. *Proceedings of the National Academy of Sciences* **100**, 1484-1489 (2003).

10. Friedland, J.C., Lee, M.H. & Boettiger, D. Mechanically activated integrin switch controls α5β1 function. *Science* **323**, 642-644 (2009).

11. Sarasa-Renedo, A., Tunç-Civelek, V. & Chiquet, M. Role of RhoA/ROCK-dependent actin contractility in the induction of tenascin-C by cyclic tensile strain. *Experimental cell research* **312**, 1361-1370 (2006).

12. Zhao, X.-H. *et al.* Force activates smooth muscle α-actin promoter activity through the Rho signaling pathway. *Journal of cell science* **120**, 1801-1809 (2007).

13. Trepat, X. *et al.* Universal physical responses to stretch in the living cell. *Nature* **447**, 592-595 (2007).

14. del Rio, A. *et al.* Stretching single talin rod molecules activates vinculin binding. *Science* **323**, 638-641 (2009).

15. Zamir, E. *et al.* Dynamics and segregation of cell–matrix adhesions in cultured fibroblasts. *Nature cell biology* **2**, 191-196 (2000).

16. Ingber, D.E. Tensegrity: the architectural basis of cellular mechanotransduction. *Annual review of physiology* **59**, 575-599 (1997).

17. Cukierman, E., Pankov, R., Stevens, D.R. & Yamada, K.M. Taking cell-matrix adhesions to the third dimension. *Science* **294**, 1708-1712 (2001).

18. Geiger, B., Spatz, J.P. & Bershadsky, A.D. Environmental sensing through focal adhesions. *Nature reviews Molecular cell biology* **10**, 21-33 (2009).

19. Orr, A.W., Helmke, B.P., Blackman, B.R. & Schwartz, M.A. Mechanisms of mechanotransduction. *Developmental cell* **10**, 11-20 (2006).

20. Balaban, N.Q. *et al.* Force and focal adhesion assembly: a close relationship studied using elastic micropatterned substrates. *Nature cell biology* **3**, 466-472 (2001).

21. Prager-Khoutorsky, M. *et al.* Fibroblast polarization is a matrix-rigidity-dependent process controlled by focal adhesion mechanosensing. *Nature cell biology* **13**, 1457-1465 (2011).

22. Thomas, W.E., Vogel, V. & Sokurenko, E. Biophysics of catch bonds. *Annu. Rev. Biophys.* **37**, 399-416 (2008).

23. Dembo, M., Torney, D., Saxman, K. & Hammer, D. The reaction-limited kinetics of membrane-to-surface adhesion and detachment. *Proceedings of the Royal Society of London B: Biological Sciences* **234**, 55-83 (1988).

24. Hynes, R.O. Integrins: bidirectional, allosteric signaling machines. *Cell* **110**, 673-687 (2002).

25. Bell, G.I. Models for the specific adhesion of cells to cells. *Science* **200**, 618-627 (1978).

26. Pereverzev, Y.V. & Prezhdo, O.V. Force-induced deformations and stability of biological bonds. *Physical Review E* **73**, 050902 (2006).

27. Neves, S.R. *et al.* Cell shape and negative links in regulatory motifs together control spatial information flow in signaling networks. *Cell* **133**, 666-680 (2008).

28. Marquardt, D.W. An algorithm for least-squares estimation of nonlinear parameters. *Journal of the society for Industrial and Applied Mathematics* **11**, 431-441 (1963).

29. Gill, P.E. & Murray, W. Algorithms for the solution of the nonlinear least-squares problem. *SIAM Journal on Numerical Analysis* **15**, 977-992 (1978).

30. Wegener, K.L. *et al.* Structural basis of integrin activation by talin. *Cell* **128**, 171-182 (2007).

31. Calderwood, D.A. *et al.* The phosphotyrosine binding-like domain of talin activates integrins. *The Journal of biological chemistry* **277**, 21749-21758 (2002).

32. Parsons, J.T. Focal adhesion kinase: the first ten years. *Journal of cell science* **116**, 1409-1416 (2003).

33. Withers, B.E., Keller, P.R. & Fry, D.W. Expression, purification and characterization of focal adhesion kinase using a baculovirus system. *Protein Expr Purif* **7**, 12-18 (1996).

34. Clucas, J. & Valderrama, F. ERM proteins in cancer progression. *Journal of cell science* **127**, 267-275 (2014).

35. Mitra, S.K., Hanson, D.A. & Schlaepfer, D.D. Focal adhesion kinase: in command and control of cell motility. *Nature reviews. Molecular cell biology* **6**, 56-68 (2005).

36. Welf, E.S., Naik, U.P. & Ogunnaike, B.A. A spatial model for integrin clustering as a result of feedback between integrin activation and integrin binding. *Biophys J* **103**, 1379-1389 (2012).

37. Martel, V. *et al.* Conformation, localization, and integrin binding of talin depend on its interaction with phosphoinositides. *The Journal of biological chemistry* **276**, 21217-21227 (2001).

38. Geier, F., Fengos, G. & Iber, D. A computational analysis of the dynamic roles of talin, Dok1, and PIPKI for integrin activation. *PloS one* **6**, e24808 (2011).

39. Hata, K., Kaibuchi, K., Inagaki, S. & Yamashita, T. Unc5B associates with LARG to mediate the action of repulsive guidance molecule. *J Cell Biol* **184**, 737-750 (2009).

40. Chikumi, H. *et al.* Homo- and hetero-oligomerization of PDZ-RhoGEF, LARG and p115RhoGEF by their C-terminal region regulates their in vivo Rho GEF activity and transforming potential. *Oncogene* **23**, 233-240 (2004).

41. Jaiswal, M. *et al.* Mechanistic insights into specificity, activity, and regulatory elements of the regulator of G-protein signaling (RGS)-containing Rho-specific guanine nucleotide exchange factors (GEFs) p115, PDZ-RhoGEF (PRG), and leukemia-associated RhoGEF (LARG). *The Journal of biological chemistry* **286**, 18202-18212 (2011).

42. Arthur, W.T. & Burridge, K. RhoA inactivation by p190RhoGAP regulates cell spreading and migration by promoting membrane protrusion and polarity. *Mol Biol Cell* **12**, 2711-2720 (2001).

43. Zhang, B. & Zheng, Y. Regulation of RhoA GTP hydrolysis by the GTPase-activating proteins p190, p50RhoGAP, Bcr, and 3BP-1. *Biochemistry* **37**, 5249-5257 (1998).

44. Wang, L. *et al.* Mechanisms of the proteinuria induced by Rho GTPases. *Kidney international* **81**, 1075-1085 (2012).

45. Lal, M.A. & Tryggvason, K. Knocking out podocyte rho GTPases: and the winner is. *Journal of the American Society of Nephrology : JASN* **23**, 1128-1129 (2012).

46. Wiseman, P.W. *et al.* Spatial mapping of integrin interactions and dynamics during cell migration by image correlation microscopy. *Journal of cell science* **117**, 5521-5534 (2004).

47. Gaborski, T.R., Clark, A., Jr., Waugh, R.E. & McGrath, J.L. Membrane mobility of beta2 integrins and rolling associated adhesion molecules in resting neutrophils. *Biophys J* **95**, 4934-4947 (2008).

48. Koh, G., Teong, H.F., Clement, M.V., Hsu, D. & Thiagarajan, P.S. A decompositional approach to parameter estimation in pathway modeling: a case study of the Akt and MAPK pathways and their crosstalk. *Bioinformatics* **22**, e271-280 (2006).

49. Arrio-Dupont, M., Foucault, G., Vacher, M., Devaux, P.F. & Cribier, S. Translational diffusion of globular proteins in the cytoplasm of cultured muscle cells. *Biophys J* **78**, 901-907 (2000).
