# Supplementary material for: Cell shape information is transduced through tension-independent mechanisms
Source: Nat Commun. 2017 Dec 15;8:2145. doi: 10.1038/s41467-017-02218-4 (PMC5732205; doi:10.1038/s41467-017-02218-4)
Supplement: Supplementary file 2 — Description of Additional Supplementary Files [file 41467_2017_2218_MOESM2_ESM.pdf]

Description of Additional Supplementary Files

File Name: Supplementary Data 1
Description: Differentially bound proteins for integrin β1 and β3 according to LC-MS/MS shotgun proteomics following IP.
